# Grandiflolines A–F, new anti-inflammatory diterpenoid alkaloids isolated from *Delphinium grandiflorum*

**DOI:** 10.3389/fchem.2022.1012874

**Published:** 2022-09-19

**Authors:** Yuanfeng Yan, Hongjun Jiang, Xiaoyan Yang, Zongbao Ding, Tianpeng Yin

**Affiliations:** Department of Pharmacy, Zhuhai Campus of Zunyi Medical University, Zhuihai, Guangdong, China

**Keywords:** delphinium grandiflorum, ranunculaceae, diterpenoid alkaloid, grandiflolines A-F, anti-inflammatory activity

## Abstract

*Delphinium grandiflorum* L. (family Ranunculaceae), one of the most important and widely distributed *Delphinium* species, has received considerable interest due to its extremely high medicinal value. The discovery of novel metabolites from *D. grandiflorum* supported and broadened its application as an herbal medicine. In this study, the whole herb of *D. grandiflorum* was phytochemically investigated to obtain fourteen C_19_-lycaconitine-type diterpenoid alkaloids (**1**–**14**), including six undescribed alkaloids, grandiflolines A–F (**1**–**6**). The structural elucidation of them was accomplished by detailed spectroscopic analyses, mainly including HR-MS, 1D and 2D NMR (^1^H–^1^H COSY, NOESY, HMBC and HSQC), and IR spectra. New alkaloids **1**–**3** and **5** possess a characteristic △^2,3^ functional group in the A ring, while compounds **5** and **6** feature a rare OH-16 substituent. In addition, known compounds **7**–**12** were isolated from *D. grandiflorum* for the first time. Moreover, according to its medicinal use, new alkaloids **1**–**6** were estimated for their potential *in vitro* anti-inflammatory effects, and some of them exhibited inhibitory effects on NO production in LPS-activated RAW 264.7 macrophages. Our work enriched the chemical diversity of *D. grandiflorum* and the genus *Delphinium* and presented beneficial information for further investigations.

## Introduction

The genus *Delphinium* L., which belongs to the tribe *Delphineae* in the family Ranunculaceae, is an important species-rich genus comprising approximately 400 species of annual, biennial, or perennial herbs. *Delphinium* plants prefer cold and humid conditions and are mainly distributed in mountainous regions in the north temperate zone, including Asia, Europe, North America, and sporadically North Africa (Wang, 2019). China is regarded as the distribution center for this genus, as more than half of the confirmed *Delphinium* species were reported to be grown within this country (232 species, 200 endemic), mainly in the high mountain areas in northern Yunnan, eastern Tibet and western Sichuan (Chen et al., 2009; Wang, 2019). *Delphinium* plants are well-known ornamental plants with a long history around the world (Yin et al., 2020). Many *Delphinium* species, represented by *D. grandiflorum*, *D. elatum*, *Delphinium × belladonna*, feature showy flowers with various colors, including white, pink, blue, light blue, violet, purple, and lavender, which are particularly popular worldwide and have been widely cultivated as landscape and potted plants or cut flowers (Ichimura et al., 2009). On the other hand, in many countries and regions, such as China and India, *Delphinium* plants are commonly used as herbal medicines by natives for treating various diseases, mainly traumatic injury, enteritis, rheumatism, headache, toothache, neuralgia, and other kinds of pain. The multiple therapeutic effects of *Delphinium*-derived herbs could be attributed to the abundance of various active ingredients, including diterpenoid alkaloids (DAs), flavonoids, phenolic acids, and volatile oils (Marin, 2011). In particular, DAs, which have been acknowledged as the characteristic ingredients for this genus, have exhibited broad-spectrum biological activities, including analgesic, anti-inflammatory, antiarrhythmic, anticancer, antioxidant, and neuroprotective activities (Thawabteh et al., 2021; Liu et al., 2022). Further exploration and discovery of bioactive DAs with novel structures from *Delphinium* plants could support and broaden their application as medicinal plants.

**FIGURE 1:**
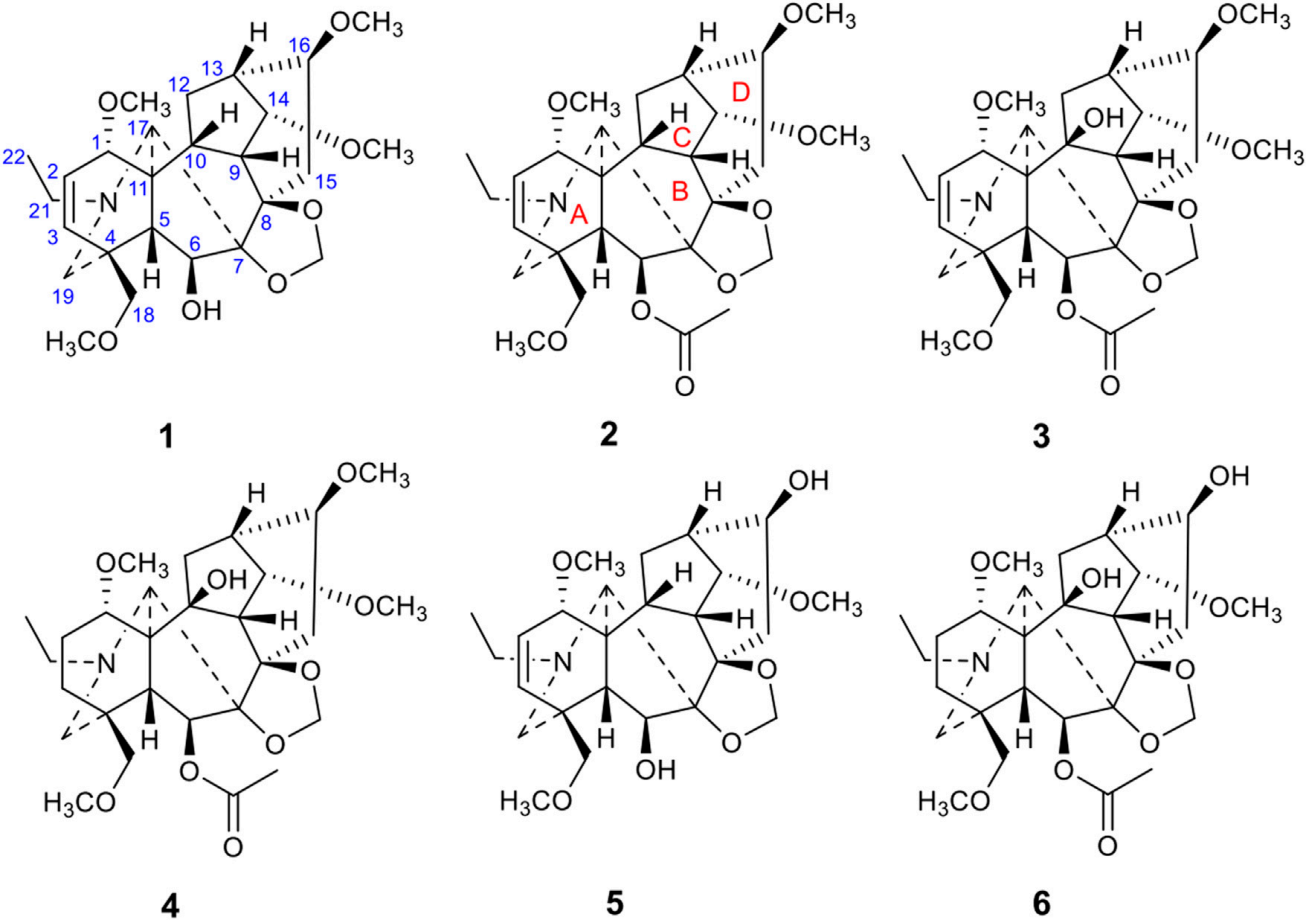
The chemical structures of six new diterpenoid alkaloids.

*D. grandiflorum* L., one of the best-known *Delphinium* species, is widely distributed in China and other Asian countries, including Mongolia, Korea, and the Russian Far East (Siberia) (Liu et al., 2009; Chen et al., 2017; Xu et al., 2021). This plant has a particularly long history as an ornamental flower and has been cultivated as a horticultural plant in Beijing City of China for hundreds of years. In addition, its roots or whole herbs are used for treating traumatic injury, toothache, and asthma in traditional Chinese medicine (Li et al., 2019). In previous investigations, a number of DAs, mainly C_19_-lycoctonine-type DAs, have been reported in *D. grandiflorum* (Batbayar et al., 2003; Wang et al., 2021), and some of them possess unprecedented DA skeletons. For example, Chen et al. reported two novel DAs, grandiflodine A and B, from *D. grandiflorum*. The former compound showed a C_19_-lycaconitine-type DA skeleton with cleavage of N-C19 and C7-C17 bonds and linkage of the N-C7 bond, while the latter represents a rare hetisine-type C_20_-DA with a broken N–C7 bond (Chen et al., 2017). In addition, two DAs, grandiflonines A and B reported by (Xu et al., 2021), possess an undescribed C_20_-hetisine-type DA skeleton with an open E ring. These exciting discoveries highlight the significant chemical diversity of DAs in *D. grandiflorum* and promote further in-depth studies on them. Hence, as part of our ongoing research exploring bioactive DAs with novel structures from *Delphinium* plants (Yin et al., 2022), the whole herb of *D. grandiflorum* was phytochemically investigated to afford fourteen C_19_-lycaconitine-type DAs (**1**–**14**), including six undescribed DAs, grandiflolines A–F (**1**–**6**) (Figure 1). The structural elucidation was accomplished by detailed spectroscopic analyses, mainly including 1D and 2D NMR, HR-MS, and IR spectra. Moreover, new alkaloids **1**–**6** were estimated for their potential anti-inflammatory effects in LPS-activated RAW 264.7 macrophages. This paper describes the extraction and isolation, structural elucidation, and activity screening of these compounds.

## Materials and methods

### General experimental procedures

1D (^1^H, ^13^C, and DEPT) and 2D (HSQC, HMBC, ^1^H–^1^H COSY, and NOESY) NMR spectra were obtained on a Bruker AM-500 spectrometer (Bruker, Germany) in CDCl_3_ or CD_3_OD (Qingdao Tenglong, China), and TMS was used as an internal reference. IR spectra were scanned on a Nicolet Magna-IR 550 spectrometer (Thermo Nicolet, United States) with KBr pellets. Optical rotations were measured on a Jasco P-1020 digital polarimeter (Jasco, Japan). HR-ESI-MS spectra were obtained on an Agilent 6230 LC/TOF MS spectrometer (Agilent, United States). The prep-HPLC experiment was performed on an Agilent 1260 pump coupled with an analytical preparative ZORBAXSB column (21.2 × 500 m, 5 µm). Silica gel (300–400 mesh, Qingdao Haiyang, China) was used in column chromatography, and Dragendorff’s reagent was used in TLC analysis (GF_254_ TLC plates, Qingdao Haiyang, China).

### Plant material

Whole herbs of *D. grandiflorum* were gathered in Longhua County of China in December 2020. The voucher specimen (2020-dg-1) identified by Zhang Jun from Kunming GenPHYTech Co., Ltd. is stored at Zunyi Medical University, China.

### Extraction and isolation

The whole herbs of *D. grandiflorum* (20 kg) were air-dried, crushed, and then extracted with 95% ethanol three for 3 days at room temperature (three times). The extracted solutions were combined and evaporated under reduced pressure to obtain ethanol extracts (∼2 kg), which were completely dissolved in water (2 L) at 70°C, adjusted to pH 1 with HCl and extracted with EtOAc (3 L × 3). After adjusting the acidic aqueous solution to pH 10 with sodium hydroxide, it was extracted with CHCl_3_ to obtain the crude alkaloid (320 g).

The crude alkaloid was divided into five fractions (Fr.A–Fr.E) by silica gel CC eluted with a CHCl_3_-CH_3_OH gradient system (100:1 → 0:1). Fr.A (5.4 g) was separated by silica gel CC (CHCl_3_-CH_3_OH-NH_4_OH, 100:1:1) to yield two subfractions (Fr.A1 and Fr.A2). The former subfraction was purified on silica gel CC (CHCl_3_-CH_3_OH, 1:1) to afford compound **7** (3 mg), while the latter was purified on silica gel CC (CHCl_3_-CH_3_OH- NH_4_OH, 100:1:1) to afford compound **2** (168 mg). Fr.B (20.7 g) was further purified by semipreparative HPLC over a ZORBAXSB C_18_ column eluted with petroleum ether-acetone-diethylamine (4:1:1) to obtain compound **1** (7 mg). Further silica gel CC purification of Fr.C (25.3 g) was carried out by elution with CH_3_OH-H_2_O (30:70 → 80:20) to yield four subfractions (Fr.C1–Fr.C4). Subfraction Fr.C1 was separated on silica gel CC (CHCl_3_-CH_3_OH-NH_3_H_2_O, 30:1:1) to obtain compounds **5** (5 mg) and **10** (98 mg), while subfraction Fr.C2 was purified by semipreparative HPLC (CH_3_OH-H_2_O, 65:35, 0.5% NH_4_OH) to yield compound **3** (4 mg). Fr.C3 was subjected to silica gel CC (CHCl_3_-CH_3_OH-NH_4_OH, 100:1:1) to afford two subfractions (SFr.C3-1 and SFr.C3-2). Fr.C3-1 was further separated by silica gel CC (PE-acetone-NH_3_H_2_O, 2:1:1) to afford compounds **8** (6 mg) and **9** (15 mg), and Fr.C3-2 was purified by semipreparative HPLC (CH_3_OH-H_2_O, 70:30, 0.5% NH_4_OH) to afford compounds **6** (7 mg) and **11** (12 mg). Fr.C4 was separated on a silica gel CC (PE-acetone-diethylamine, 4:1:1) to give two subfractions (Fr.C4-1 and Fr.C4-2), which was purified by semipreparative HPLC (CH_3_OH-H_2_O, 70:30, 0.5% NH_4_OH) to yield compounds **4** (13 mg) and **13** (2 mg). Fr.D (12.9 g) was purified by silica gel CC (PE-acetone-NH_3_H_2_O, 3:1:1) and semipreparative HPLC (CH_3_OH-H_2_O, 70:30, 0.5% NH_4_OH) to afford compound **12** (28 mg). Fr.E (14.1 g) was separated by silica gel CC (CHCl_3_-CH_3_OH-NH_4_OH, 100:1:1 → 220:1:1) along with semipreparative HPLC (CH_3_OH-H_2_O, 70:30, 0.5% NH_4_OH) to afford compound **14** (43 mg).

Grandifloline A (**1**): white powder; IR (KBr, cm^−1^): 3520, 2917, 2751, 1745, 1675, 1278, 1057, 805; ${\left[\alpha \right]}_{D}^{22}$ +26.0 (c = 0.1, CH_3_OH). ^1^H and ^13^C NMR spectral data are shown in Tables 1, 2. HR-ESI-MS *m/z* 478.2799 [M+H]^+^ (calcd for C_26_H_39_NO_7_, 478.2805).

**TABLE 1:** ^13^C NMR (125 MHz) data of compounds **1**–**6** (compounds **1**–**4** in CDCl_3_ and compounds **5** and **6** in CD_3_OD).

Nos	1	2	3	4	5	6
1	80.5 d	80.1 d	76.0 d	77.1 d	81.5 d	78.4 d
2	125.4 d	125.8 d	126.4 d	26.6 t	126.1 d	27.2 t
3	134.6 d	134.6 d	134.2 d	31.5 t	135.6 d	32.5 t
4	39.7 s	40.1 s	39.9 s	37.9 s	40.7 s	39.1 s
5	50.9 d	50.0 d	45.3 d	45.7 d	53.4 d	46.4 d
6	79.5 d	78.4 d	78.8 d	78.7 d	80.0 d	80.0 d
7	92.3 s	91.5 s	91.2 s	91.8 s	93.7 s	93.8 s
8	85.1 s	84.4 s	82.4 s	81.6 s	85.1 s	82.1 s
9	40.3 d	40.0 d	50.7 d	50.5 d	40.5 d	48.8 d
10	47.3 d	47.5 d	84.2 s	84.0 s	49.0 d	83.8 s
11	50.7 s	50.8 s	56.4 s	55.9 s	51.6 s	56.5 s
12	27.9 t	28.1 t	38.5 t	39.2 t	28.5 t	39.3 t
13	38.1 d	38.7 d	38.7 d	38.4 d	43.8 d	42.0 d
14	83.5 d	83.7 d	82.0 d	81.7 d	85.2 d	83.6 d
15	33.7 t	34.4 t	35.2 t	34.9 t	38.5 t	37.8 t
16	82.2 d	82.2 d	81.6 d	81.5 d	73.6 d	73.1 d
17	61.3 d	62.1 d	61.1 d	64.0 d	63.4 d	65.4 d
18	75.9 t	75.9 t	75.9 t	78.4 t	77.2 t	79.6 t
19	54.3 t	53.7 t	53.7 t	53.5 t	55.0 t	54.7 t
21	49.0 t	49.0 t	48.8 t	50.6 t	50.2 t	51.5 t
22	13.1 q	13.1 q	13.0 q	14.0 q	13.2 q	14.1 q
OCH_2_O	93.2 t	93.8 t	94.4 t	94.1 t	94.3 t	94.7 t
OCH_3_-1	56.3 q	56.1 q	56.1 q	55.5 q	56.1 q	55.7 q
OCH_3_-14	58.0 q	57.8 q	57.9 q	57.9 q	58.0 q	58.2 q
OCH_3_-16	56.5 q	56.4 q	56.3 q	56.4 q		
OCH_3_-18	59.8 q	59.7 q	59.7 q	59.5 q	59.7 q	59.6 q
OAc-6		170.1 s	169.9 s	170.0 s		171.9 s
		21.8 q	21.8 q	21.8 q		21.6 q

**TABLE 2:** ^1^H NMR (500 MHz, *δ*_H_, Mult., *J* in Hz) data of compounds **1**–**6** (compounds **1**–**4** in CDCl_3_ and compounds **5** and **6** in CD_3_OD).

Nos	1	2	3	4	5	6
1	3.40 d (3.6)	3.42 d (3.5)	3.82 d (3.9)	3.48 m	3.45 d (3.5)	3.59 m
2	6.03 dd (9.9, 3.6)	6.05 dd (10.0, 3.5)	6.10 dd (9.8, 3.9)	a 2.10 m	5.97 dd (10.0, 3.5)	a 2.10 m
				b 2.18 m		b 2.20 m
3	5.95 d (9.9)	5.92 d (10.0)	5.91 d (9.8)	a 1.70 m	5.91 d (10.0)	a 1.69 m
				b 1.41 m		b 1.36 m
4	—	—	—	—	—	—
5	1.83 d (2.2)	1.87 d (2.4)	2.12 d (2.4)	1.83 d (2.4)	1.68 d (2.3)	2.05d (3.7)
6	4.28 brs	5.47 brs	5.51 brs	5.51 brs	4.24 brs	5.50 brs
7	—	—	—	—	—	—
8	—	—	—	—	—	—
9	3.67 t (4.3)	3.53 t (7.2)	3.31 d (5.1)	3.30 d (5.1)	3.81 t (5.1)	3.54 d (5.0)
10	2.23 m	2.23 m	—	—	2.21 m	—
11	—	—	—	—	—	—
12	a 2.21 m	a 2.25 m	a 2.82 d (14.9)	a 3.21 t (5.4)	a 2.16 m	a 2.74 dd (7.3, 8.0)
	b 1.93 m	b 1.91 m	b 1.84 d (14.9)	b 1.73 t (5.4)	b 1.91 m	b 1.75 dd (7.3, 8.0)
13	2.40 m	2.38 m	2.56 m	2.53 m	2.22 m	2.37 m
14	3.70 t (5.2)	3.71 t (5.0)	4.13 t (5.7)	4.12 t (4.5)	3.75 t (4.0)	4.24 t (6.0)
15	2.53 dd (14.4, 8.6)	2.50 dd (12.8, 6.8)	2.52 t (8.1)	2.49 t (7.4)	2.48 dd (15.0, 7.5)	2.51 dd (16.6, 9.1)
	1.88 dd (14.4, 8.6)	1.85 dd (12.8, 6.8)	1.88 t (8.1)	1.83 t (7.4)	1.81 dd (15.0, 7.5)	1.71 dd (16.6, 9.1)
16	3.26 t (8.4)	3.27 t (9.0)	3.22 t (9.2)	3.19 t (8.9)	3.67 t (8.2)	3.61 t (7.8)
17	3.13 s	3.18 s	3.14 s	3.11 s	3.17 s	3.26 s
18	a 3.31	3.30	a 3.32	a 3.21	a 3.26	a 3.16
	ABq (9.2)	ABq (8.2)	ABq (9.5)	ABq (9.2)	ABq (9.2)	ABq (9.2)
	b 3.23	3.16	b 3.21	b 3.06	b 3.28	b 3.06
	ABq (9.2)	ABq (8.2)	ABq (9.5)	ABq (9.2)	ABq (9.2)	ABq (9.2)
19	a 2.45	a 2.48	a 2.51	a 2.71	a 2.40	a 2.73
	ABq (11.2)	ABq (11.5)	ABq (8.5)	ABq (11.9)	ABq (11.6)	ABq (11.4)
	b 2.32	b 2.44	b 2.49	b 2.40	b 2.31	b 2.38
	ABq (11.2)	ABq (11.5)	ABq (8.5)	ABq (11.9)	ABq (11.6)	ABq (11.4)
21	a 2.90 m	a 2.97 m	a 2.97 m	a 2.72 m	a 2.89 m	a 2.70 m
	b 2.60 m	b 2.63 m	b 2.63 m	b 2.81 m	b 2.56 m	b 2.81 m
22	1.08 t (7.2)	1.07 t (7.1)	1.07 t (7.2)	1.06 t (7.2)	1.03 t (7.0)	1.05 t (7.2)
OCH_2_O	a 5.12 s	a 4.95 s	a 4.97 s	4.94 s	a 5.14 s	4.88 s
	b 5.08 s	b 4.94 s	4.96 s	4.92 s	b 5.01 s	4.86 s
OCH_3_-1	3.32 s	3.34 s	3.34 s	3.26 s	3.30 s	3.27 s
OCH_3_-14	3.43 s	3.44 s	3.45 s	3.44 s	3.43 s	3.47 s
OCH_3_-16	3.38 s	3.36 s	3.35 s	3.33 s		
OCH_3_-18	3.36 s	3.30 s	3.30 s	3.25 s	3.33 s	3.23 s
OAc-6		2.08 s	2.09 s	2.08 s		2.05 s

Grandifloline B (**2**): white powder; IR (KBr, cm^−1^): 2936, 2881, 1741, 1649, 1452, 1366, 1224, 853; ${\left[\alpha \right]}_{D}^{22}$ +29.8 (c = 0.1, CH_3_OH). ^1^H and ^13^C NMR spectral data are shown in Tables 1, 2. HR-ESI-MS *m/z* 520.2909 [M+H]^+^ (calcd for C_28_H_41_NO_8_, 520.2910).

Grandifloline C (**3**): white powder; IR (KBr, cm^−1^): 3450, 2933, 2888, 1739, 1674, 1229, 1088, 819; ${\left[\alpha \right]}_{D}^{22}$ +41.26 (c = 0.1, CH_3_OH). ^1^H and ^13^C NMR spectral data are shown in Tables 1, 2. HR-ESI-MS *m/z* 536.2855 [M+H]^+^ (calcd for C_28_H_41_NO_9_, 536.2859).

Grandifloline D (**4**): white powder; IR (KBr, cm^−1^): 3515, 2934, 2874, 1739, 1650, 1366, 1090, 852; ${\left[\alpha \right]}_{D}^{22}$ -16.92 (c = 0.1, CH_3_OH). ^1^H and ^13^C NMR spectral data are shown in Tables 1, 2. HR-ESI-MS *m/z* 538.3012 [M+H]^+^ (calcd for C_28_H_43_NO_9_, 538.3016).

Grandifloline E (**5**): white powder; IR (KBr, cm^−1^): 3437, 2964, 2824, 1740, 1647, 1397, 1078, 813; ${\left[\alpha \right]}_{D}^{22}$ +13.8 (c = 0.1, CH_3_OH). ^1^H and ^13^C NMR spectral data are shown in Tables 1, 2. HR-ESI-MS *m/z* 464.2643 [M+H]^+^ (calcd for C_25_H_37_NO_7_, 464.2648).

Grandifloline F (**6**): white amorphous powder; IR (KBr, cm^−1^): 3467, 2932, 2875, 1741, 1631, 1245, 1054, 852; ${\left[\alpha \right]}_{D}^{22}$ -37.48 (c = 0.1, CH_3_OH). ^1^H and ^13^C NMR spectral data are shown in Tables 1, 2. HR-ESI-MS *m/z* 524.2854 [M+H]^+^ (calcd for C_27_H_41_NO_9_, 524.2859).

### NO production in RAW264.7 macrophages

A previously reported method was adopted to evaluate the *in vitro* anti-inflammatory activities of the newly isolated alkaloids (Wang et al., 2019). RAW264.7 cells were plated into 96-well microplates, stimulated with 1 μg/ml LPS, and treated with the alkaloid under test. The nondrug group and L-NMMA-treated group were set as blank and positive controls, respectively. After the macrophages were cultivated overnight, the NO content in the medium and the absorbance of the solution were measured at 570 nm. To exclude the toxic effects of the compound on the cells, MTS was added to the remaining medium to detect cell survival. The inhibition rate on NO generation was calculated by the following equation: inhibition rate (%) = (OD_nondrug group_–OD_sample group_)/OD_nondrug group_ × 100%).

## Results and discussion

### Structural identification of new compounds

**FIGURE 2:**
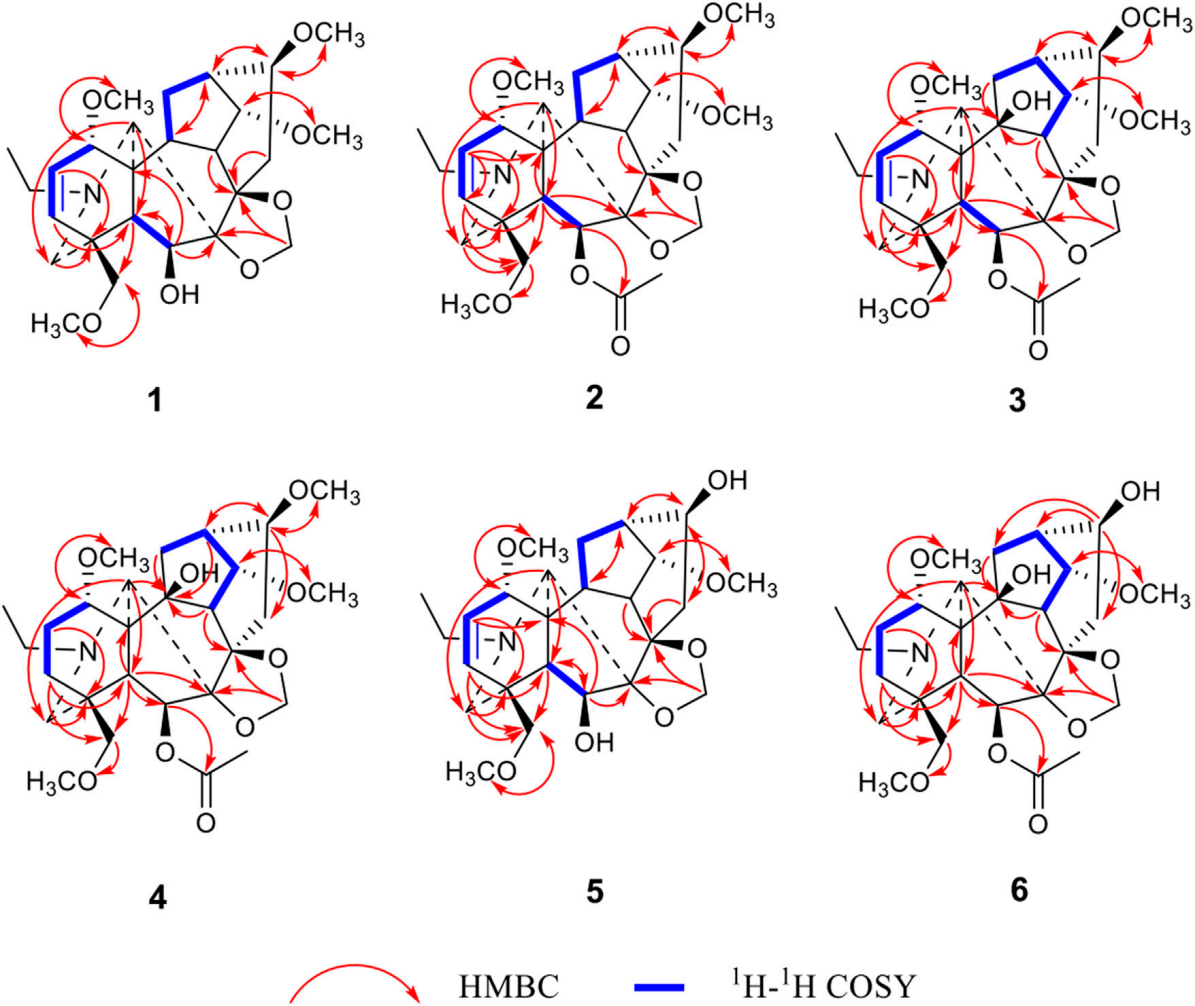
Key HMBC and ^1^H–^1^H COSY correlations of six new diterpenoid alkaloids.

**FIGURE 3:**
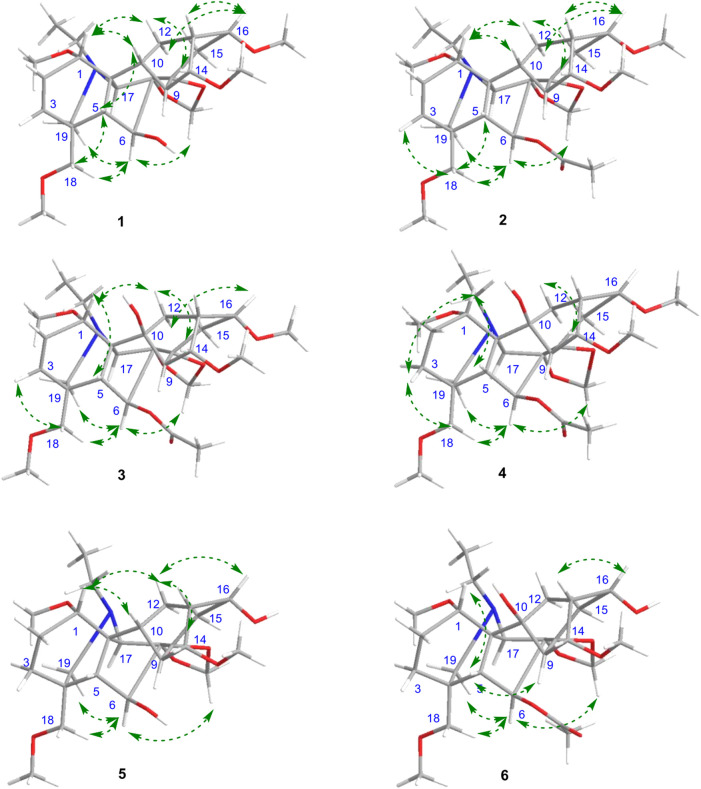
Key NOESY correlations of six new diterpenoid alkaloids.

Compound **1**, a white powder, exhibited a *pseudo* ion peak at *m*/*z* 478.2799 [M+H]^+^ in its HR-MS spectrum, which corresponded to the molecular formula C_26_H_39_NO_7_, with an unsaturation degree of eight. Its ^1^H NMR spectrum revealed the existence of an *N*CH_2_CH_3_ group (*δ*_H_ 1.08, t, *J* = 7.2 Hz, 3H), a characteristic *cis*-trisubstituted double bond (*δ*_H_ 6.03, dd, *J* = 9.9 Hz, 3.6 Hz; 5.95, d, *J* = 9.9 Hz; each 1H), four methoxyl (OCH_3_) groups (*δ*_H_ 3.32, 3.36, 3.38, 3.43, s, each 3H), and a methylenedioxy (OCH_2_O) group (*δ*_H_ 5.08, 5.12, s, each 1H). The ^13^C NMR spectrum suggested that compound **1** possesses 19 carbons in addition to the *N*CH_2_CH_3_, methoxy, and methylenedioxy groups, including four diagnostic quaternary carbons at *δ*_H_ 39.7 s, 50.7 s, 85.1 s, and 92.3 s (Yin et al., 2022). Combining the above data with biogenetic considerations implied that compound **1** could be a C_19_-lycoctonine-type DA (Meng et al., 2017). Seven oxygenated carbons were observed in the ^13^C NMR spectrum (*δ*_C_ 75.9 t, 79.5 d, 80.5 d, 82.2 d, 83.5 d, 85.1 s, 92.3 s), in addition to the molecular formula, suggesting an extra OH group in addition to the OCH_3_ and OCH_2_O groups. According to the HMBC correlations from OCH_3_-1 (*δ*_H_ 3.32, s) to C-1 (*δ*_C_ 80.5 d), OCH_3_-14 (*δ*_H_ 3.43 s) to C-14 (*δ*_C_ 83.5 d), OCH_3_-16 (*δ*_H_ 3.38 s) to C-16 (*δ*_C_ 82.2 d), and OCH_3_-18 (*δ*_H_ 3.36 s) to C-18 (*δ*_C_ 75.9 t), four OCH_3_ groups were located at C-1, C-14, C-16, and C-18 (Figure 2). The OCH_2_O group was placed at C-7 and C-8 as revealed by the long-range correlations from the methylene protons to C-7 and C-8. In addition, on the basis of the HMBC correlation networks from H-6 (*δ*_H_ 4.28, brs) to C-5 (*δ*_C_ 50.9 d), C-7 (*δ*_C_ 92.3 s), and C-11 (*δ*_C_ 50.7 s), the hydroxyl group was determined to be connected to C-6 (*δ*_C_ 79.5). The double bound was located at C-2 and C-3 based on the ^1^H–^1^H COSY correlation between H-1 and H-2, which was further confirmed by the HMBC correlations from H-2 to C-1 and C-11 and from H-3 to C-4, C-5, and C-18. Finally, the relative configuration of **1** was deduced from the NOESY experiment (Figure 3). The *α*-orientation of OCH_3_-1 was confirmed by the NOESY correlation between H-1*β* and H-10*β*, and between H-1*β* and H-12*β*. The *β*-orientation of OH-6 was deduced from the NOESY correlation between H-1*β* and H-10*β*, and between H-1*β* and H-12*β*. The orientations of the remaining oxygenated substituents are identical for all lycoctonine-type DAs, namely, the *α*-orientation of OCH_3_-14 and the *β*-orientation of OCH_3_-16, OCH_3_-18, and OCH_2_O (Ablajan et al., 2018). Hence, the structure of **1** was established with the assigned NMR data in Tables 1, 2.

Compound **2** was afforded as a white powder, and its molecular formula was determined to be C_28_H_41_NO_8_ by HR-MS at *m*/*z* 520.2909 [M+H]^+^. A characteristic absorption spectrum for ester carbonyl groups (1741 cm^−1^) was observed in the IR spectrum. The ^1^H NMR spectrum revealed the presence of an *N*CH_2_CH_3_ group (*δ*_H_ 1.03 t, *J* = 7.0 Hz, 3H), four OCH_3_ groups (*δ*_H_ 3.30, 3.34, 3.36, 3.44, s, each 3 H), a *cis*-trisubstituted double bond (*δ*_H_ 5.92, d, *J* = 10.0 Hz; 6.05, dd, *J* = 10.0 Hz, 3.4 Hz; each 1H), an OCH_2_O group (*δ*_H_ 4.94, 4.95, s, each 1H) and an OAc functional group (*δ*_H_ 2.08, s, 3H). The above NMR features indicated a C_19_-lycoctonine-type DA for **2** (Lin et al., 2017). Comparison of NMR data between compounds **2** and **1** (Tables 1, 2) revealed identical substituent patterns. Exceptionally, alkaloid **2** possesses an extra OAc group, which was placed at C-6 according to the HMBC correlation from H-6 to the ester carbonyl carbon (Figure 2). This could be further supported by the fact that H-6 in **2** was significantly shifted downfield from *δ*_H_ 4.28 in compound **1** to *δ*_H_ 5.47 due to the substituted effect (OH→OAc). Therefore, the structure of **2** was determined, and its relative configuration was consistent with that of **1**, as revealed by the NOESY experiment (Figure 3). All of the NMR data of **2** were assigned by 2D NMR and are listed in Tables 1, 2.

Compound **3** is a white powder, whose molecular formula was identified as C_28_H_41_NO_9_ by HR-MS at *m*/*z* 536.2855 [M+H]^+^. The IR spectrum indicated the existence of hydroxyl (3450 cm^−1^) and ester (1739 cm^−1^) groups**.** Compound **3** displayed characteristic NMR features for a C_19_-lycoctonine-type DA bearing an *N*CH_2_CH_3_ group (*δ*_H_ 1.07, t, *J* = 7.2 Hz, 3H), an OCH_2_O group (*δ*_H_ 4.96, 4.97, s, each 1H), four OCH_3_ groups (*δ*_H_ 3.30, 3.34, 3.35, 3.45, s, 3H), a *cis*-trisubstituted double bond (*δ*_H_ 6.10, dd, *J* = 9.8 Hz, 3.9 Hz; 5.91, d, *J* = 9.8 Hz; s, each 1H), and an OAc group (*δ*_H_ 2.09 s, 3H) (Ping et al., 2007). The NMR data of **3** were highly similar to those of **2** except that the chemical shift of C-10 in compound **3** was shielded downfield with approximately Δ*δ*_C_ 36. In addition, eight oxygenated carbons (*δ*_C_ 75.9 t, 76.0 d, 78.8 d, 81.6 d, 82.0 d, 82.4 s, 84.2 s, 91.2 s) were detected in the ^13^C NMR spectrum, in combination with the molecular formula, indicating the presence of an extra hydroxyl group in addition to the abovementioned substituents. The hydroxyl group should be positioned at C-10 due to the chemical shift of C-10, which could be further supported by the HMBC correlation networks from H-9, H-12, and H-17 to C-10 (Figure 2). The relative configuration of **3** was also deduced from the NOESY experiment (Figure 3), which was identical to those of compounds **1** and **2** (Meng et al., 2017). Thus, the structure of compound **3** was determined.

Compound **4** was afforded as a white powder. According to the protonated molecular ion at *m*/*z* 538.3012 [M+H]^+^ in the HR-MS spectrum, its molecular formula was identified as C_28_H_43_NO_9_. Analysis of its IR spectrum revealed hydroxyl (3515 cm^−1^) and ester (1739 cm^−1^) groups in the structure. In the ^1^H NMR spectrum, an *N*CH_2_CH_3_ group (*δ*_H_ 1.06, t, *J* = 7.4 Hz, 3 H), an OCH_2_O group (*δ*_H_ 4.92, 4.94, s, each 1H), four OCH_3_ groups (*δ*_H_ 3.25, 3.26, 3.33, 3.44, each 3H), and an OAc group (*δ*_H_ 2.08 s, 3H) were recognized. Apart from the above groups, compound **4** contains 19 carbons, including five diagnostic quaternary carbons (*δ*_C_ 37.9, 55.9, 81.6, 84.0, 91.8, s). These data, in combination with biogenetic consideration, suggested a C_19_-lycoctonine-type DA for **4** (Li et al., 2010). Moreover, a combined analysis of ^1^H and ^13^C NMR data suggested that **4** and **3** have similar structures. Correlations between OCH_3_-1 (*δ*_H_ 3.26, s) and C-1 (*δ*_C_ 77.1 d), OCH_3_-14 (*δ*_H_ 3.44, s) and C-14 (*δ*_C_ 81.7 d), OCH_3_-16 (*δ*_H_ 3.33, s) and C-16 (*δ*_C_ 81.5 d), OCH_3_-18 (*δ*_H_ 3.25, s) and C-18 (*δ*_C_ 78.4 d), and H-6 (*δ*_H_ 5.51, brs) with C=O (*δ*_C_ 170.0 s) in the HMBC spectrum confirmed the assignment of four OCH_3_ groups and the ester group. A hydroxyl group was placed at C-10 on the basis of the HMBC correlations from H-9 (*δ*_H_ 3.30, d), H-12 and H-13 (*δ*_H_ 2.53, m) to C-10 (*δ*_C_ 83.9) (Figure 2). Based on the long-range correlations of the OCH_2_O group with C-7 and C-8, this substituent group was located at C-7 and C-8. Furthermore, in a comparison between **4** and **3** in terms of their molecular formulas and NMR data, one of the most important differences was found to be the lack of the △^2,3^ in **4**. Therefore, its structure was determined and further confirmed by analysing its 2D NMR spectra (Figure 2). Similarly, the orientations for several oxygenated substituents at flexible positions, including OCH_3_-1*α* and OAc-6*β*, were determined by using the NOESY experiment (Figure 3) (Meng et al., 2017). Thus, the structural elucidation of compound **4** was accomplished.

Compound **5** is a white powder, and its molecular formula was determined to be C_25_H_37_NO_7_ by HR-MS at *m*/*z* 464.2643 [M+H]^+^. The ^1^H NMR spectrum showed an *N*CH_2_CH_3_ group (*δ*_H_ 1.03, t, *J* = 7.0 Hz, 3H), an OCH_2_O group (*δ*_H_ 5.01, 5.14, s, each 1H), three OCH_3_ groups (*δ*_H_ 3.30, 3.33, 3.43, s, each 3H), and a *cis*-trisubstituted double bond (*δ*_H_ 5.97, dd, *J* = 10.0 Hz, 3.8 Hz; 5.91, d, *J* = 10.0 Hz; each 1H). There are nineteen carbons in the ^13^C NMR spectrum of **5** other than the abovementioned groups, including four characteristic quaternary carbons (*δ*c 40.7, 51.6, 85.2, 93.7). Combining the data presented above with biogenetic considerations suggested that **5** might be a C_19_-lycoctonine-type DA (Zhang et al., 2016). In addition, seven oxygenated carbons (*δ*_C_ 73.6 d, 77.2 t, 80.0 d, 81.5 d, 85.1 s, 85.2 d, 93.7 s) were found in the ^13^C NMR spectrum, which corresponded to the molecular formula, implied two extra OH groups in **5**. On the basis of the HMBC correlation networks from H-6 (*δ*_H_ 4.24, brs) to C-5 (*δ*_C_ 53.4 d), C-7 (*δ*_C_ 93.7 s), and C-11 (*δ*_C_ 51.6 s) and from H-16 (*δ*_H_ 3.67, t) to C-13 (*δ*_C_ 43.8 d) and C-15 (*δ*_C_ 38.5 t), these two hydroxyl groups were determined to connect to C-6 (*δ*_C_ 80.0) and C-16 (*δ*_C_ 73.6), respectively. The double bond was located at C-2 and C-3 based on the ^1^H–^1^H COSY correlation between H-1 and H-2, which was further supported by the HMBC correlations from H-2 to C-1 and C-11 and from H-3 to C-4, C-5, and C-18. Three OCH_3_ groups were placed at C-1, C-14, and C-18 on the basis of the HMBC correlations from OCH_3_-1 (*δ*_H_ 3.30, s) to C-1 (*δ*c 81.5 d), OCH_3_-14 (*δ*_H_ 3.43 s) to C-14 (*δ*c 85.2 d), and OCH_3_-18 (*δ*_H_ 3.36 s) to C-18 (*δ*c 59.7 t), respectively. In addition, OCH_2_O was located at C-7 and C-8 due to the long-range correlations of the methylene group with C-7 (*δ*c 91.3 q) and C-8 (*δ*c 84.2 q) (Figure 2). The relative configurations of OCH_3_-1*α* and OH-6*β* were determined on the basis of the NOESY correlations between H-6 and H-18 and between H-1 and H-10, respectively (Liu et al., 2009). Thus, the structure of compound **5** was determined.

Compound **6** has the molecular formula C_27_H_41_NO_9_ as determined by the HR-MS experiment (*m*/*z* 524.2854 [M+H]^+^), suggesting eight degrees of unsaturation. Its IR spectrum showed the presence of hydroxyl (3467 cm^−1^) and ester (1741 cm^−1^) groups. The ^1^H NMR spectrum revealed the presence of an *N*CH_2_CH_3_ group (*δ*_H_ 1.05, t, *J* = 7.2 Hz, 3H), an OCH_2_O group (*δ*_H_ 4.86, 4.88, s, each 1H), an OAc group (*δ*_H_ 2.05, s, 3H; *δ*_C_ 21.6 q, 171.9 s), and three OCH_3_ groups (*δ*_H_ 3.23, 3.27, 3.47, s, each 3H). The ^13^C NMR spectrum showed 19 carbons in addition to the aforementioned groups, including four diagnostic quaternary carbons at *δ*_C_ 39.1, 56.5, 82.1, 93.8, thus revealing a C_19_-lycoctonine-type DA for **6** (Ablajan et al., 2018). Comparing all of the NMR data of **6** and **5**, it was found that the major difference was the lack of the △^2,3^ group for **6** and an additional ester group and oxygenated carbon (*δ*_C_ 83.8 s), which can be attributed to an OAc group and an OH group. Eight oxygenated carbons (*δ*_C_ 73.1 d, 78.4 d, 79.6 d, 80.0 d, 82.1 s, 83.6 d, 83.8 d, 93.8 s) presented in the ^13^C NMR spectrum also implied the presence of two OH groups along with three OCH_3_ groups, an OAc group, and an OCH_2_O group in **6** (Xu et al., 2021). The extra OH group could be positioned at C-10 due to the HMBC correlations from H-9 (*δ*_H_ 3.54, d) and H-12 (*δ*_H_ 1.75, d) to C-10 (*δ*_C_ 83.8 s) (Figure 2). Additionally, the HMBC correlation from H-6 (*δ*_H_ 5.50, s) to the carbonyl carbon suggested that the OAc group is connected at C-6. Thus, the structure of compound **6** was determined.

The remaining compounds were identified by comparison of the ^1^H and ^13^C NMR data with those in the literature. Finally, they were identified as eight known C_19_-lycaconitine-type DAs, namely, tatsiensine (**7**) (Wang et al., 2003), deacetyltatsiensine (**8**) (Pelletier et al., 1983), siwanines A and B (**9** and **10**) (Zhang and Ou, 1998), elasine (**11**) (Pelletier et al., 1989), anthriscifoldine B (**12**) (Song et al., 2009), browniine (**13**) (Zhou et al., 2005), and lycoctonine (**14**) (Zeng et al., 2007). Among them, compounds **7**–**12** have not been found in *D. grandiflorum* in previous studies.

### Biological activity

**FIGURE 4:**
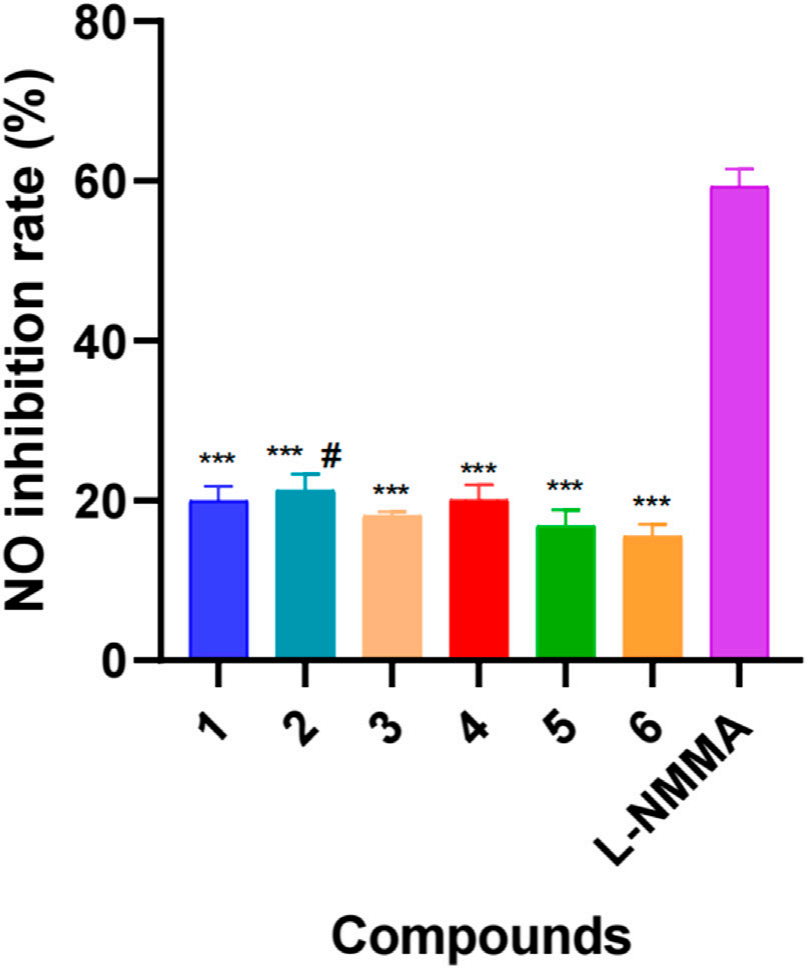
Inhibitory effects of six new new diterpenoid alkaloids on NO production in LPS-induced RAW264.7 cells. L-NMMA: L-NMMA was used as positive control. Error bars indicate SD, #, *p <* 0.05 vs. compound **6**, ***, *p <* 0.001 vs. L-NMMA.

Traditionally, *Delphinium* plants have been extensively utilized to treat arthritis and other inflammatory diseases, which implies that their major constituents, namely, diterpenoid alkaloids, possess certain anti-inflammatory activity. It has been reported in previous studies that a certain number of diterpenoid alkaloids, mainly aconitine-type and lycoctonine-type alkaloids, have exhibited *in vitro* anti-inflammatory activity (Shen et al., 2020; Qasem et al., 2022). For example, two typical lycoctonine-type DAs, delbrunine and eldeline, from *D. brunonianum* Royle showed good anti-inflammatory activity in LPS-activated RAW 264.7 cells, which could significantly restrain the elevation of inflammatory factors, including NO, TNF-α (tumor necrosis factor-α), IL-6 (interleukin-6), COX-2 (cyclooxygenase 2), and iNOS (inducible nitric oxide synthase) through NF-κB signaling pathway (Wang et al., 2010). Another analogous *Delphinium* alkaloid A from *D. giraldii* Diels suppressed the overexpression of the proinflammatory factors TNF-α, IL-6 and IL-8 significantly in LPS-infected Caco2 cells (Liu et al., 2019). Our previous studies also indicated that three aconitine-type DAs, taronenines A, B and D, showed inhibitory effects on the production of IL-6 in LPS-activated RAW 264.7 cells, which exerted IC_50_ values of 29.6, 18.8, and 25.4 μg/ml, respectively (Yin et al., 2018). Accordingly, in the present study, all of the new compounds were screened for their inhibitory activities against NO in LPS-activated RAW 264.7 cells. As a result, all of the isolated new alkaloids exhibited a weak inhibitory effect, exerting an inhibition rate of approximately 20% at a concentration of 50 µM (Figure 4). This might be attributed to the lack of key pharmacophores responsible for their anti-inflammatory activity, such as aromatic ester groups at C-14 or C-8 (Tang et al., 2022).

## Conclusion

DAs have attracted increasing interest due to their complex and diverse structures and bioactivities. In the present study, six previously undescribed C_19_-lycoctonine-type DAs were isolated and identified from the whole plant of *D. grandiflorum*. New alkaloids **1**–**3** and **5** possess a characteristic △^2,3^ functional group in the A ring, while compounds 5 and 6 feature a rare OH-16 substituent. In addition, known compounds **7**–**12** were isolated from *D. grandiflorum* for the first time. The results of our work enriched the chemical diversity of *D. grandiflorum* and the genus *Delphinium* and presented beneficial information for further investigations. Compounds **1**–**6** only exhibit weak inhibition activities of NO in LPS-activated RAW 264.7 macrophages. The results suggested that the anti-inflammatory effect of *D. grandiflorum* might be attributed to the other DA compounds. Thus, further studies are still needed to elucidate the material basis of *D. grandiflorum* in inflammation-mediated diseases.

## Data Availability

The original contributions presented in the study are included in the article/Supplementary Material, further inquiries can be directed to the corresponding author.
