# Supplementary material for: Grandiflolines A–F, new anti-inflammatory diterpenoid alkaloids isolated from Delphinium grandiflorum
Source: Front Chem. 2022 Sep 19;10:1012874. doi: 10.3389/fchem.2022.1012874 (PMC9527285; doi:10.3389/fchem.2022.1012874)
Supplement: Supplementary file 1 [file DataSheet1.pdf]

frontiers

Supplementary Material

Grandiflolines A–F, New Anti-Inflammatory Diterpenoid Alkaloids Isolated from *Delphinium grandiflorum*

Yuanfeng Yan, Hongjun Jiang, Xiaoyan Yang, Zongbao Ding, Tianpeng Yin*

Faculty of Bioengineering, Zhuhai Campus of Zunyi Medical University, Zhuhai, China

*** Correspondence:**
Tianpeng Yin
ytp@zmu.edu.cn

List of Supporting Information

content

Figure S1: HRESIMS spectrum of compound 1 4
Figure S2: IR spectrum of compound 1 5
Figure S3: ^{1}H-NMR (500 MHz, $CDCl_3$) spectrum of compound 1 6
Figure S4: ^{13}C-NMR and DEPT (125 MHz, $CDCl_3$) spectrum of compound 1 6
Figure S5: HSQC spectrum of compound 1 7
Figure S6: HMBC spectrum of compound 1 7
Figure S7: ^{1}H-^{1}H COSY spectrum of compound 1 8
Figure S8: NOESY spectrum of compound 1 8
Figure S9: HRESIMS spectrum of compound 2 9
Figure S10: IR spectrum of compound 2 10
Figure S11: ^{1}H-NMR (500 MHz, $CDCl_3$) spectrum of compound 2 11
Figure S12: ^{13}C-NMR and DEPT (125 MHz, $CDCl_3$) spectrum of compound 2 11
Figure S13: HSQC spectrum of compound 2 12
Figure S14: HMBC spectrum of compound 2 12
Figure S15: ^{1}H-^{1}H COSY spectrum of compound 2 13
Figure S16: NOESY spectrum of compound 2 13
Figure S17: HRESIMS spectrum of compound 3 14
Figure S18: IR spectrum of compound 3 15
Figure S19: ^{1}H-NMR (500 MHz, $CDCl_3$) spectrum of compound 3 16
Figure S20: ^{13}C-NMR and DEPT (125 MHz, $CDCl_3$) spectrum of compound 3 16
Figure S21: HSQC spectrum of compound 3 17
Figure S22: HMBC spectrum of compound 3 17
Figure S23: ^{1}H-^{1}H COSY spectrum of compound 3 18
Figure S24: NOESY spectrum of compound 3 18
Figure S25: HRESIMS spectrum of compound 4 19
Figure S26: HRESIMS spectrum of compound 4 20
Figure S27: ^{1}H-NMR (500 MHz, $CDCl_3$) spectrum of compound 4 21
Figure S28: ^{13}C-NMR and DEPT (125 MHz, $CDCl_3$) spectrum of compound 4 21
Figure S29: HSQC spectrum of compound 4 22
Figure S30: HMBC spectrum of compound 4 22
Figure S31: ^{1}H-^{1}H COSY spectrum of compound 4 23
Figure S32: NOESY spectrum of compound 4 23
Figure S33: HRESIMS spectrum of compound 5 24
Figure S34: IR spectrum of compound 5 25
Figure S35: The ^{1}H-NMR Spectrum ($CDCl_3$) of Compound 5 26
Figure S36: ^{13}C-NMR and DEPT (125 MHz, CD_3OD) spectrum of compound 5 26
Figure S37: HSQC spectrum of compound 5 27
Figure S38: HMBC spectrum of compound 5 27
Figure S39: ^{1}H-^{1}H COSY spectrum of compound 5 28
Figure S40: NOESY spectrum of compound 5 28
Figure S41: HRESIMS spectrum of compound 6 29
Figure S42: IR spectrum of compound 6 30
Figure S43: ^{1}H-NMR (500 MHz, CD_3OD) spectrum of compound 6 31
Figure S44: ^{13}C-NMR (125 MHz, CD_3OD) spectrum of compound 6 31
Figure S45: HSQC spectrum of compound 6 32

Figure S46: HMBC spectrum of compound 6 ...32
Figure S47: ^1H-^1H COSY spectrum of compound 6 ...33
Figure S48: NOESY spectrum of compound 6 ...33
Figure S49: ^1H-NMR (500 MHz, $CDCl_3$) spectrum of compound 7 ...34
Figure S50: ^{13}C-NMR and DEPT (125 MHz, $CDCl_3$) spectrum of compound 7 ...34
Figure S51: ^1H-NMR (500 MHz, $CDCl_3$) spectrum of compound 8 ...35
Figure S52: ^{13}C-NMR and DEPT (125 MHz, $CDCl_3$) spectrum of compound 8 ...35
Figure S53: ^1H-NMR (500 MHz, $CDCl_3$) spectrum of compound 9 ...36
Figure S54: ^{13}C-NMR and DEPT (125 MHz, $CDCl_3$) spectrum of compound 9 ...36
Figure S55: ^1H-NMR (500 MHz, $CDCl_3$) spectrum of compound 10 ...37
Figure S56: ^{13}C-NMR and DEPT (125 MHz, $CDCl_3$) spectrum of compound 10 ...37
Figure S57: ^1H-NMR (500 MHz, $CDCl_3$) spectrum of compound 11 ...38
Figure S58: ^{13}C-NMR and DEPT (125 MHz, $CDCl_3$) spectrum of compound 11 ...38
Figure S59: ^1H-NMR (500 MHz, $CDCl_3$) spectrum of compound 12 ...39
Figure S60: ^{13}C-NMR and DEPT (125 MHz, $CDCl_3$) spectrum of compound 12 ...39
Figure S61: ^1H-NMR (500 MHz, $CDCl_3$) spectrum of compound 13 ...40
Figure S62: ^{13}C-NMR and DEPT (125 MHz, $CDCl_3$) spectrum of compound 13 ...40
Figure S63: ^1H-NMR (500 MHz, $CDCl_3$) spectrum of compound 14 ...41
Figure S64: ^{13}C-NMR and DEPT (125 MHz, $CDCl_3$) spectrum of compound 14 ...41

Formula Predictor Report - Ehr565.lcd

Page 1 of 1

Data File: E:\DATA\2021\0508\Ehr565.lcd

Elmt	Val.	Min	Max	Elmt	Val.	Min	Max	Elmt	Val.	Min	Max	Elmt	Val.	Min	Max	Use Adduct
H	1	10	100	F	1	0	0	S	2	0	0	Br	1	0	0	H
2H	1	0	0	Na	1	0	0	Cl	1	0	0	Pd	2	0	0	Na
C	4	5	60	Mg	2	0	0	Co	2	0	0	Ag	1	0	0	
N	3	0	15	Si	4	0	0	Cu	2	0	0	I	3	0	0	
O	2	0	30	P	3	0	0	Se	2	0	0					

Error Margin (ppm): 5
HC Ratio: unlimited
Max Isotopes: all
MSn Iso RI (%): 75.00

DBE Range: -2.0 - 100.0
Apply N Rule: no
Isotope RI (%): 1.00
MSn Logic Mode: OR

Electron Ions: both
Use MSn Info: yes
Isotope Res: 10000
Max Results: 20

Event#: 1 MS(E+) Ret. Time : 0.360 -> 0.387 Scan# : 55 -> 59

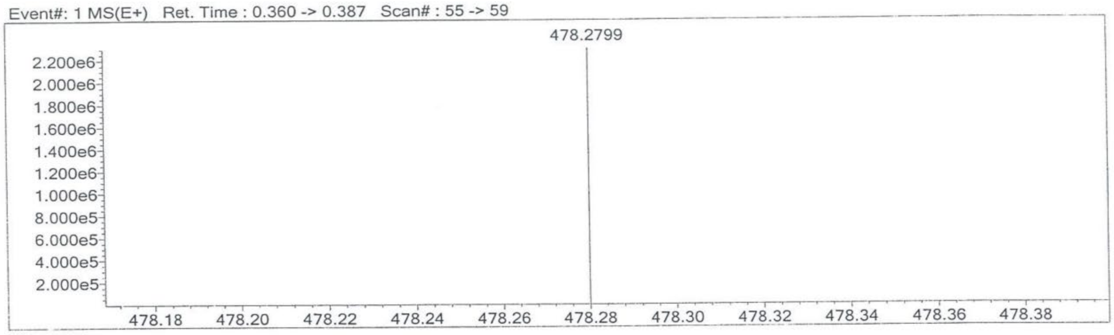

Measured region for 478.2799 m/z

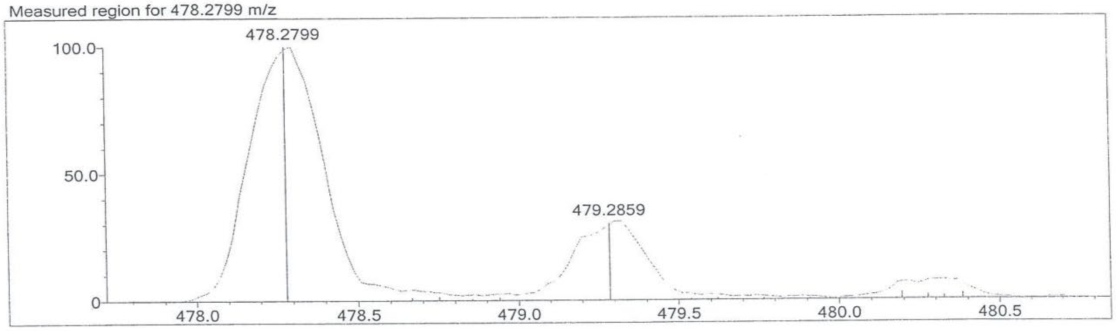

C26 H39 N O7 [M+H]+ : Predicted region for 478.2799 m/z

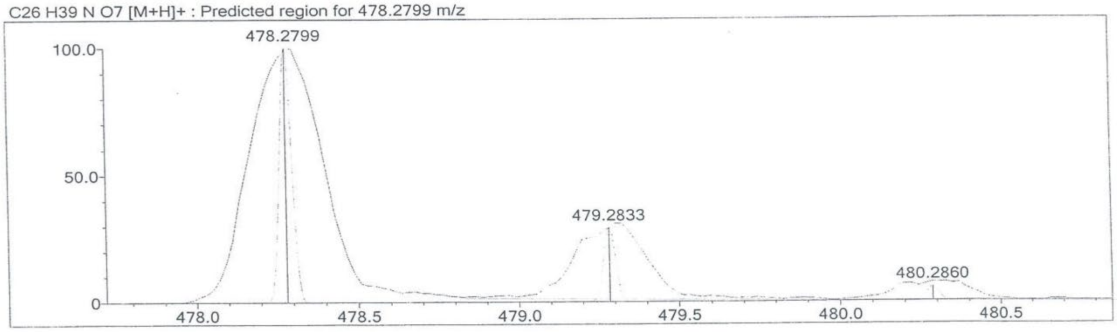

Formula (M)	Ion	Meas. m/z	Pred. m/z	Df. (mDa)	Df. (ppm)	DBE
C26 H39 N O7	[M+H]+	478.2799	478.2799	-0.0	0.00	8.0

Figure S1: HRESIMS spectrum of compound 1

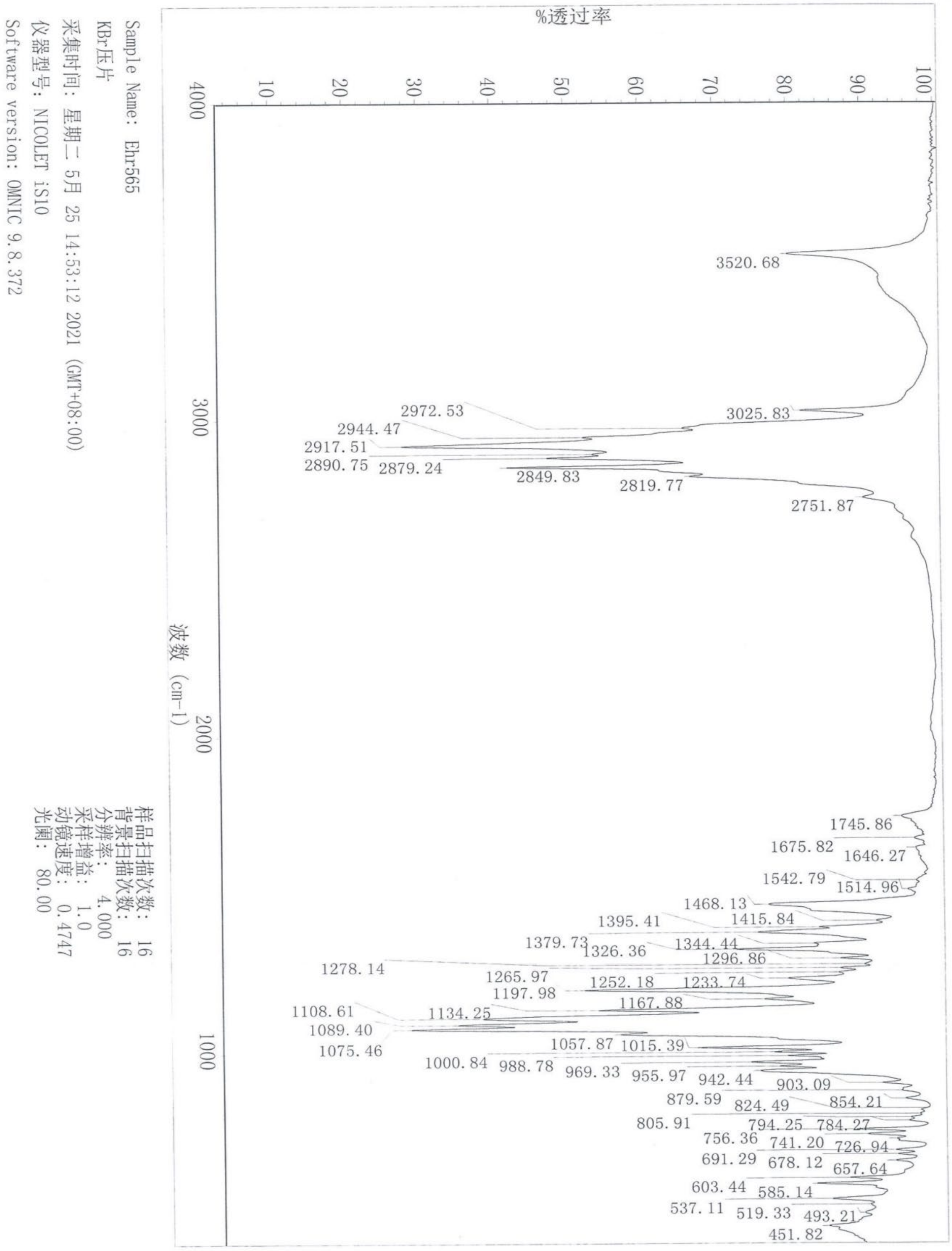

Figure S2: IR spectrum of compound 1

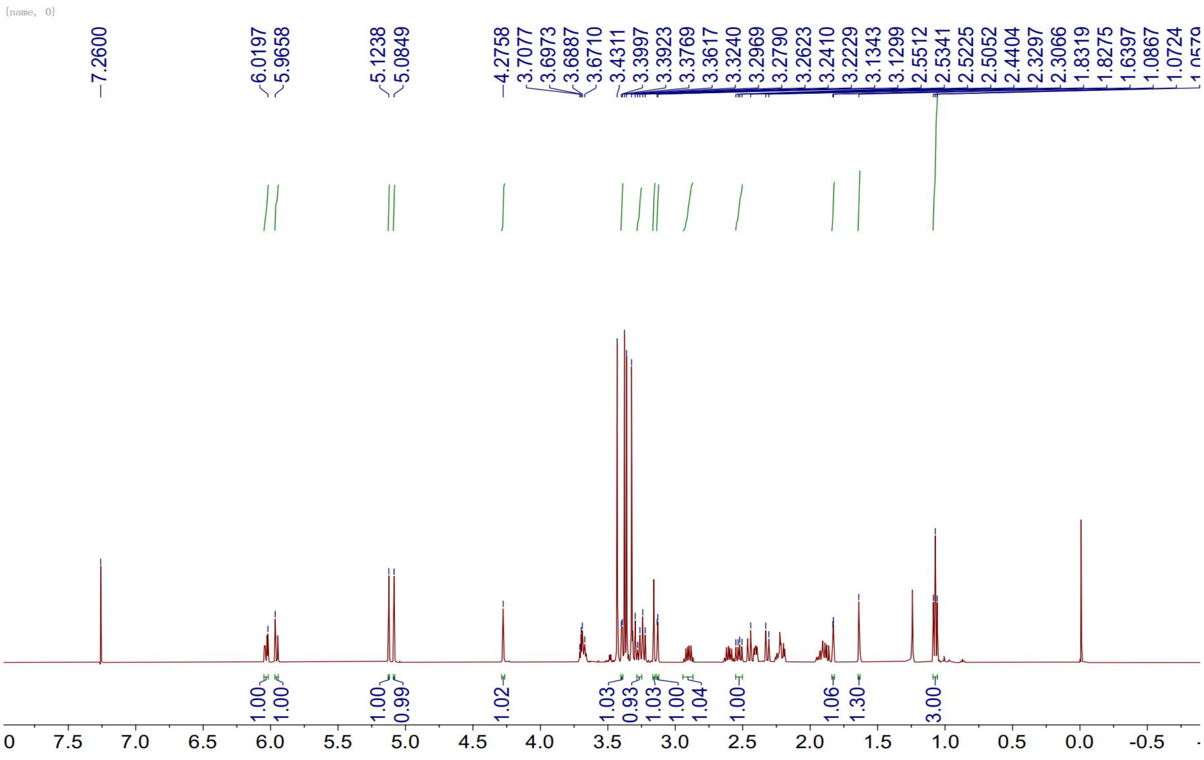

Figure S3: ^{1}H-NMR (500 MHz, $CDCl_3$) spectrum of compound 1

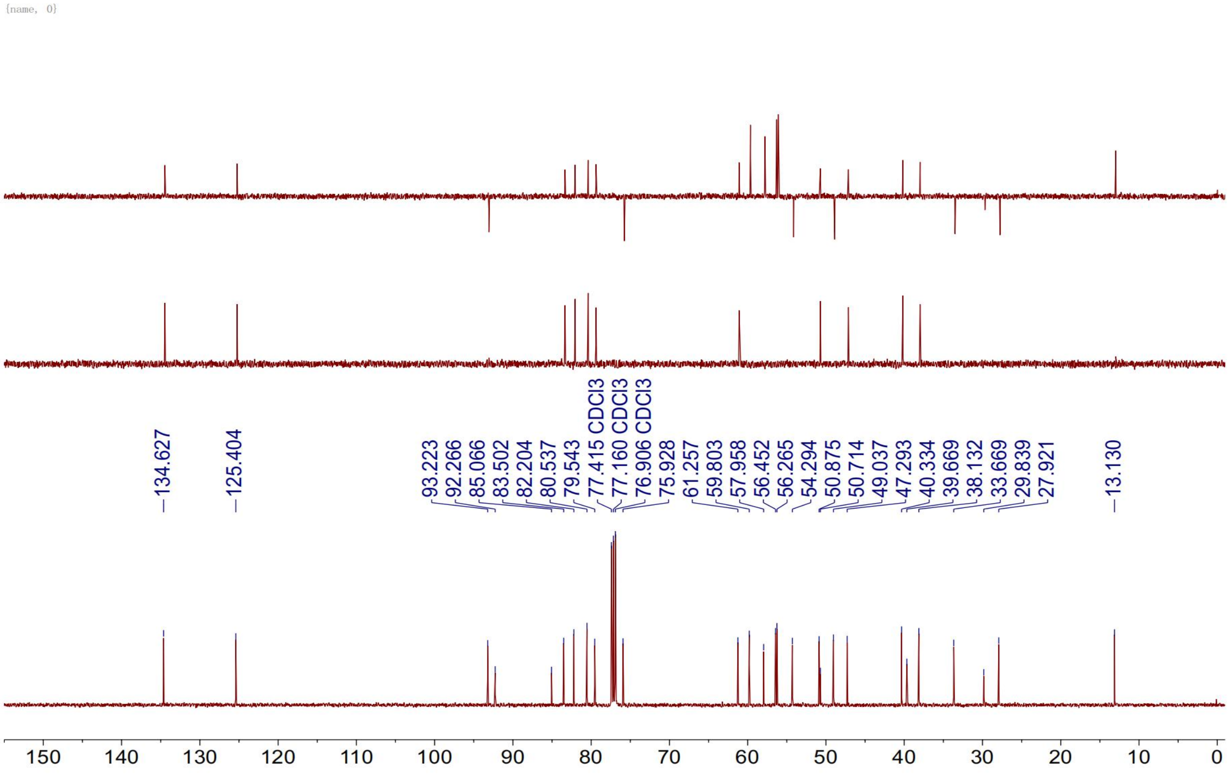

Figure S4: ^{13}C-NMR and DEPT (125 MHz, $CDCl_3$) spectrum of compound 1

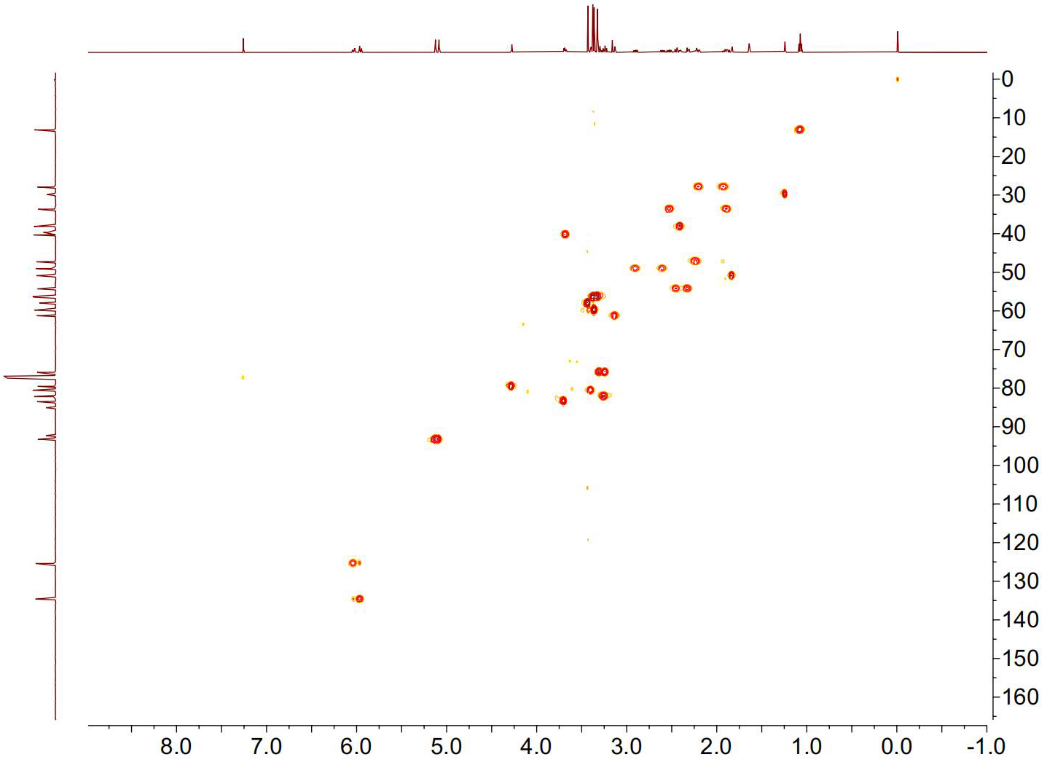

Figure S5: HSQC spectrum of compound 1

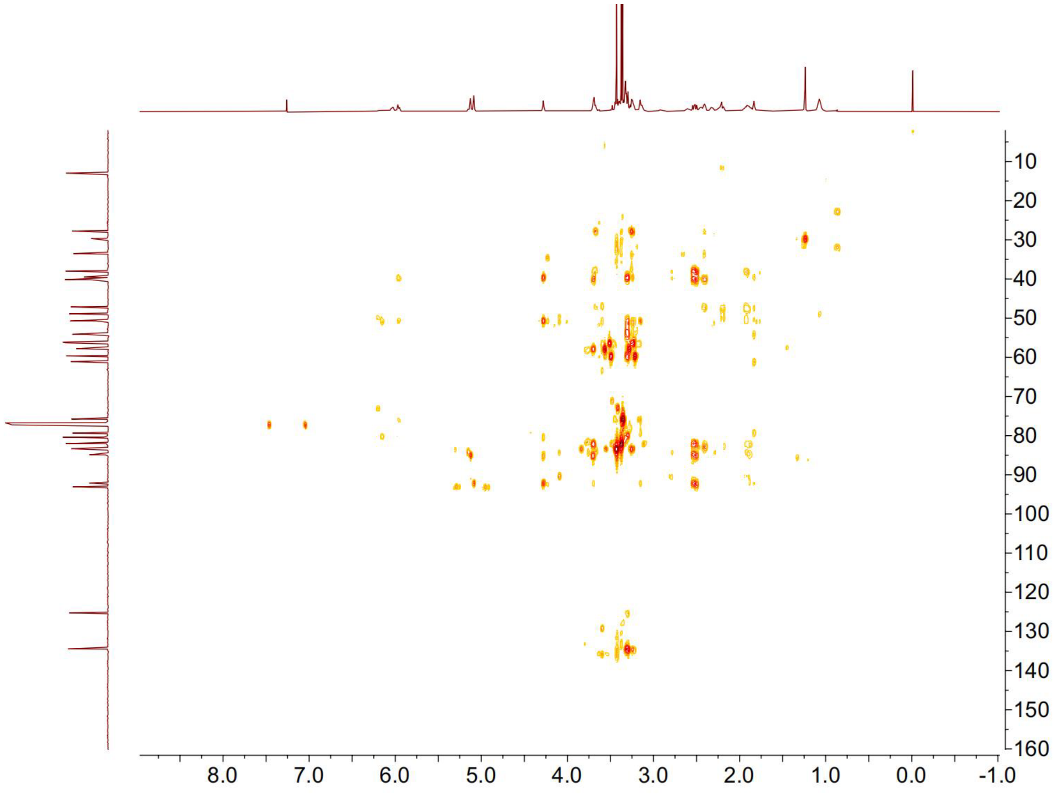

Figure S6: HMBC spectrum of compound 1

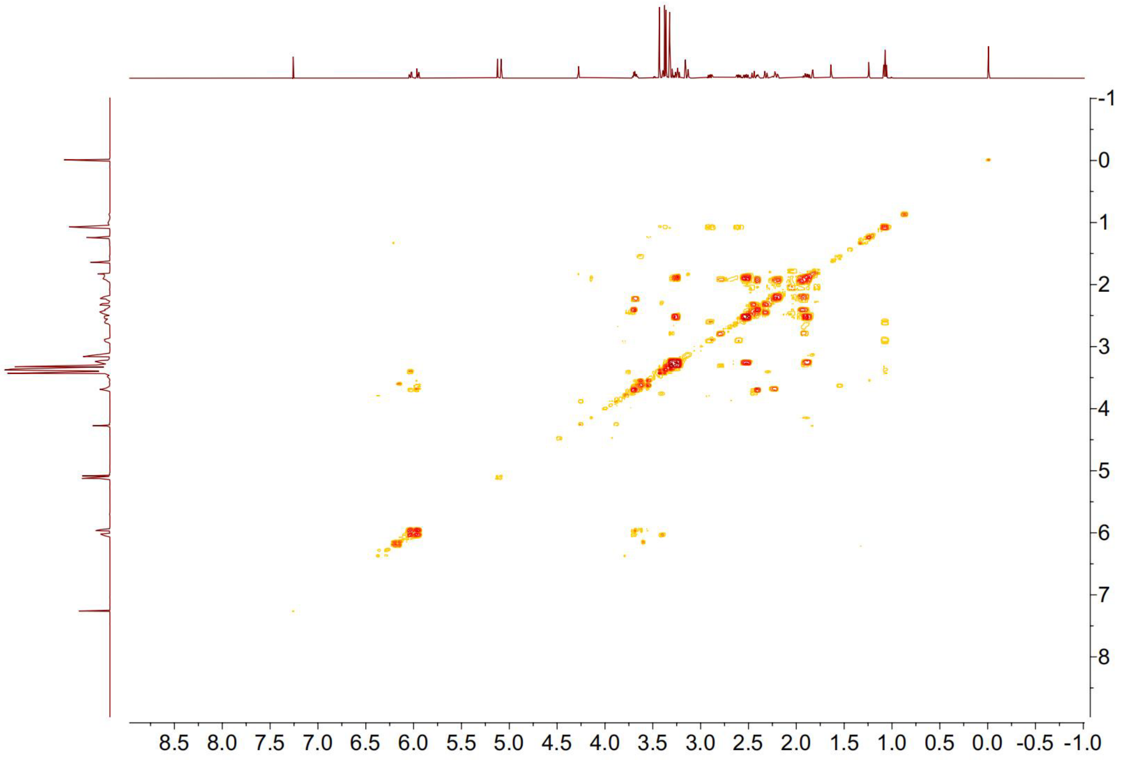

Figure S7: ^{1}H-^{1}H COSY spectrum of compound 1

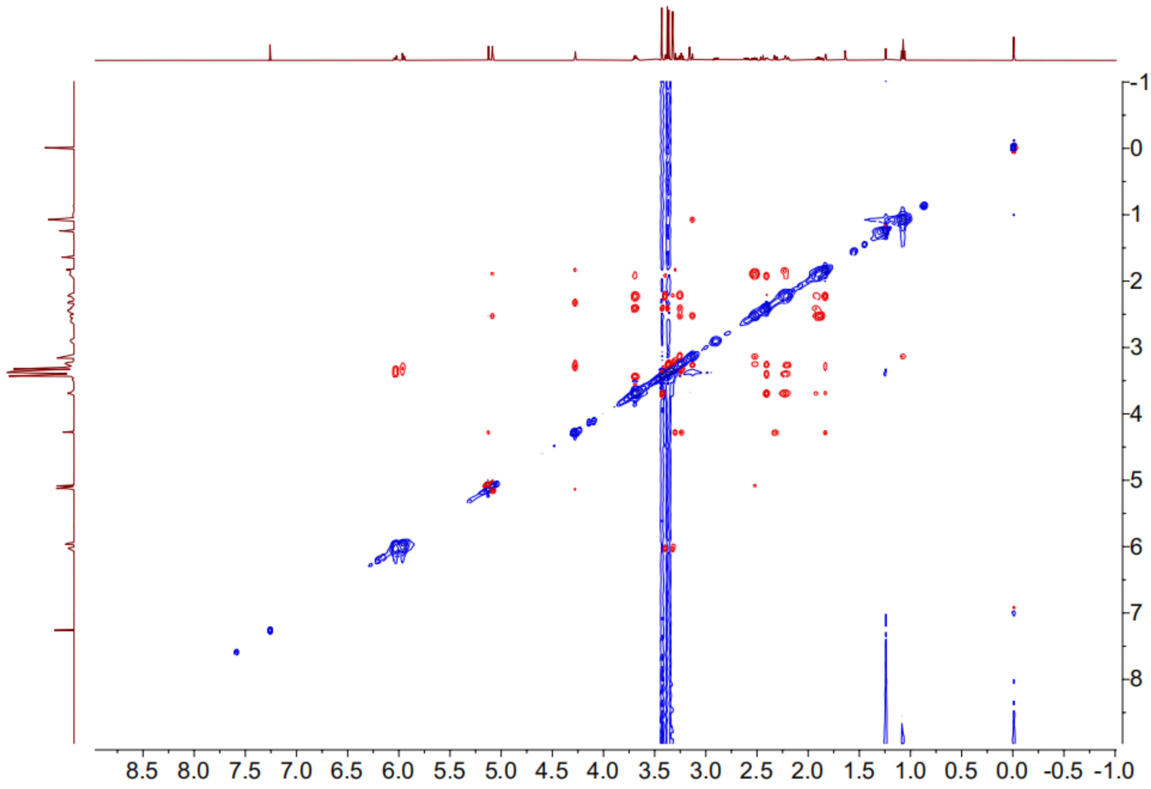

Figure S8: NOESY spectrum of compound 1

Data File: E:\DATA\2021\0325\Ehr555.lcd

Elmt	Val.	Min	Max	Elmt	Val.	Min	Max	Elmt	Val.	Min	Max	Elmt	Val.	Min	Max	Use Adduct
H	1	10	100	F	1	0	0	S	2	0	0	Br	1	0	0	H
2H	1	0	0	Na	1	0	0	Cl	1	0	0	Pd	2	0	0	Na
C	4	5	50	Mg	2	0	0	Co	2	0	0	Ag	1	0	0	
N	3	0	10	Si	4	0	0	Cu	2	0	0	I	3	0	0	
O	2	0	30	P	3	0	0	Se	2	0	0					

Error Margin (ppm): 5
HC Ratio: unlimited
Max Isotopes: all
MSn Iso RI (%): 75.00

DBE Range: -2.0 - 100.0
Apply N Rule: yes
Isotope RI (%): 1.00
MSn Logic Mode: OR

Electron Ions: both
Use MSn Info: yes
Isotope Res: 10000
Max Results: 20

Event#: 1 MS(E+) Ret. Time : 0.307 -> 1.000 Scan# : 47 -> 151

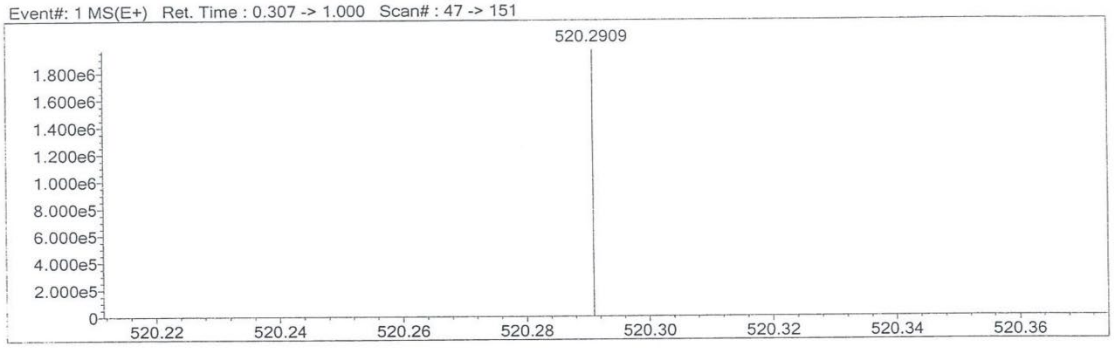

Measured region for 520.2909 m/z

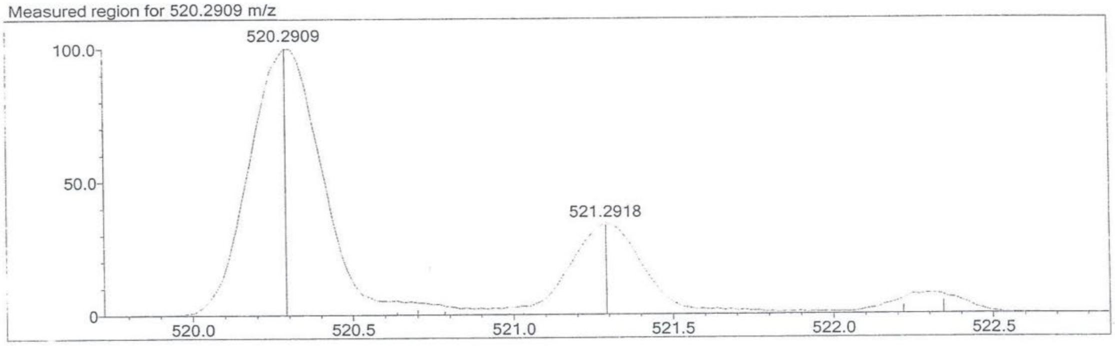

C28 H41 N O8 [M+H]+ : Predicted region for 520.2905 m/z

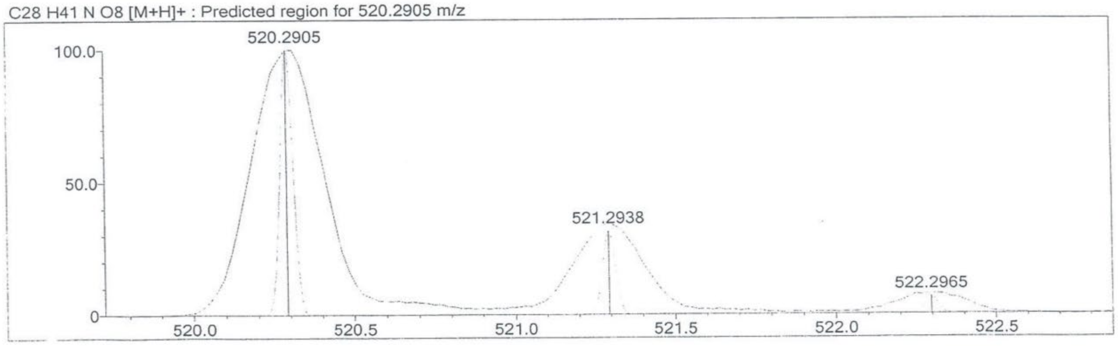

Formula (M)	Ion	Meas. m/z	Pred. m/z	Df. (mDa)	Df. (ppm)	DBE
C28 H41 N O8	[M+H]+	520.2909	520.2905	0.4	0.77	9.0

Figure S9: HRESIMS spectrum of compound 2

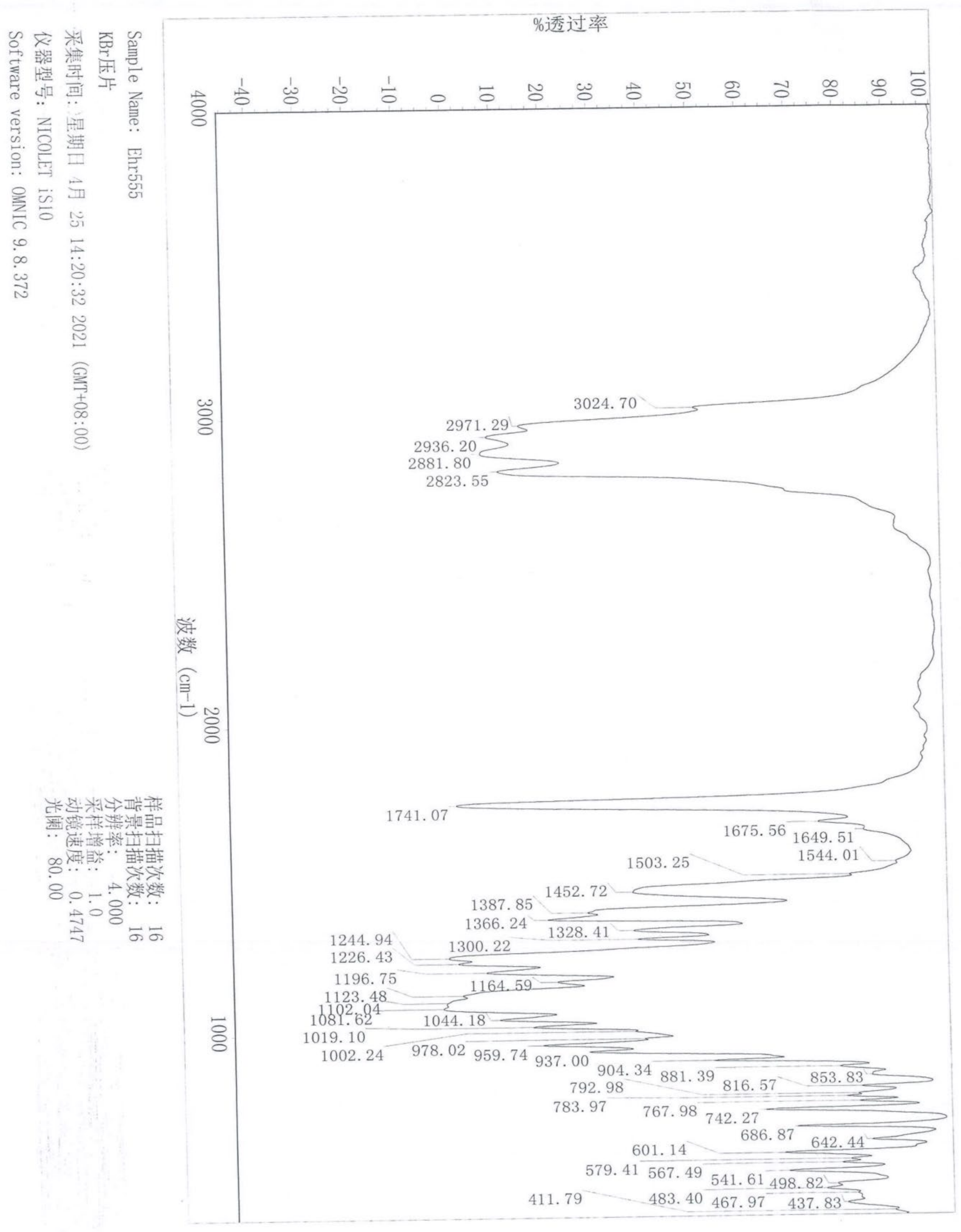

Figure S10: IR spectrum of compound 2

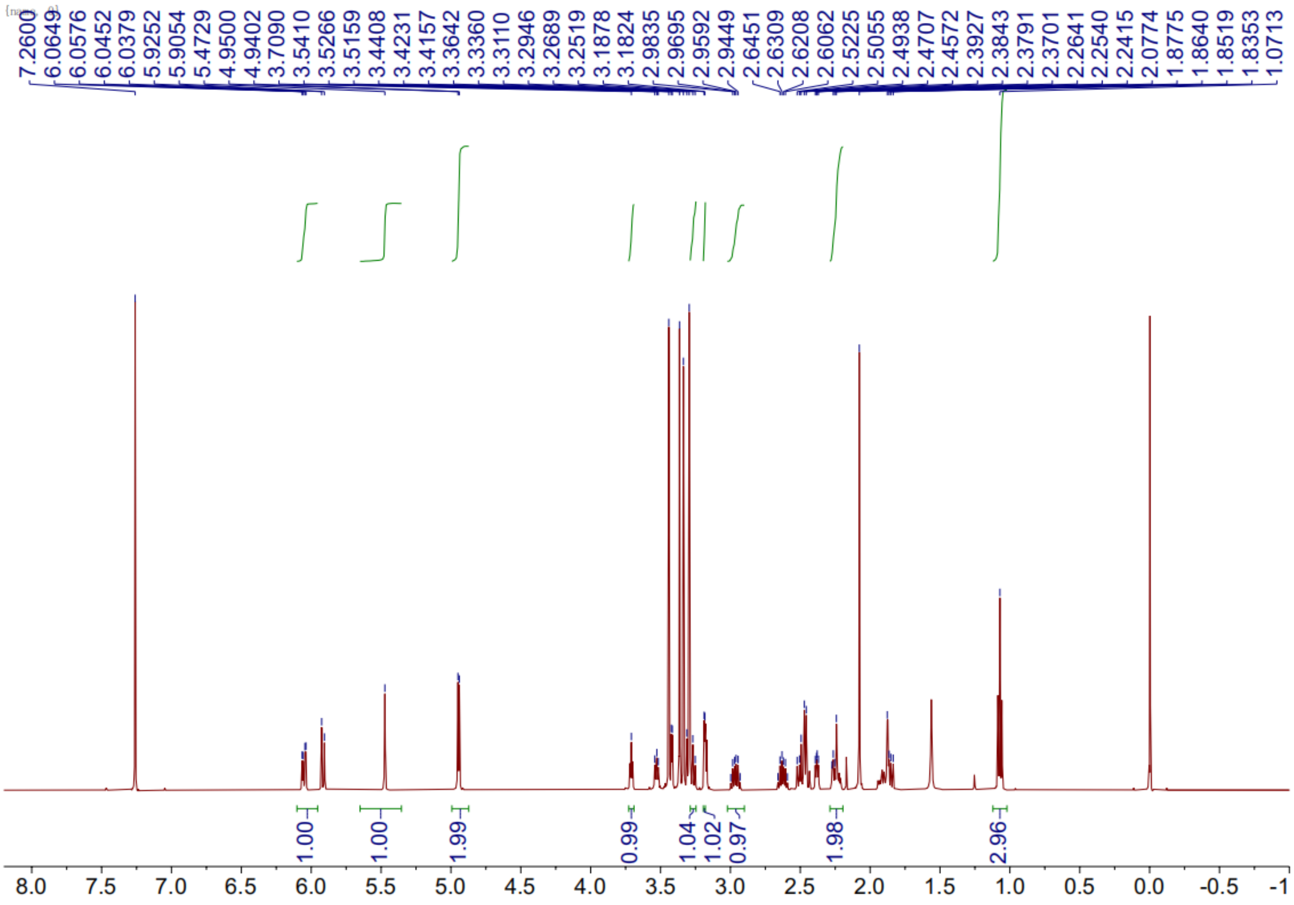

Figure S11: ^{1}H-NMR (500 MHz, $CDCl_3$) spectrum of compound 2

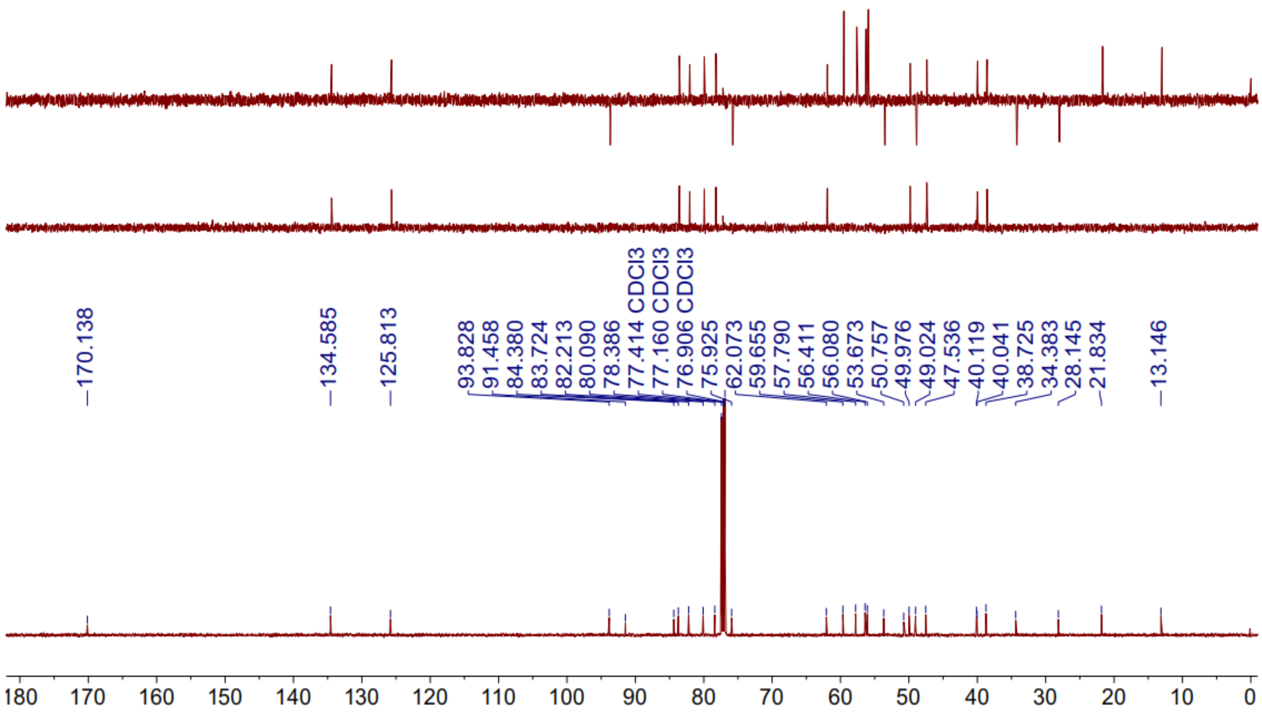

Figure S12: ^{13}C-NMR and DEPT (125 MHz, $CDCl_3$) spectrum of compound 2

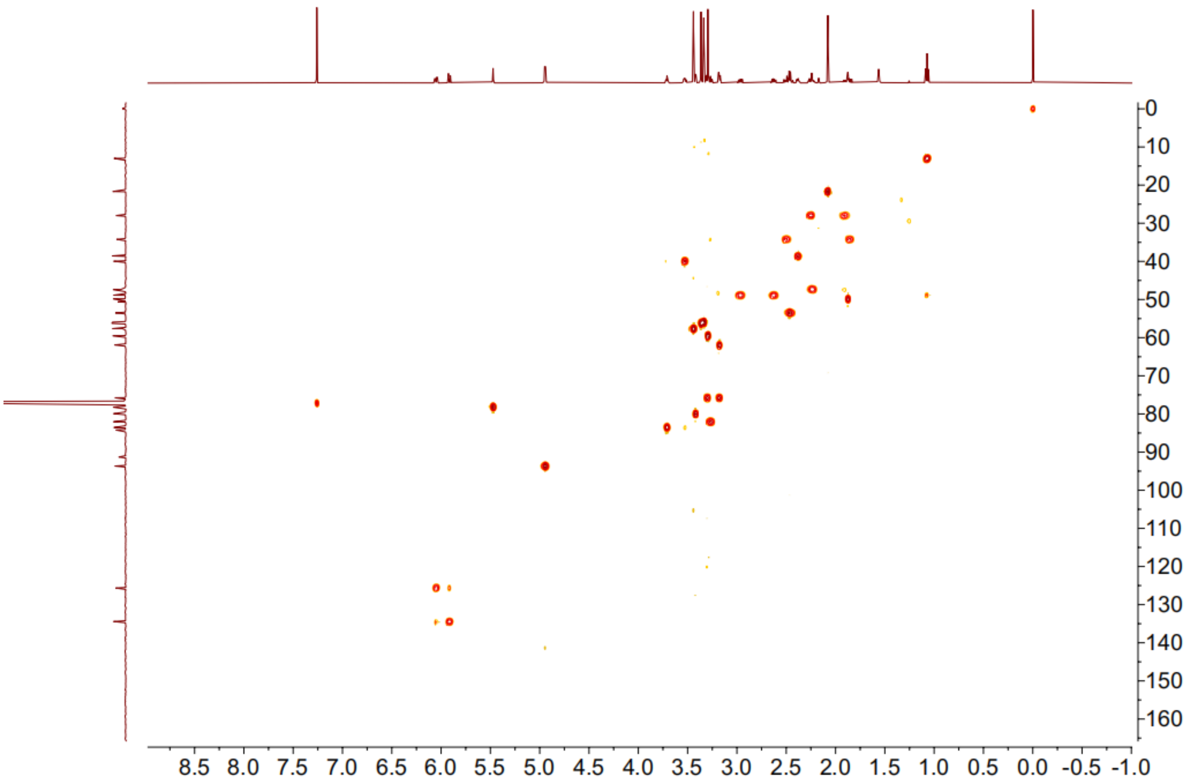

Figure S13: HSQC spectrum of compound 2

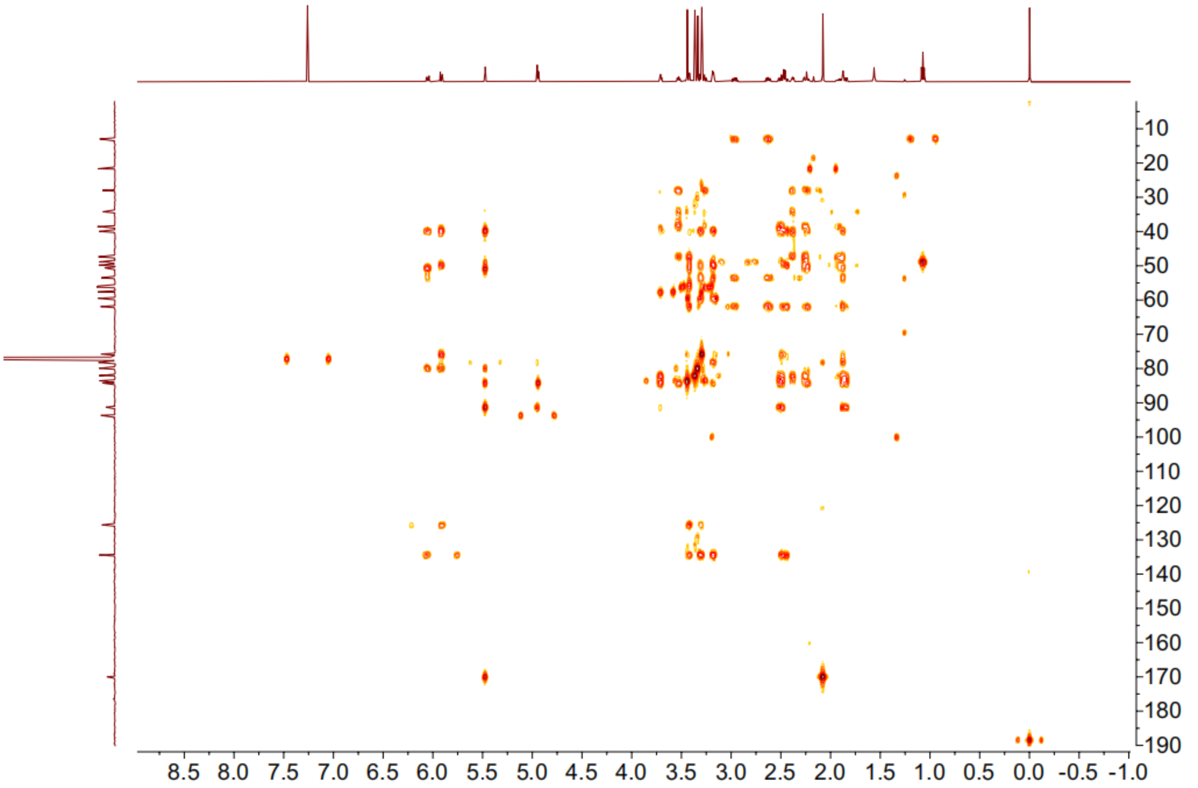

Figure S14: HMBC spectrum of compound 2

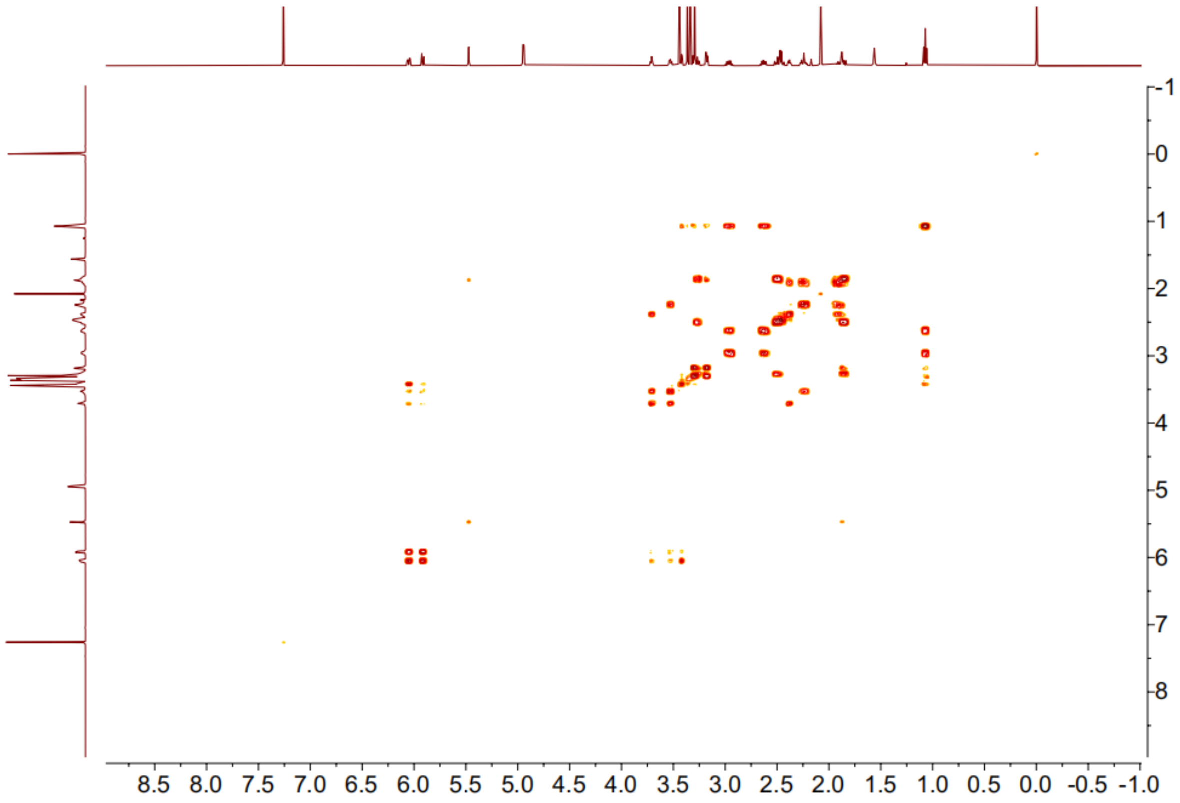

Figure S15: ^{1}H-^{1}H COSY spectrum of compound 2

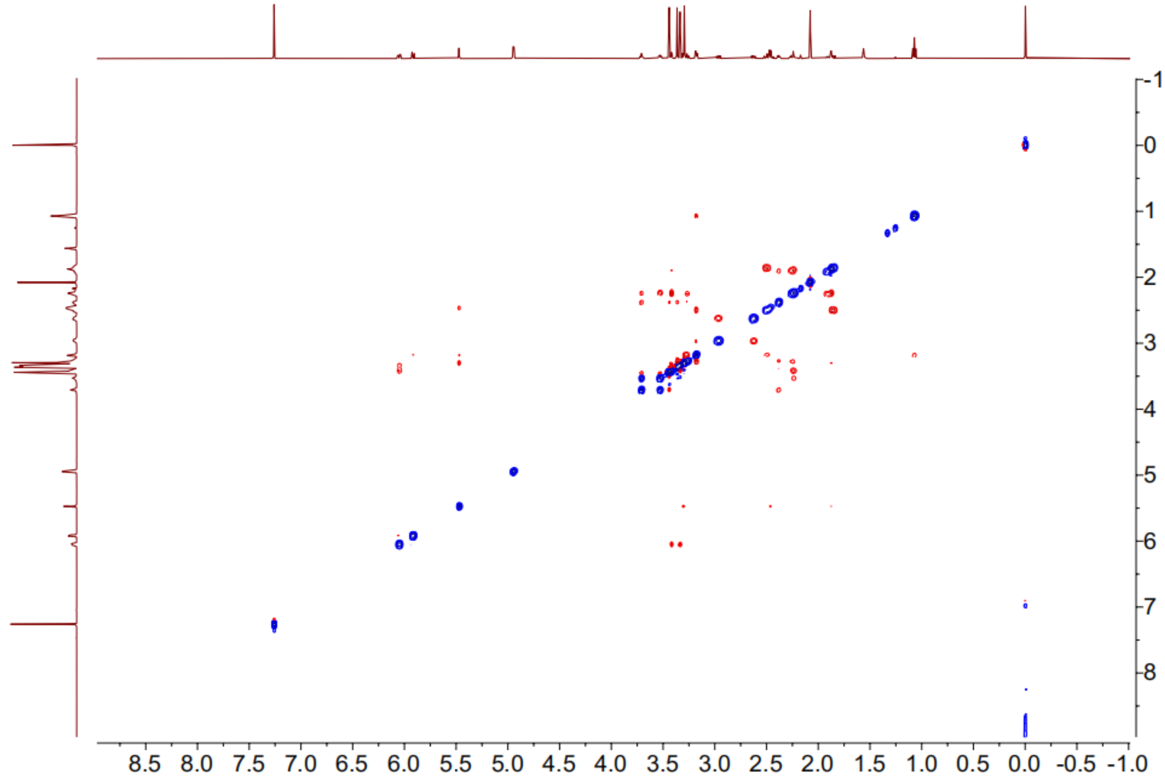

Figure S16: NOESY spectrum of compound 2

Formula Predictor Report - Ehr566.lcd

Data File: E:\DATA\2021\0519\Ehr566.lcd

Elmt	Val.	Min	Max	Elmt	Val.	Min	Max	Elmt	Val.	Min	Max	Elmt	Val.	Min	Max	Use Adduct
H	1	10	1000	F	1	0	0	S	2	0	0	Br	1	0	5	H
2H	1	0	0	Na	1	0	0	Cl	1	0	0	Pd	2	0	0	
C	4	5	50	Mg	2	0	0	Co	2	0	0	Ag	1	0	0	
N	3	0	15	Si	4	0	0	Cu	2	0	0	I	3	0	0	
O	2	0	30	P	3	0	0	Se	2	0	0					

Error Margin (ppm): 5
HC Ratio: unlimited
Max Isotopes: all
MSn Iso RI (%): 75.00

DBE Range: not fixed
Apply N Rule: no
Isotope RI (%): 1.00
MSn Logic Mode: OR

Electron Ions: both
Use MSn Info: yes
Isotope Res: 10000
Max Results: 20

Event#: 1 MS(E+) Ret. Time : 0.427 Scan# : 65

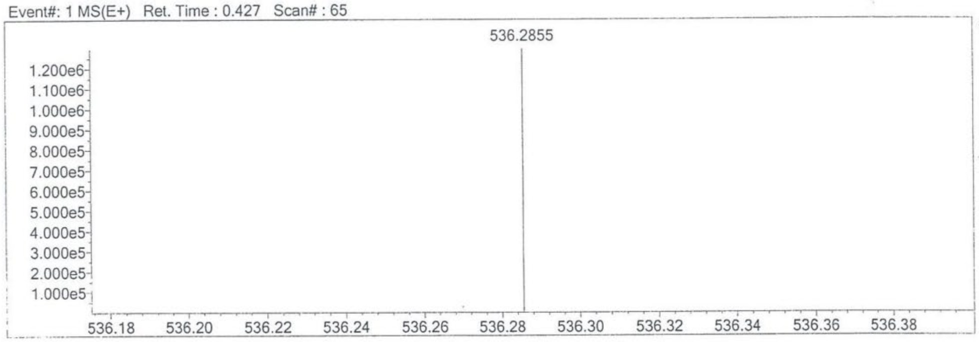

Measured region for 536.2855 m/z

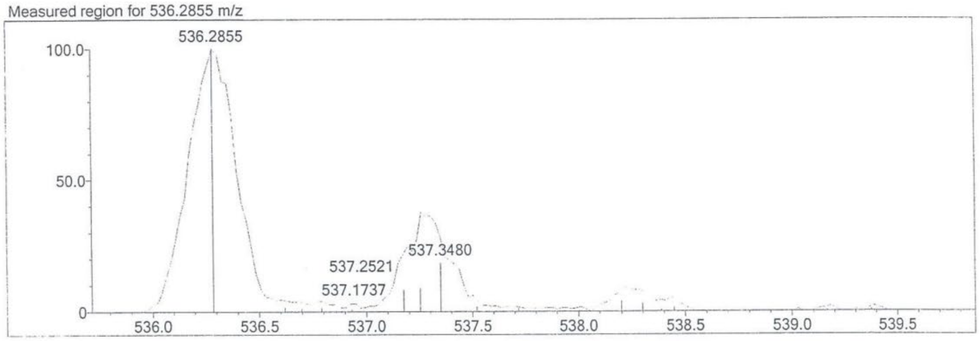

C28 H41 N O9 [M+H]+ : Predicted region for 536.2854 m/z

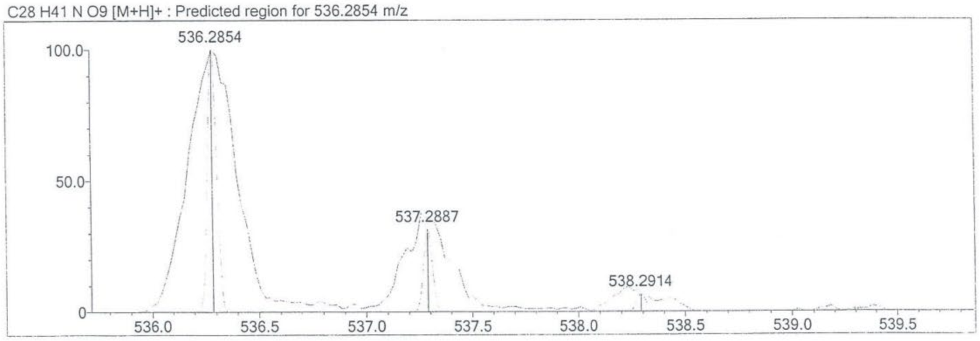

Formula (M)	Ion	Meas. m/z	Pred. m/z	Df. (mDa)	Df. (ppm)	DBE
C28 H41 N O9	[M+H]+	536.2855	536.2854	0.1	0.19	9.0

Figure S17: HRESIMS spectrum of compound 3

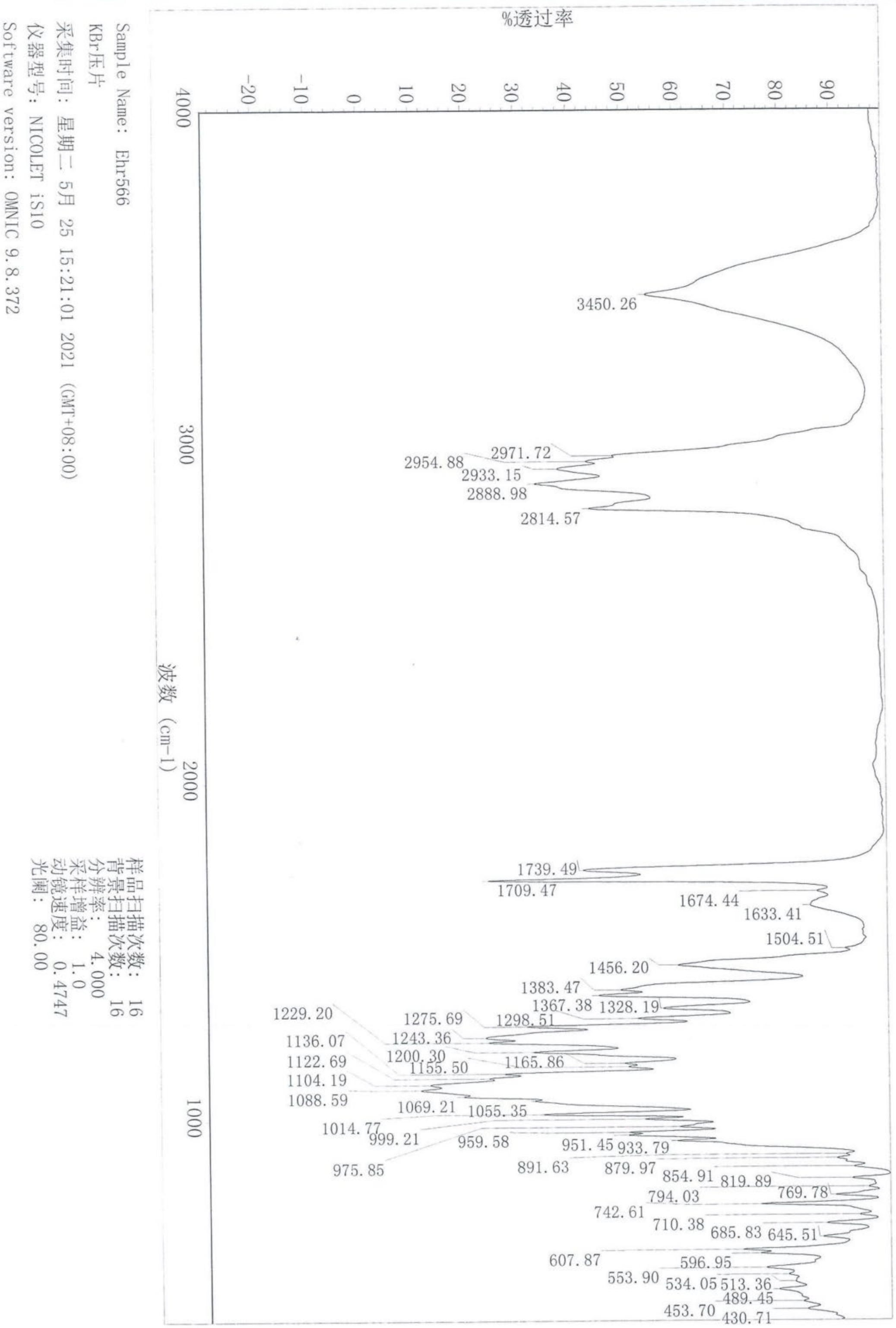

Figure S18: IR spectrum of compound 3

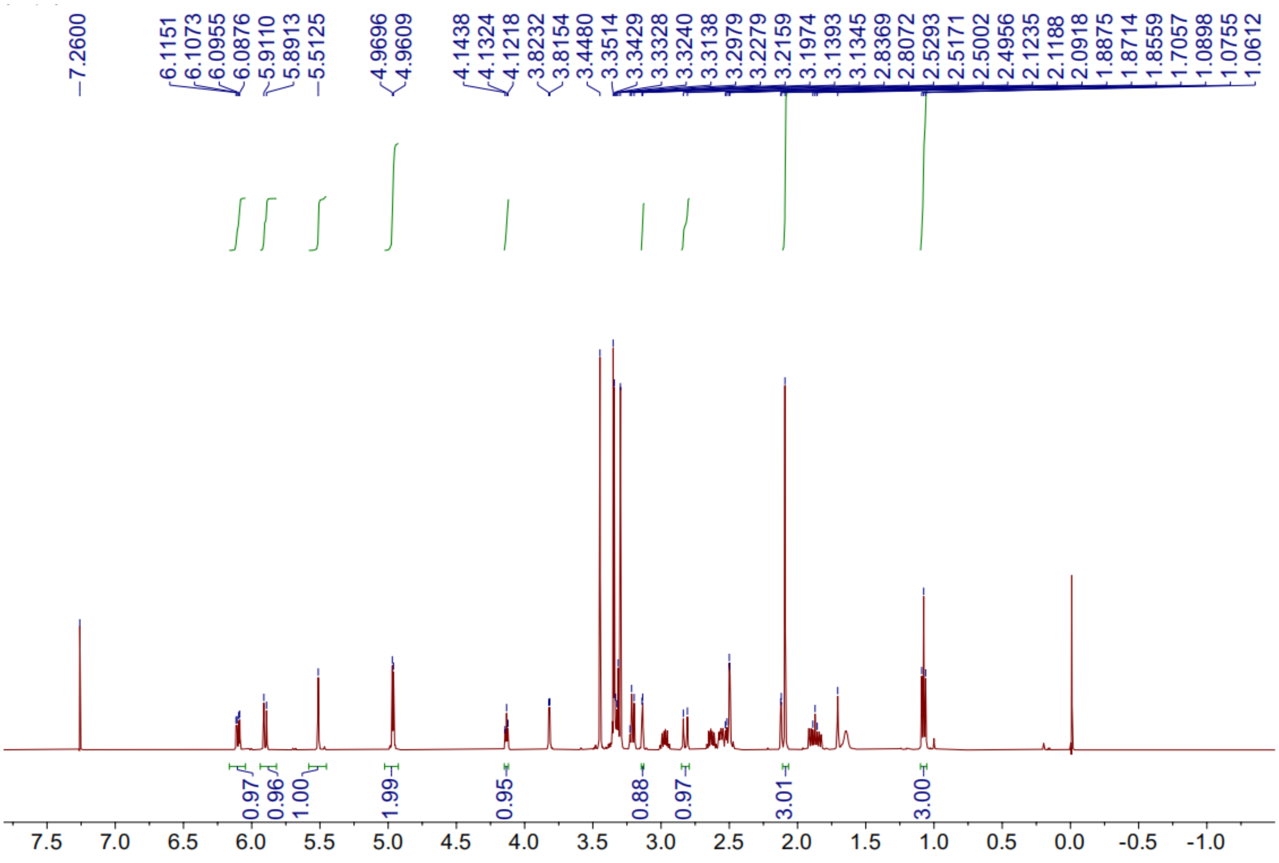

Figure S19: ^{1}H-NMR (500 MHz, $CDCl_3$) spectrum of compound 3

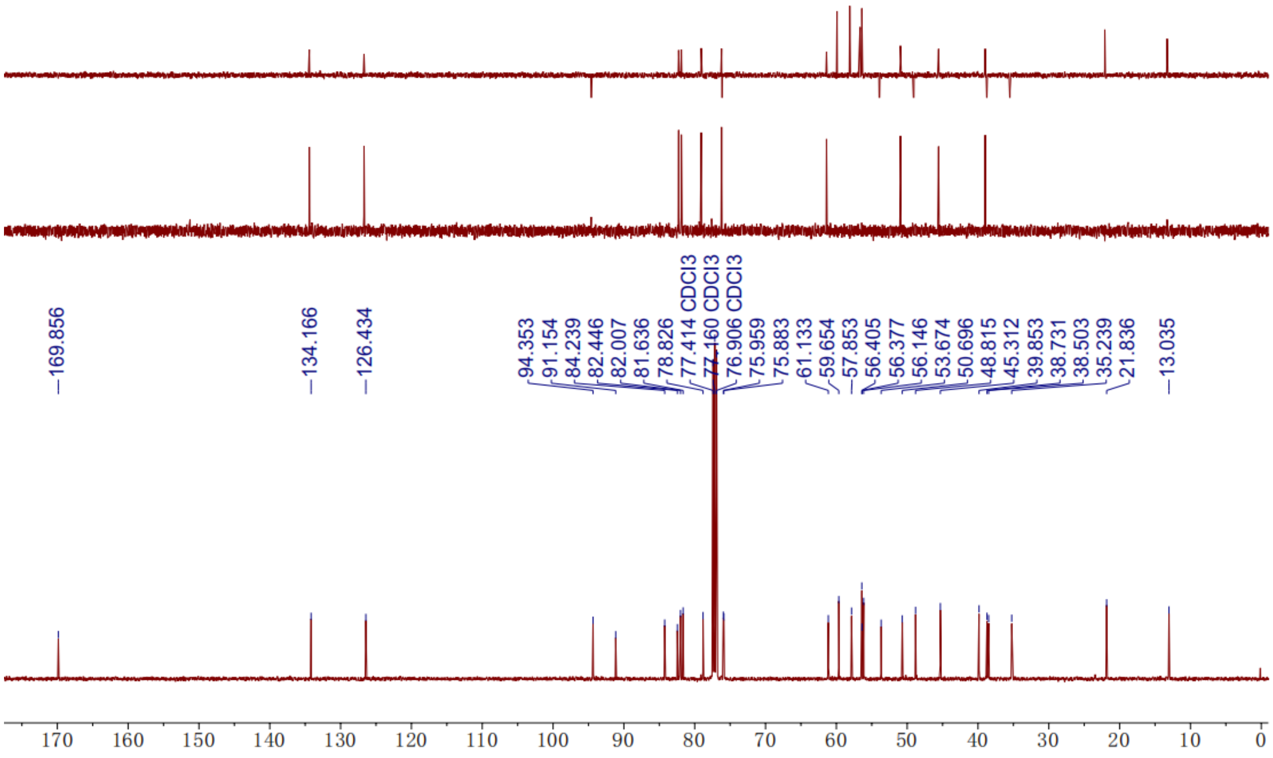

Figure S20: ^{13}C-NMR and DEPT (125 MHz, $CDCl_3$) spectrum of compound 3

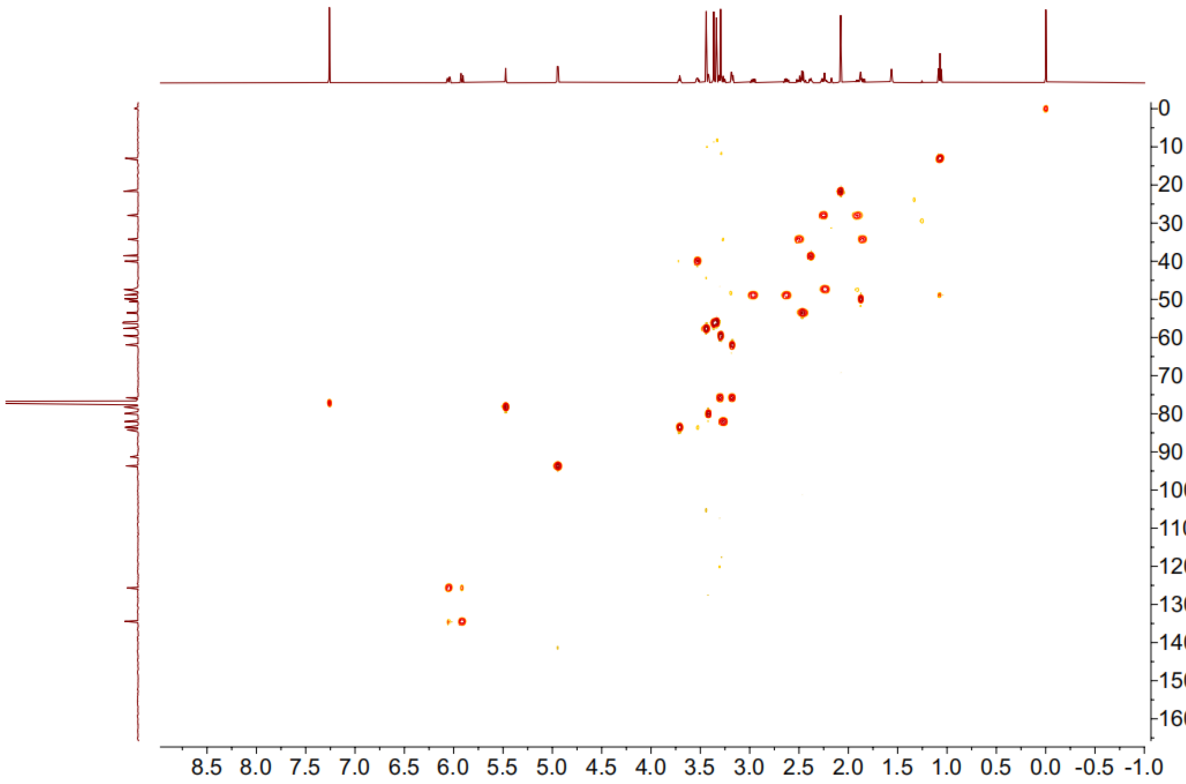

Figure S21: HSQC spectrum of compound 3

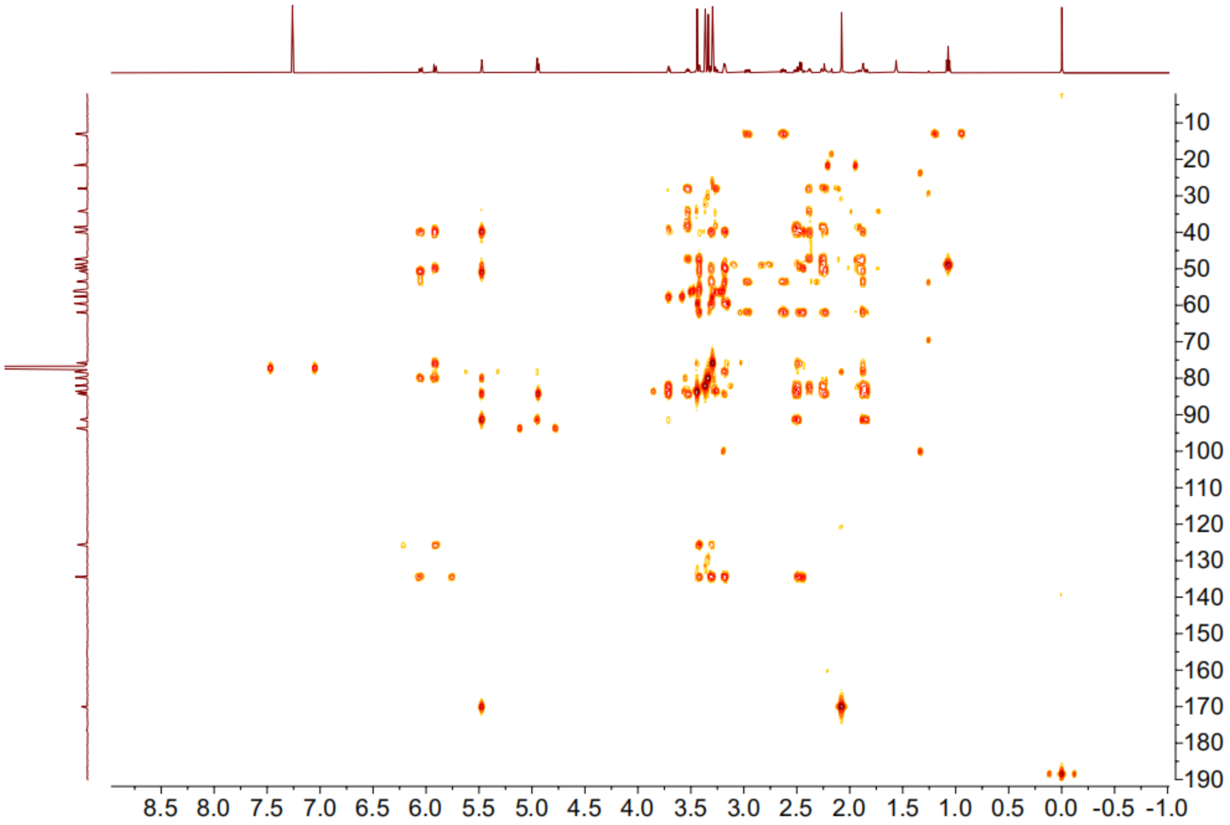

Figure S22: HMBC spectrum of compound 3

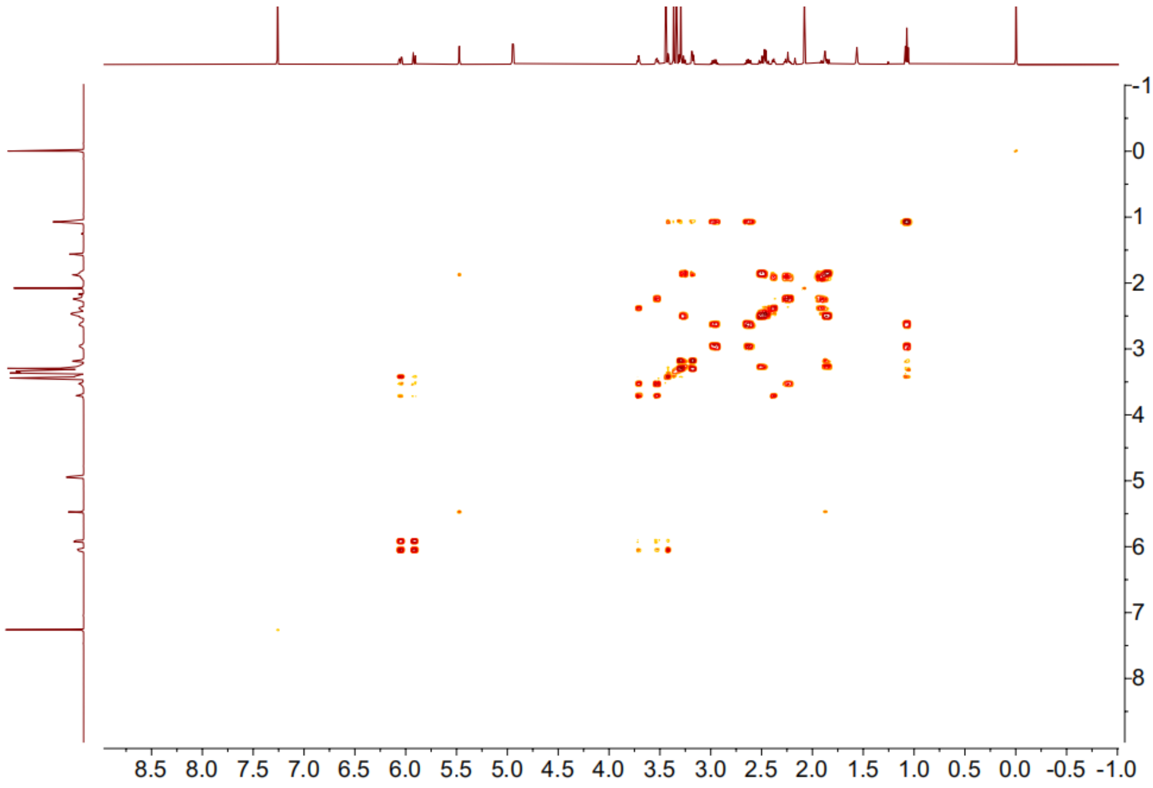

Figure S23: ^{1}H-^{1}H COSY spectrum of compound 3

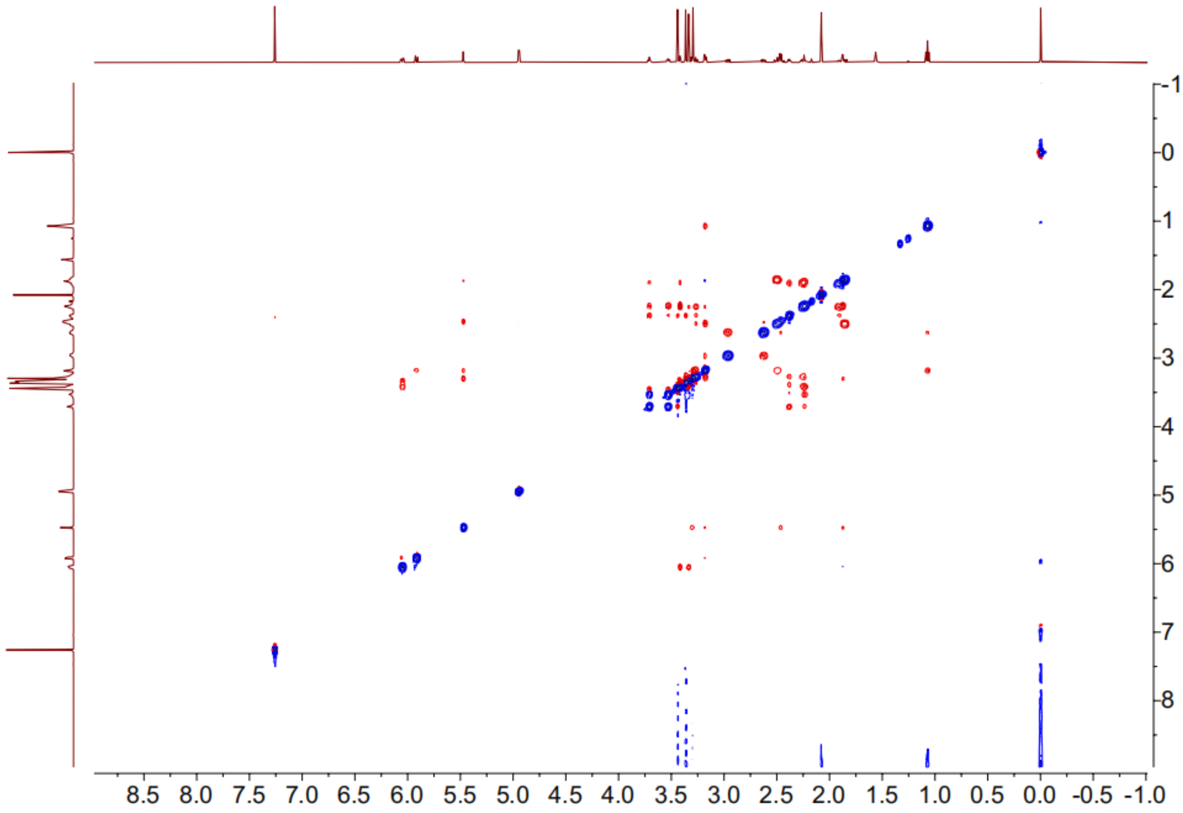

Figure S24: NOESY spectrum of compound 3

Formula Predictor Report - Ehr567.lcd

Data File: E:\DATA\2021\0422\Ehr567.lcd

Elmt	Val.	Min	Max	Elmt	Val.	Min	Max	Elmt	Val.	Min	Max	Elmt	Val.	Min	Max	Use Adduct
H	1	10	100	F	1	0	0	S	2	0	0	Br	1	0	0	H
2H	1	0	0	Na	1	0	0	Cl	1	0	0	Pd	2	0	0	Na
C	4	5	50	Mg	2	0	0	Co	2	0	0	Ag	1	0	0	
N	3	0	15	Si	4	0	0	Cu	2	0	0	I	3	0	0	
O	2	0	30	P	3	0	0	Se	2	0	0					

Error Margin (ppm):	5	DBE Range:	-2.0 - 100.0	Electron Ions:	both
HC Ratio:	unlimited	Apply N Rule:	no	Use MSn Info:	yes
Max Isotopes:	all	Isotope RI (%):	1.00	Isotope Res:	10000
MSn Iso RI (%):	75.00	MSn Logic Mode:	OR	Max Results:	20

Event#: 1 MS(E+) Ret. Time : 0.013 -> 0.440 Scan# : 3 -> 67

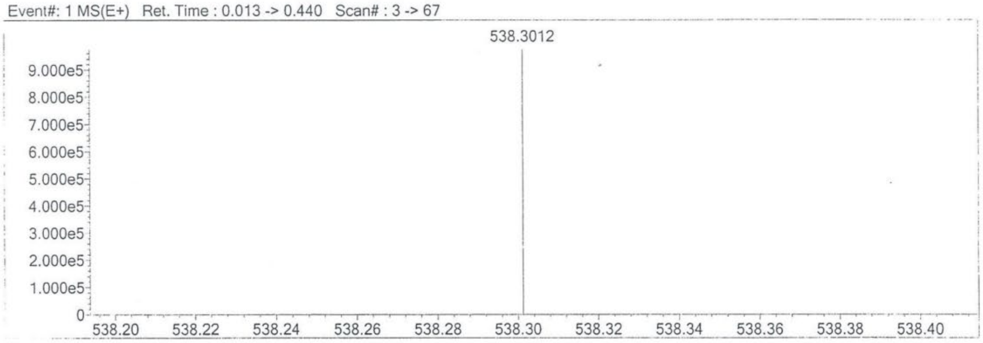

Measured region for 538.3012 m/z

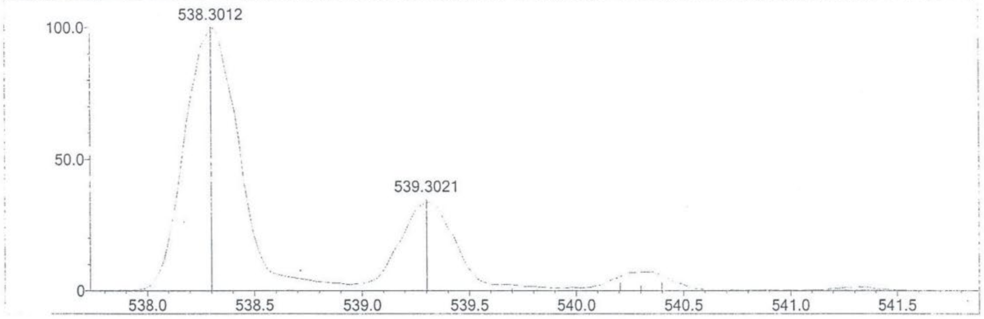

C28 H43 N O9 [M+H]+ : Predicted region for 538.3011 m/z

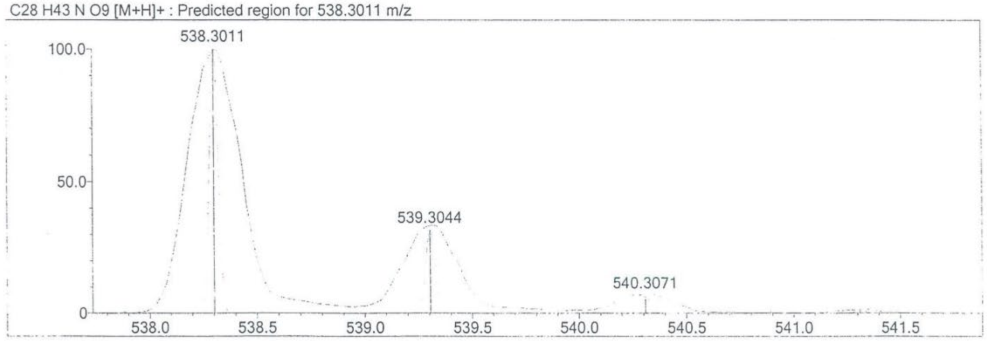

Formula (M)	Ion	Meas. m/z	Pred. m/z	Df. (mDa)	Df. (ppm)	DBE
C28 H43 N O9	[M+H]+	538.3012	538.3011	0.1	0.19	8.0

Figure S25: HRESIMS spectrum of compound 4

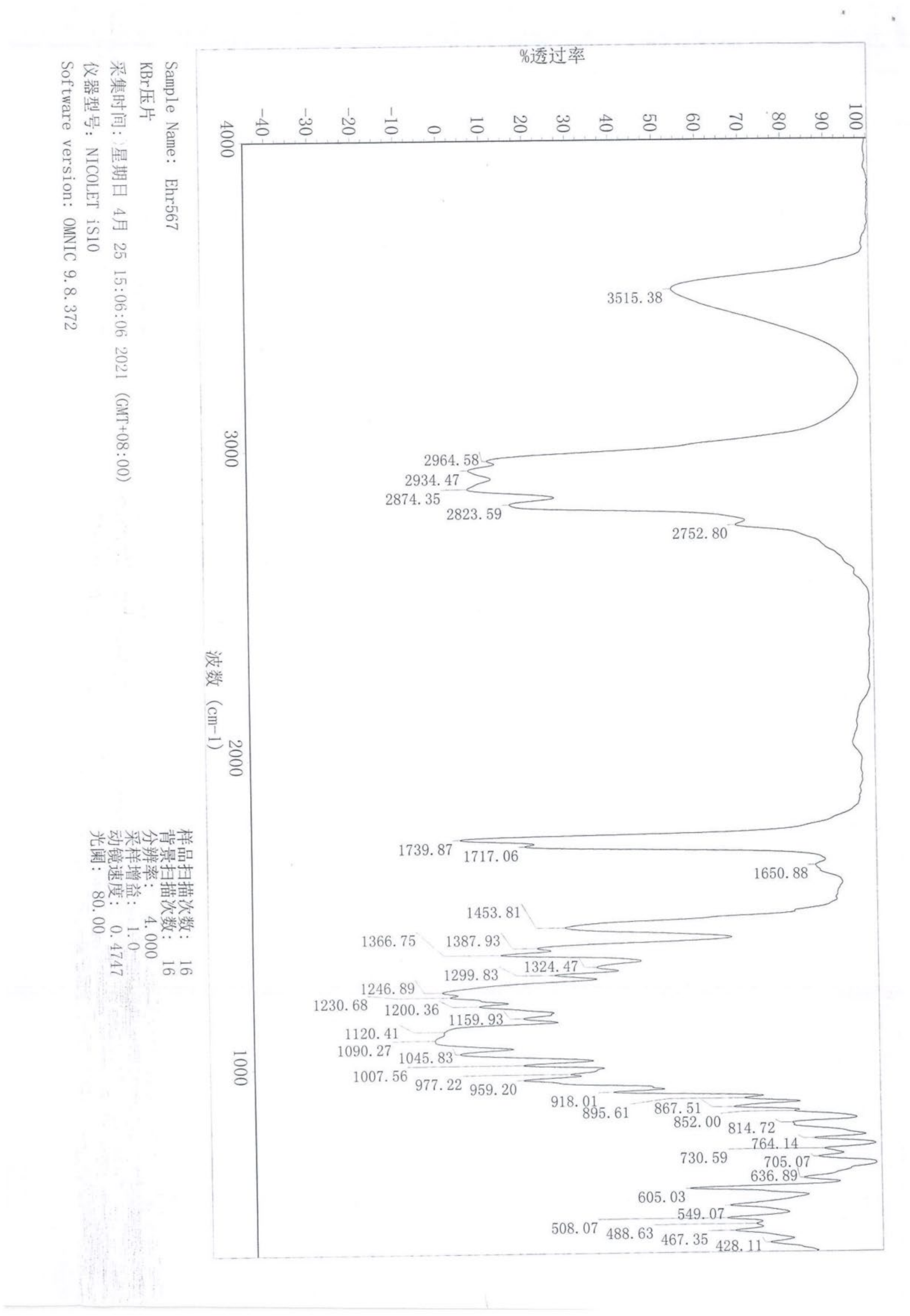

Figure S26: HRESIMS spectrum of compound 4

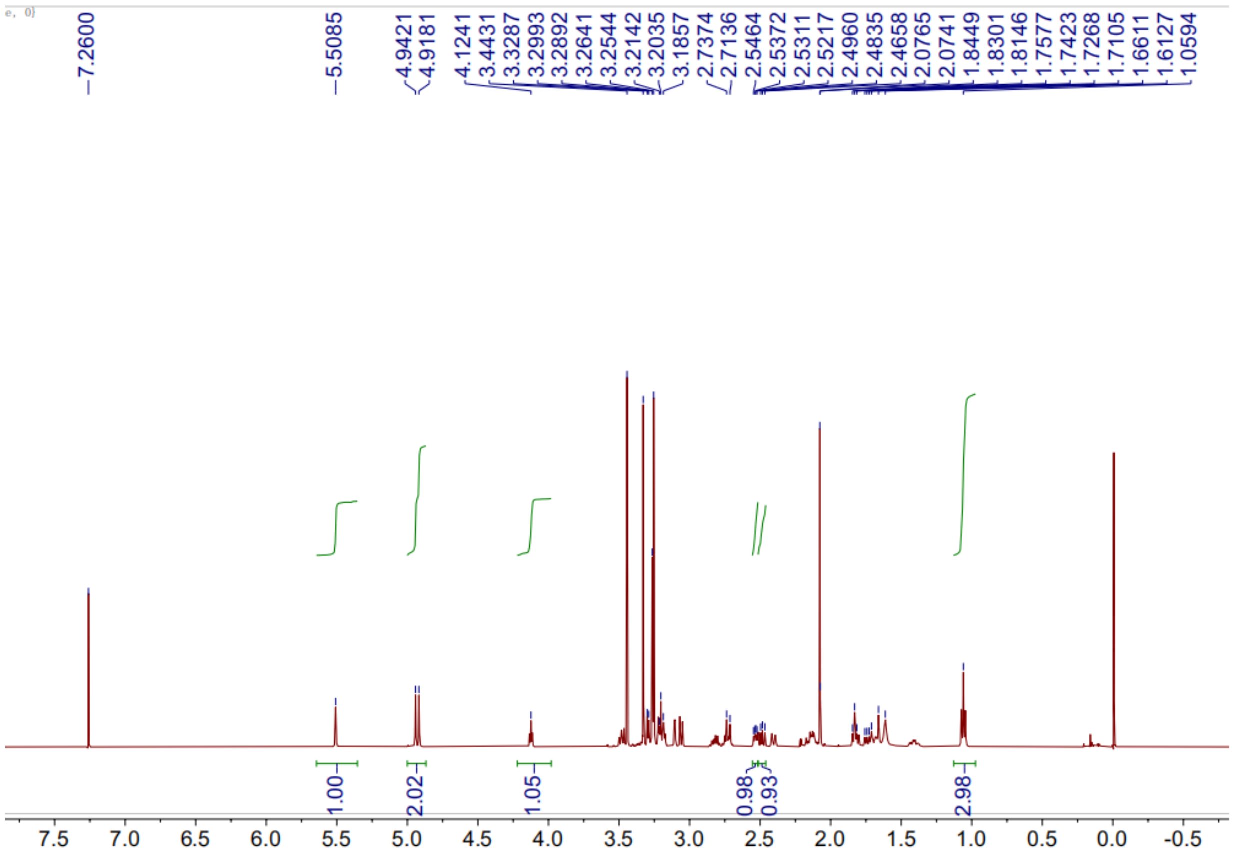

Figure S27: ^{1}H-NMR (500 MHz, $CDCl_3$) spectrum of compound 4

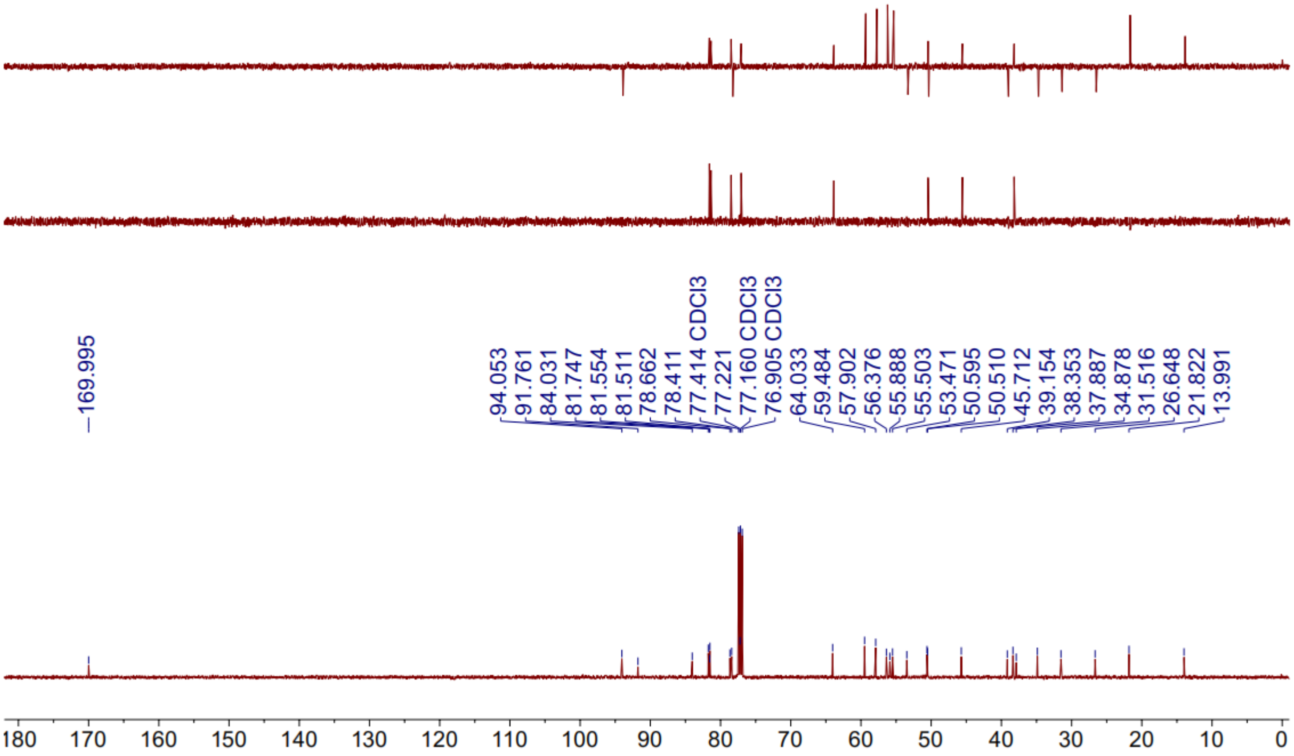

Figure S28: ^{13}C-NMR and DEPT (125 MHz, $CDCl_3$) spectrum of compound 4

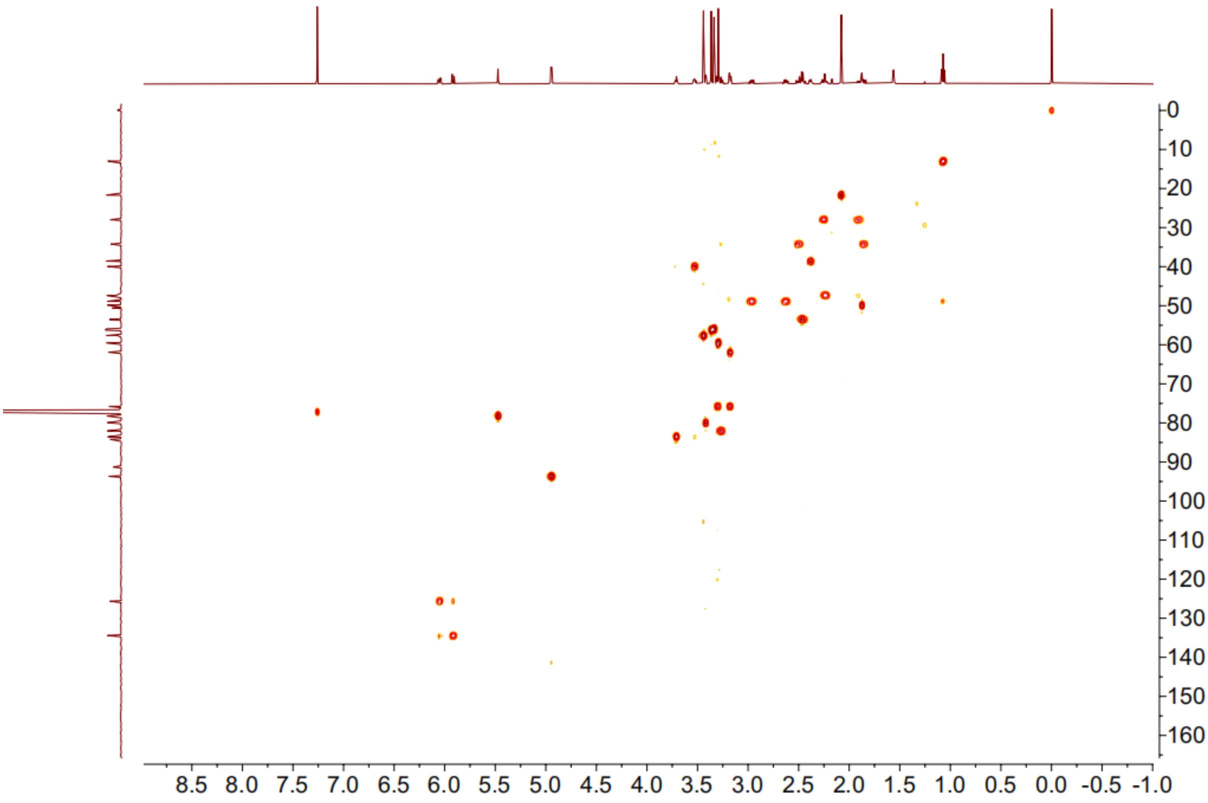

Figure S29: HSQC spectrum of compound 4

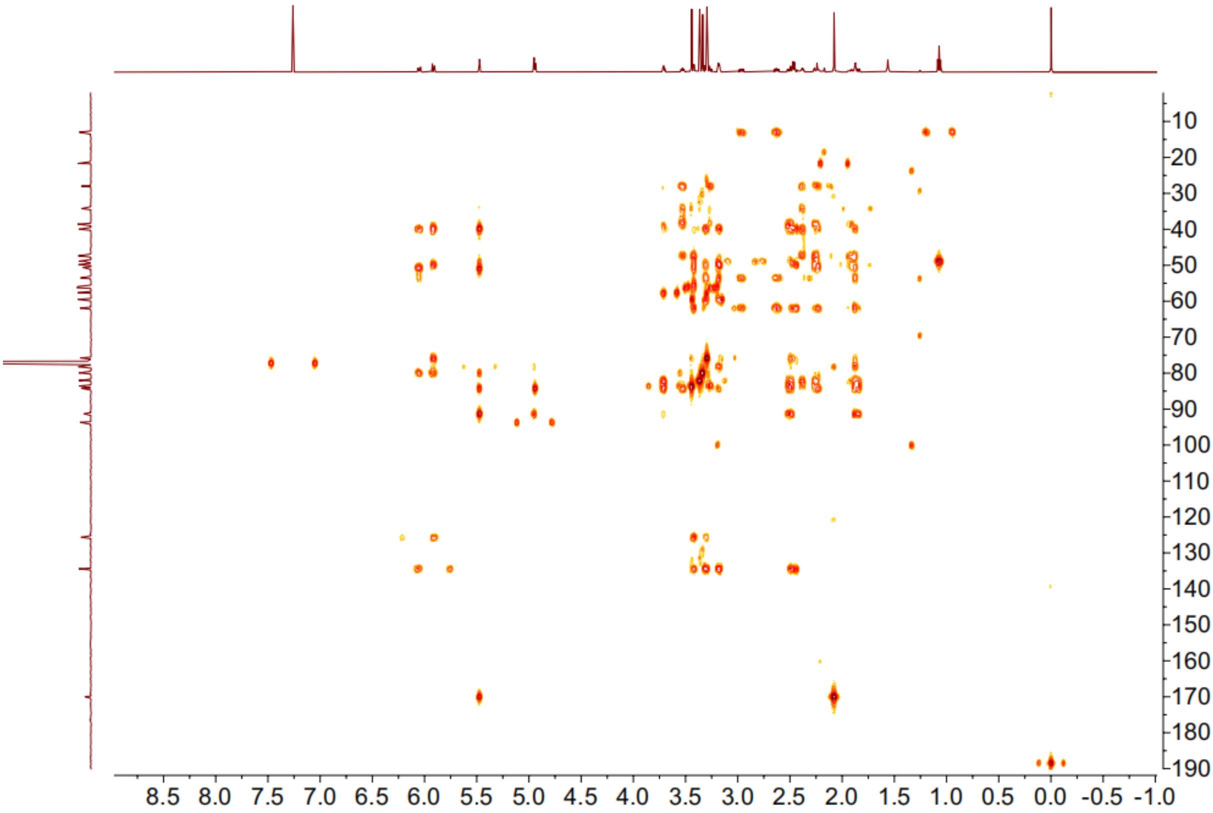

Figure S30: HMBC spectrum of compound 4

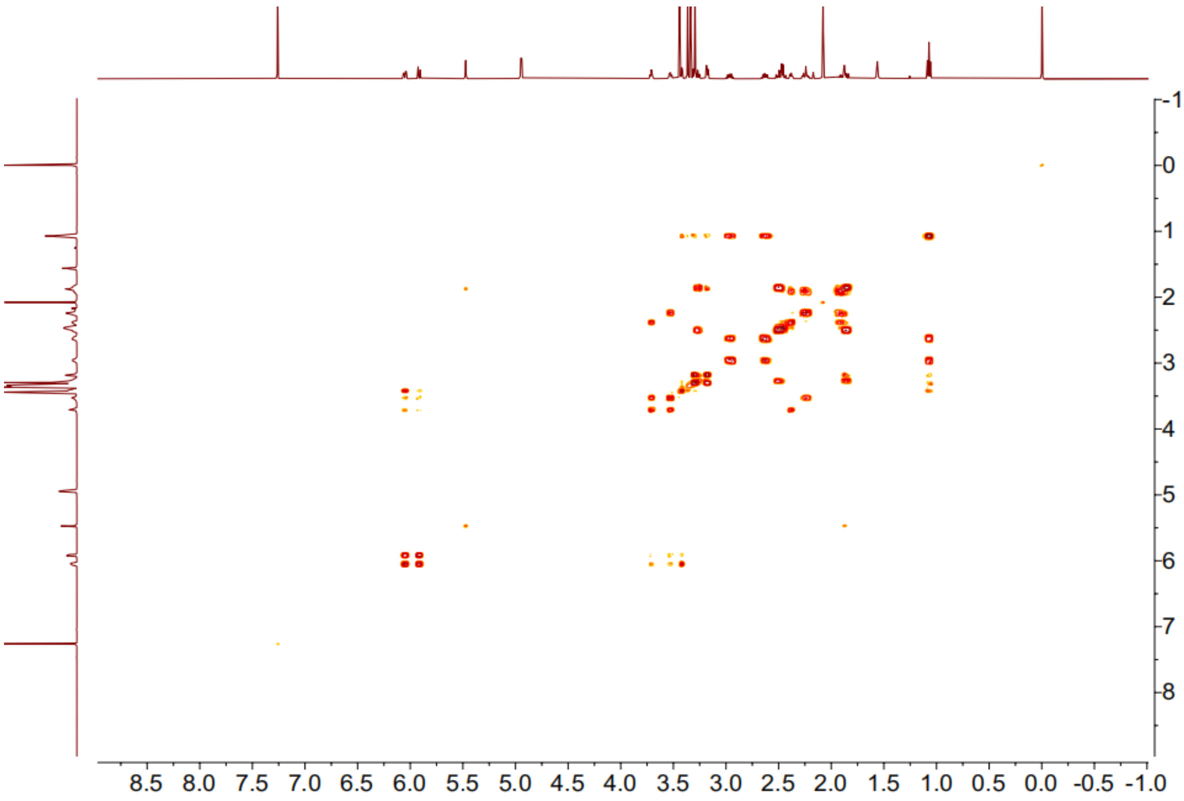

Figure S31: ^{1}H-^{1}H COSY spectrum of compound 4

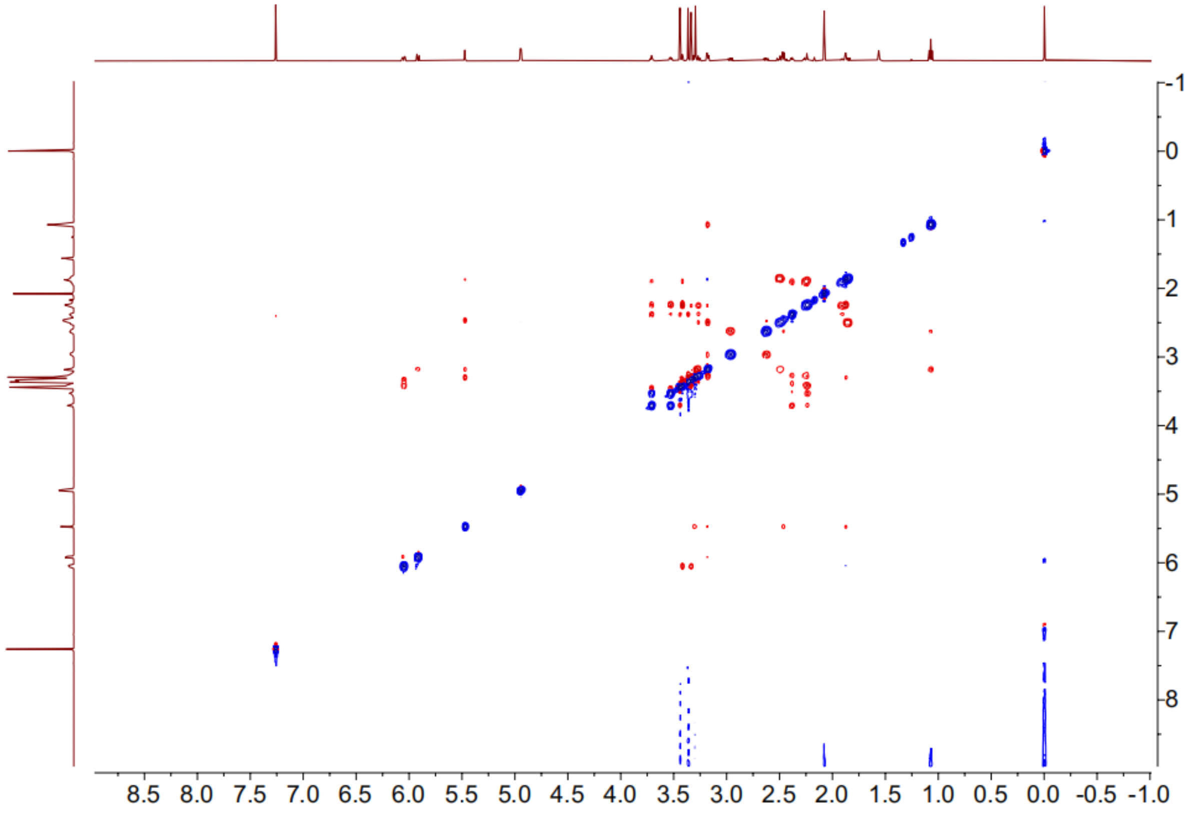

Figure S32: NOESY spectrum of compound 4

Formula Predictor Report - Ehr553.lcd

Page 1 of 1

Data File: E:\DATA\2021\0325\Ehr553.lcd

Elmt	Val.	Min	Max	Elmt	Val.	Min	Max	Elmt	Val.	Min	Max	Elmt	Val.	Min	Max	Use Adduct
H	1	10	100	F	1	0	0	S	2	0	0	Br	1	0	0	H
2H	1	0	0	Na	1	0	0	Cl	1	0	0	Pd	2	0	0	Na
C	4	5	50	Mg	2	0	0	Co	2	0	0	Ag	1	0	0	
N	3	0	10	Si	4	0	0	Cu	2	0	0	I	3	0	0	
O	2	0	30	P	3	0	0	Se	2	0	0					

Error Margin (ppm): 5
HC Ratio: unlimited
Max Isotopes: all
MSn Iso RI (%): 75.00

DBE Range: -2.0 - 100.0
Apply N Rule: yes
Isotope RI (%): 1.00
MSn Logic Mode: OR

Electron Ions: both
Use MSn Info: yes
Isotope Res: 10000
Max Results: 20

Event#: 1 MS(E+) Ret. Time : 0.440 -> 0.720 Scan# : 67 -> 109

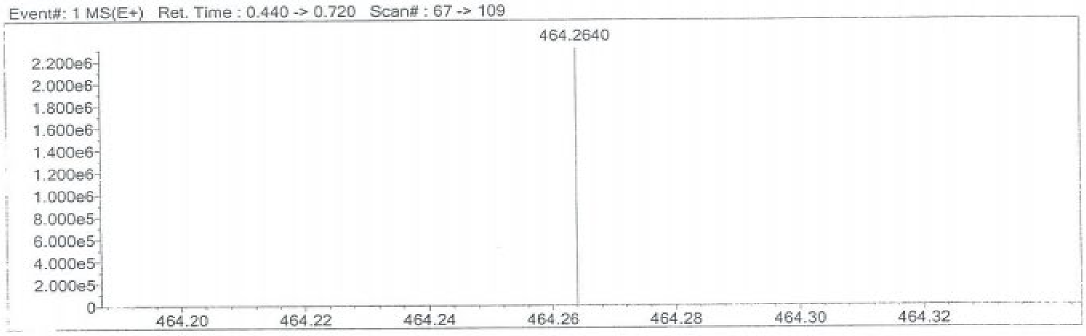

Measured region for 464.2640 m/z

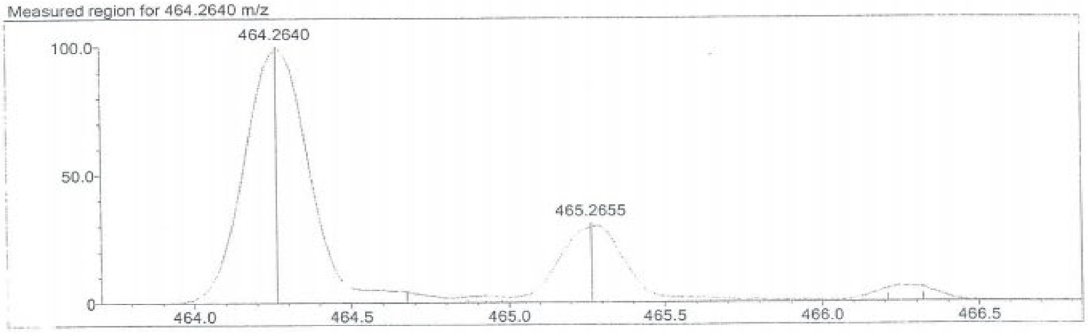

C25 H37 N O7 [M+H]+ : Predicted region for 464.2643 m/z

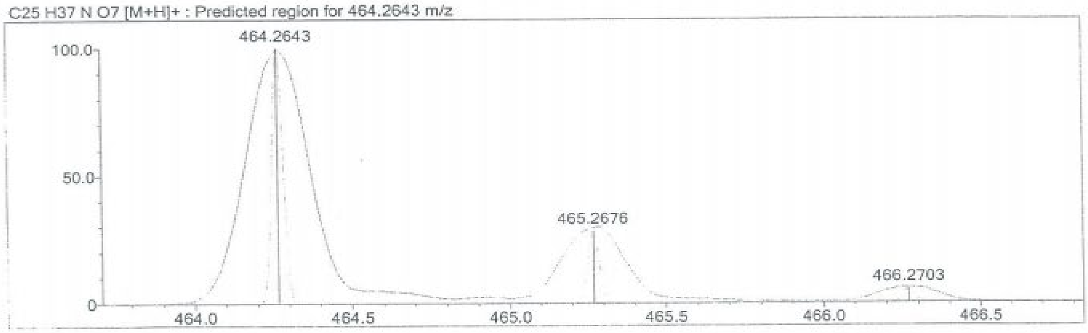

Formula (M)	Ion	Meas. m/z	Pred. m/z	Df. (mDa)	Df. (ppm)	DBE
C25 H37 N O7	[M+H]+	464.2640	464.2643	-0.3	-0.65	8.0

Figure S33: HRESIMS spectrum of compound 5

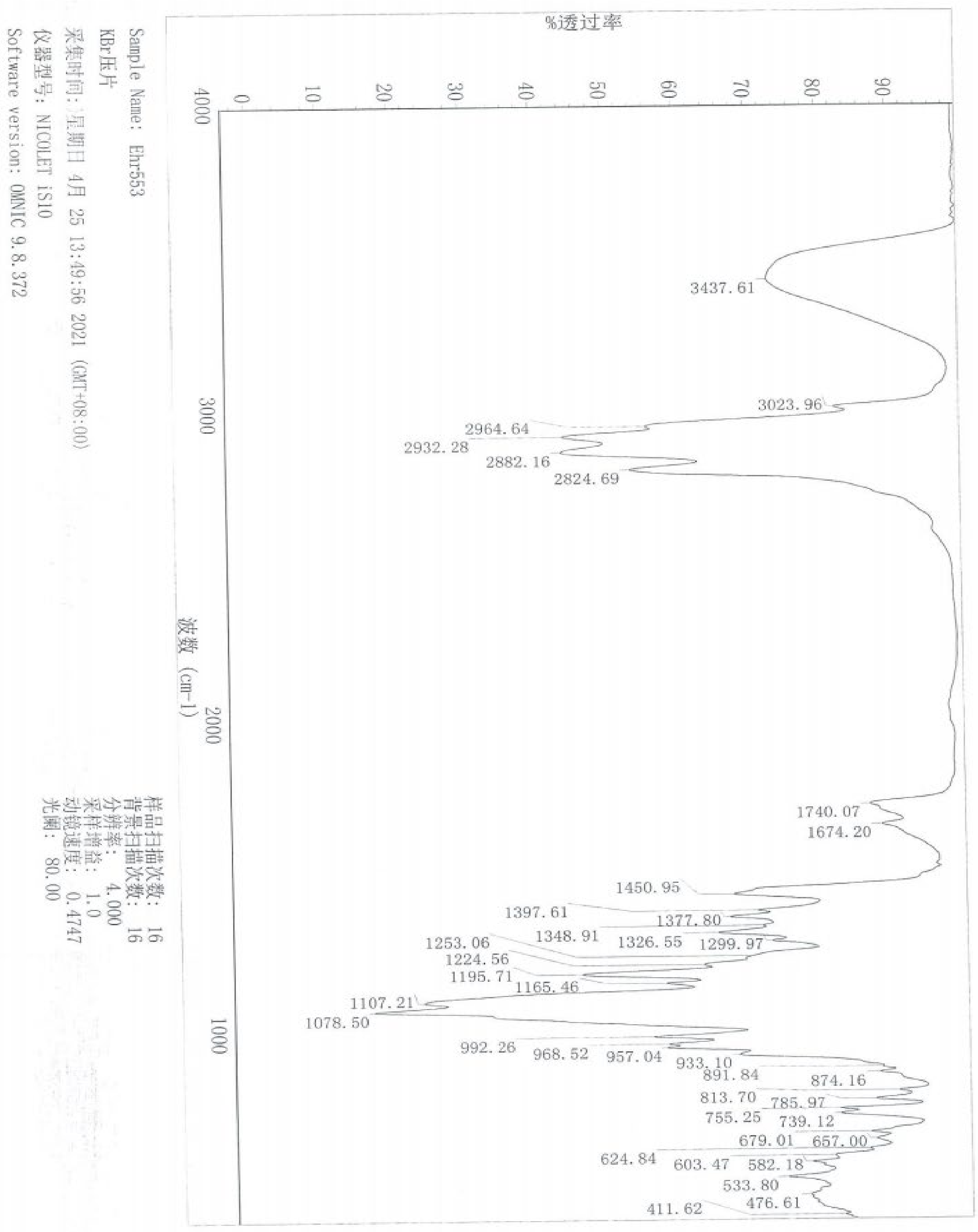

Figure S34: IR spectrum of compound 5

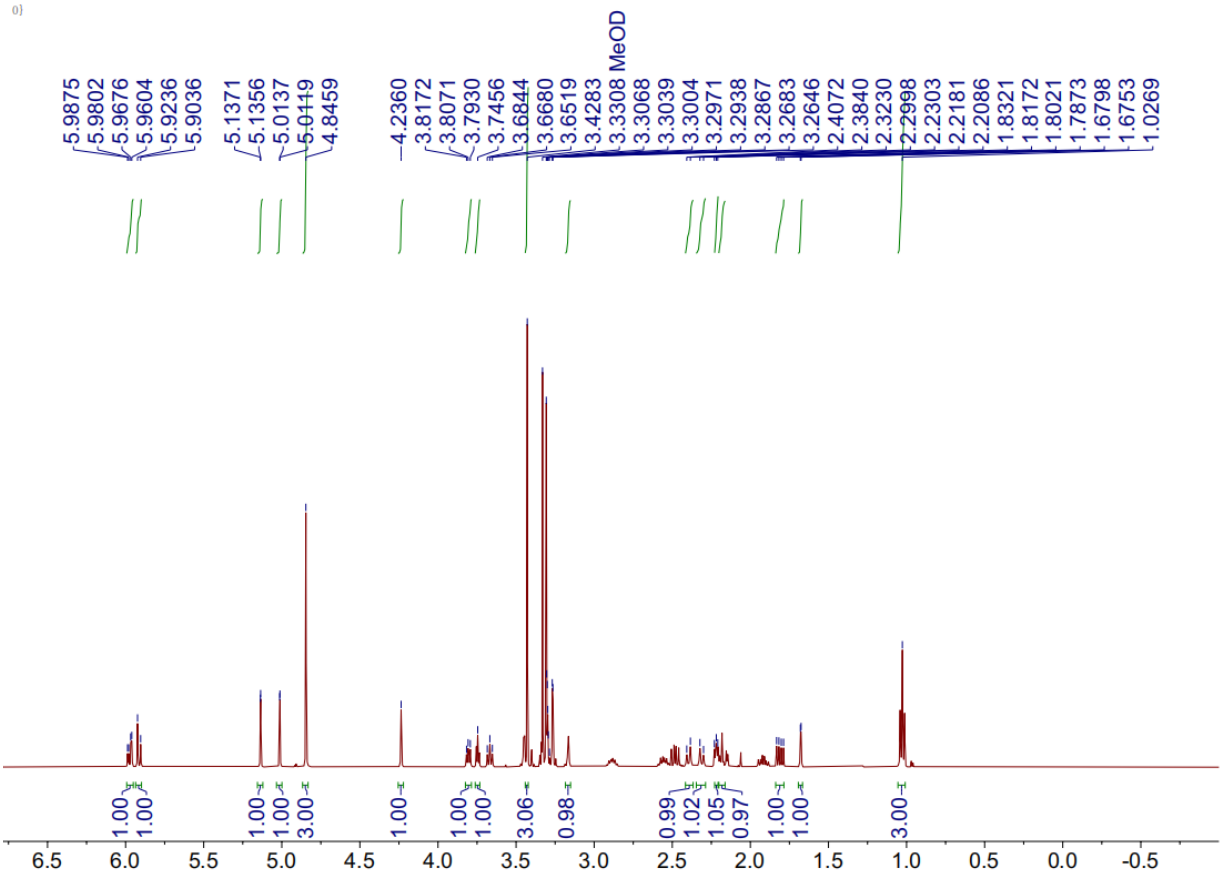

Figure S35: The ^{1}H-NMR Spectrum ($CDCl_3$) of Compound 5

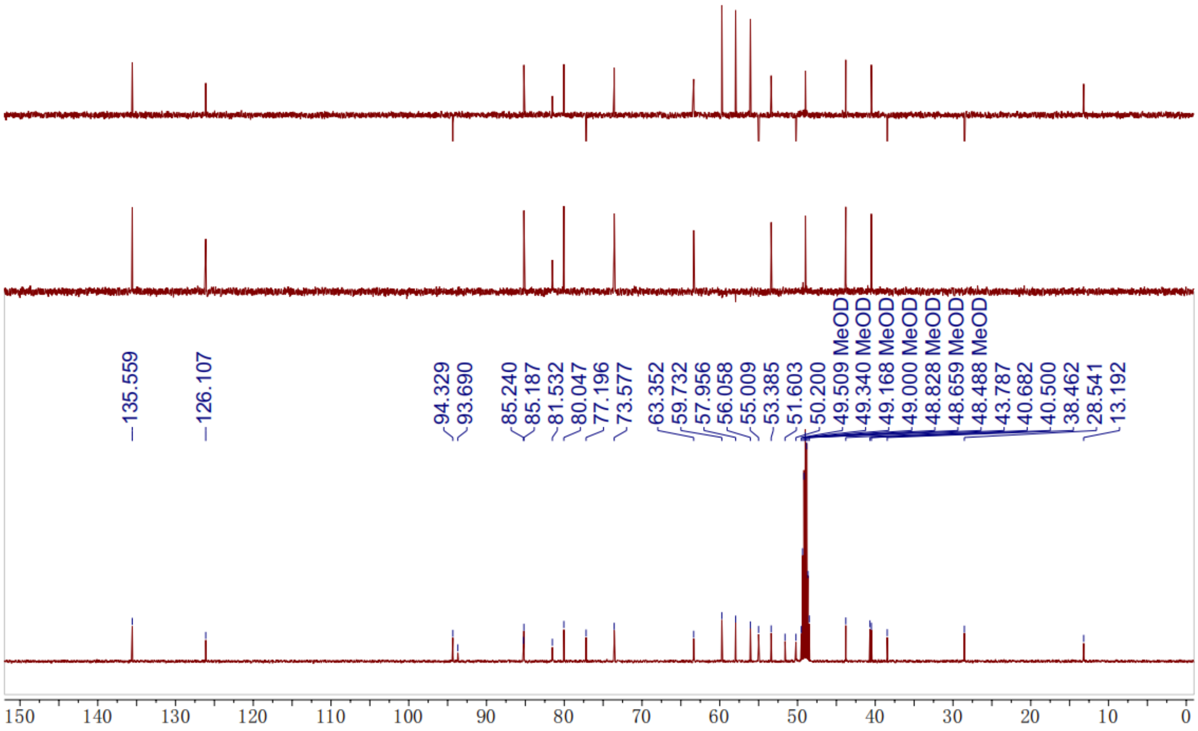

Figure S36: ^{13}C-NMR and DEPT (125 MHz, CD_3OD) spectrum of compound 5

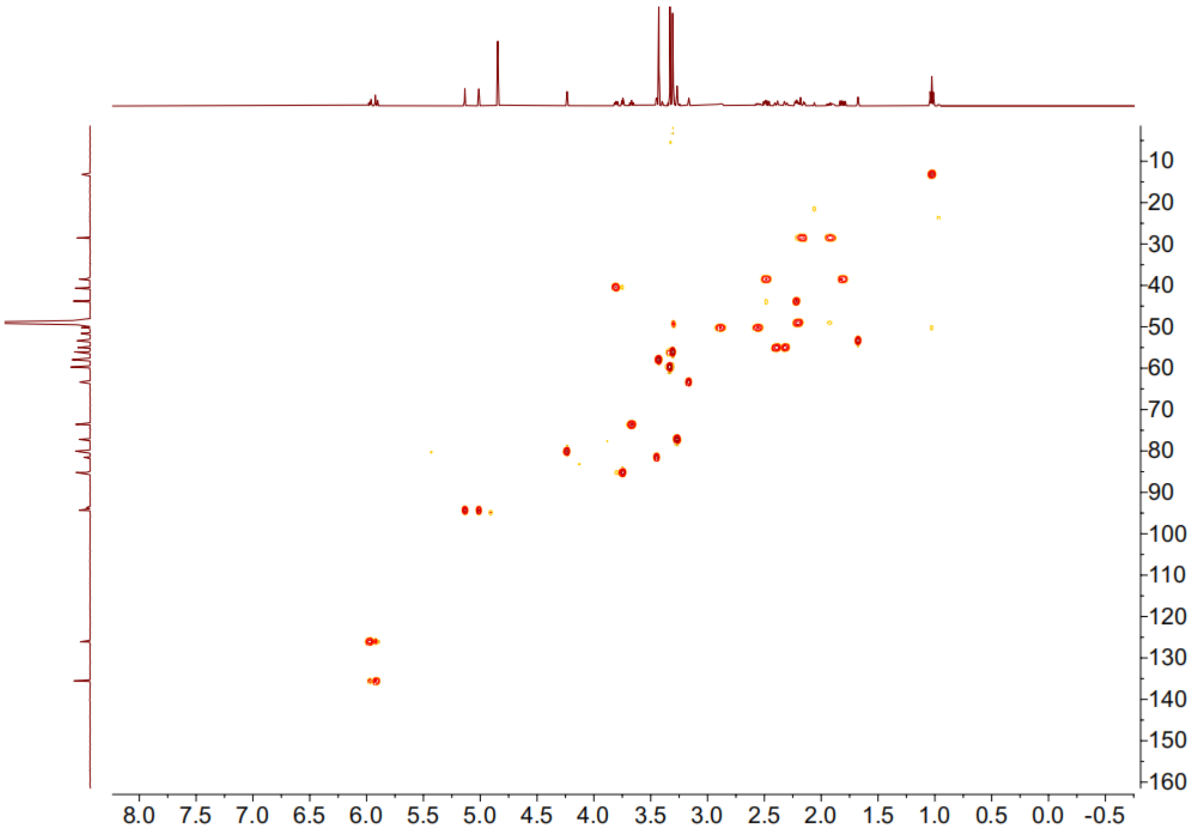

Figure S37: HSQC spectrum of compound 5

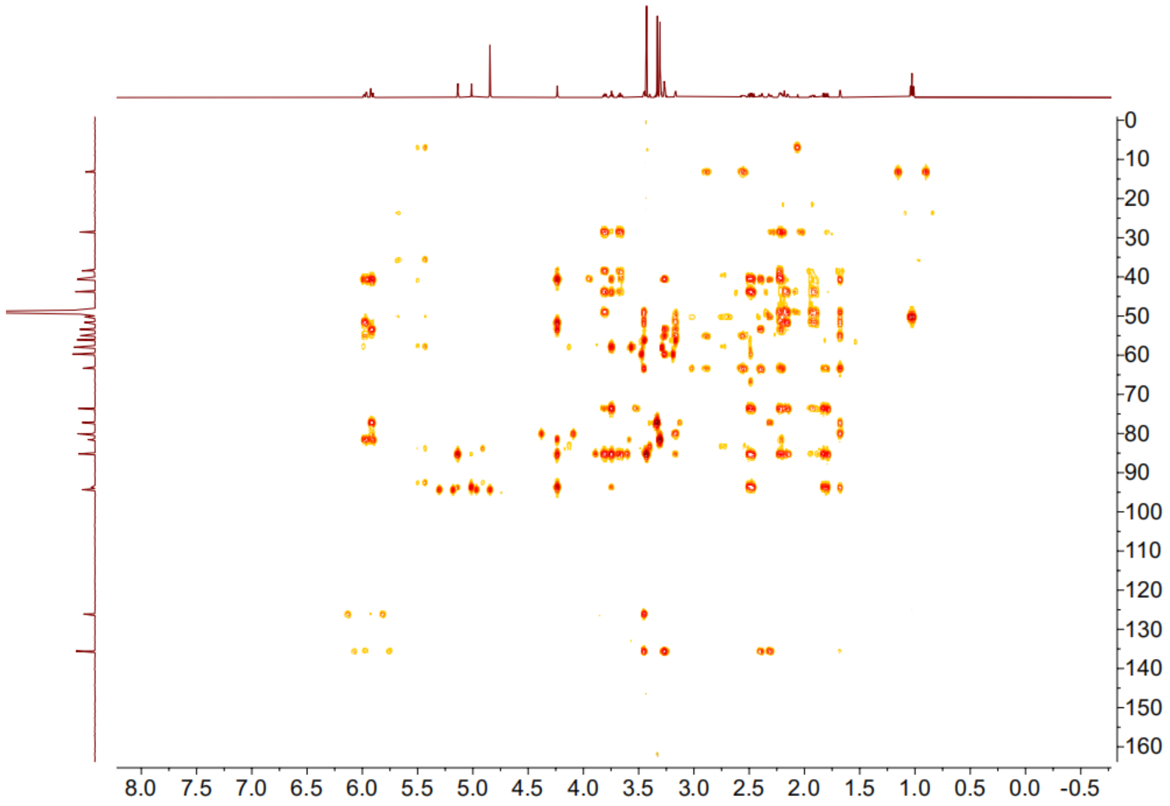

Figure S38: HMBC spectrum of compound 5

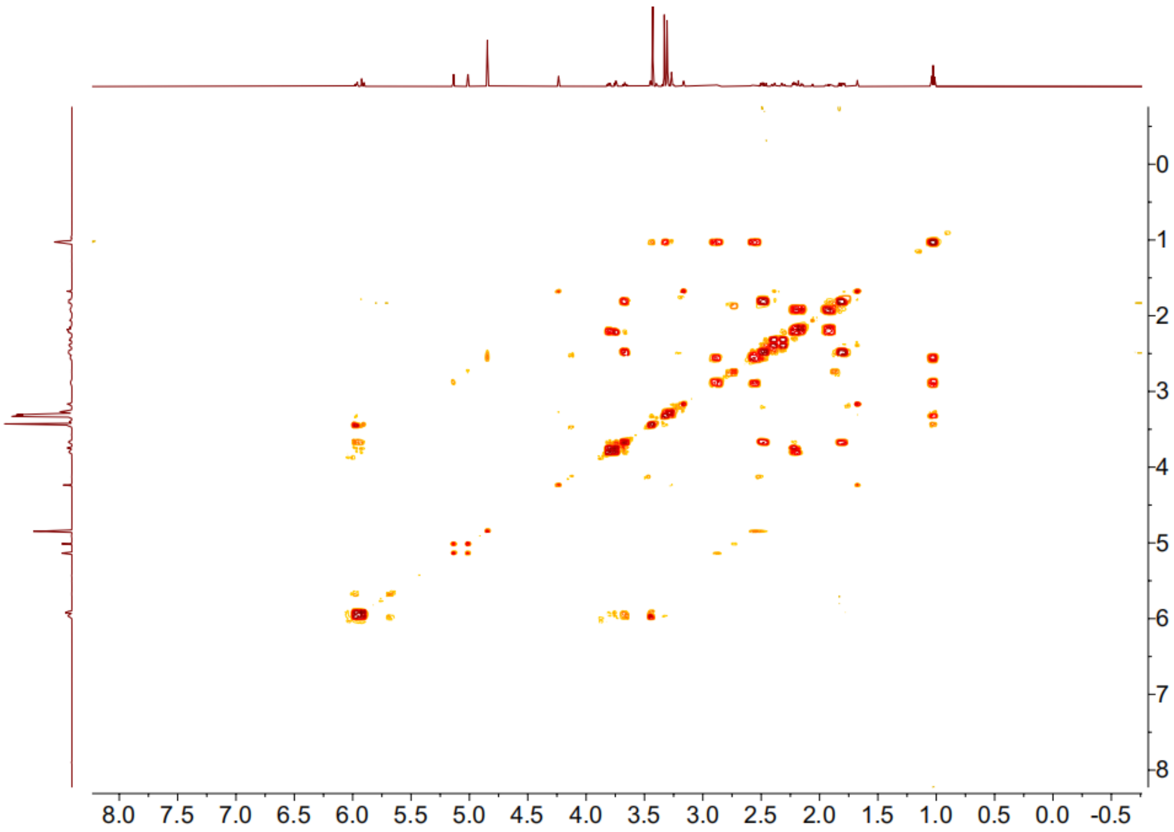

Figure S39: ^{1}H-^{1}H COSY spectrum of compound 5

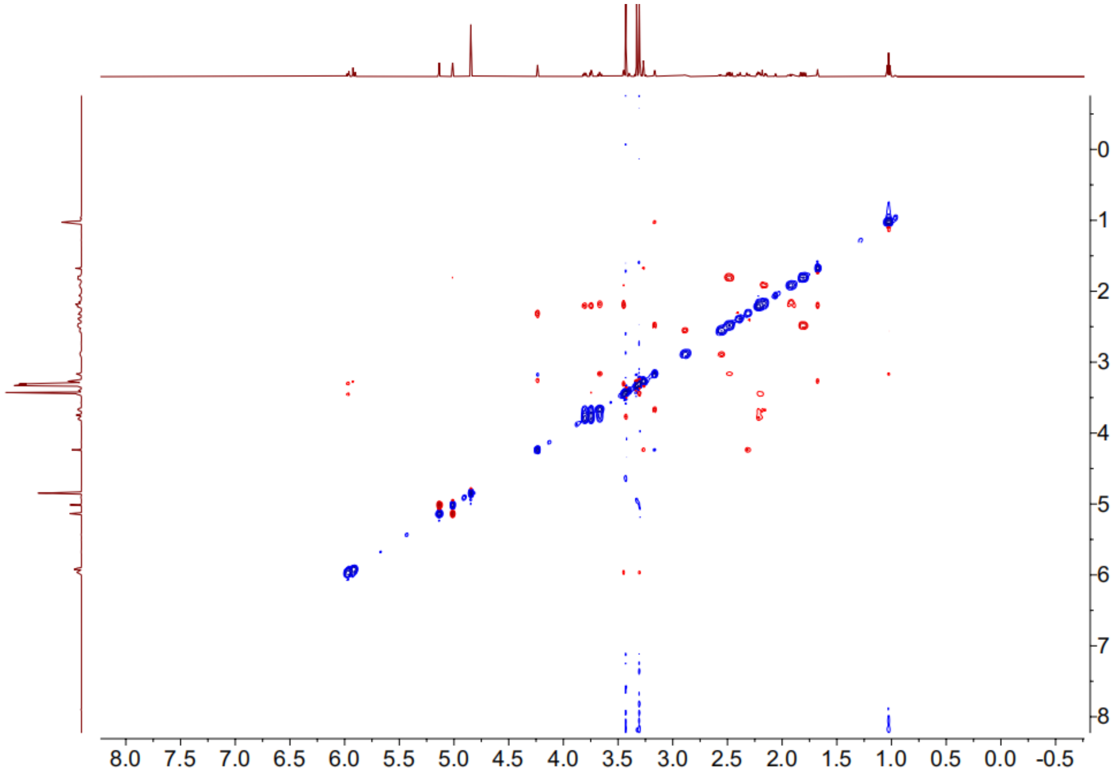

Figure S40: NOESY spectrum of compound 5

Data File: E:\DATA\2021\0415\Ehr559.lcd

Elmt	Val.	Min	Max	Elmt	Val.	Min	Max	Elmt	Val.	Min	Max	Elmt	Val.	Min	Max	Use Adduct
H	1	10	100	F	1	0	0	S	2	0	0	Br	1	0	5	H
2H	1	0	0	Na	1	0	0	Cl	1	0	0	Pd	2	0	0	Na
C	4	5	50	Mg	2	0	0	Co	2	0	0	Ag	1	0	0	
N	3	0	20	Si	4	0	0	Cu	2	0	0	I	3	0	0	
O	2	0	30	P	3	0	0	Se	2	0	0					

Error Margin (ppm):	5	DBE Range:	-2.0 - 100.0	Electron Ions:	both
HC Ratio:	unlimited	Apply N Rule:	no	Use MSn Info:	yes
Max Isotopes:	all	Isotope RI (%):	1.00	Isotope Res:	10000
MSn Iso RI (%):	75.00	MSn Logic Mode:	OR	Max Results:	20

Event#: 1 MS(E+) Ret. Time : 0.320 -> 0.360 Scan# : 49 -> 55

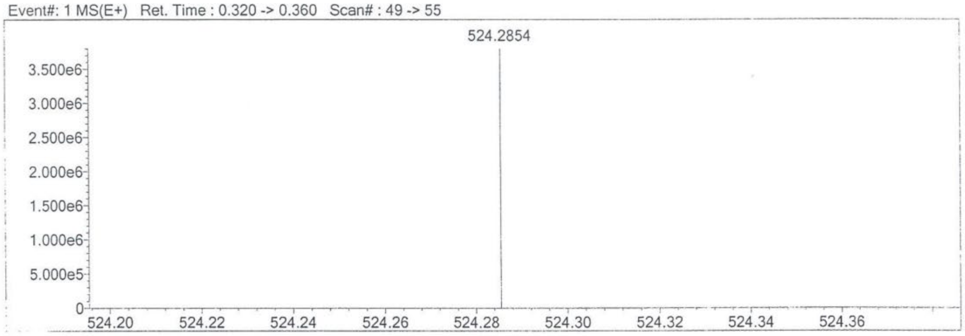

Measured region for 524.2854 m/z

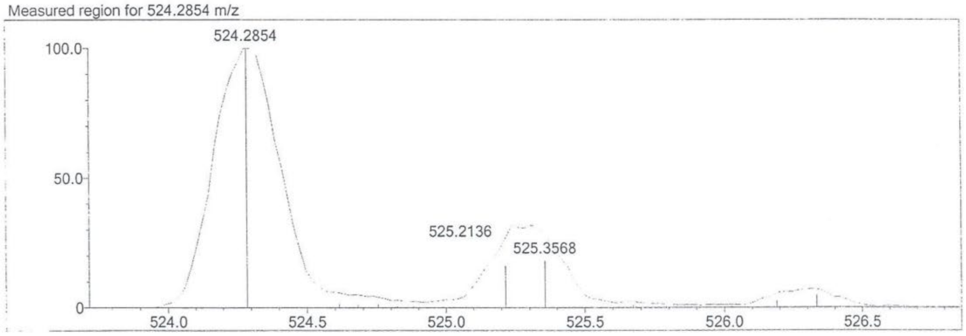

C27 H41 N O9 [M+H]+ : Predicted region for 524.2854 m/z

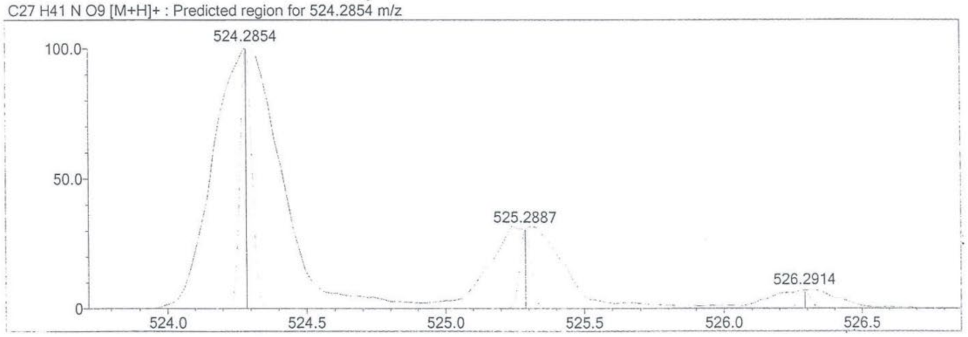

Formula (M)	Ion	Meas. m/z	Pred. m/z	Df. (mDa)	Df. (ppm)	DBE
C27 H41 N O9	[M+H]+	524.2854	524.2854	-0.0	0.00	8.0

Figure S41: HRESIMS spectrum of compound 6

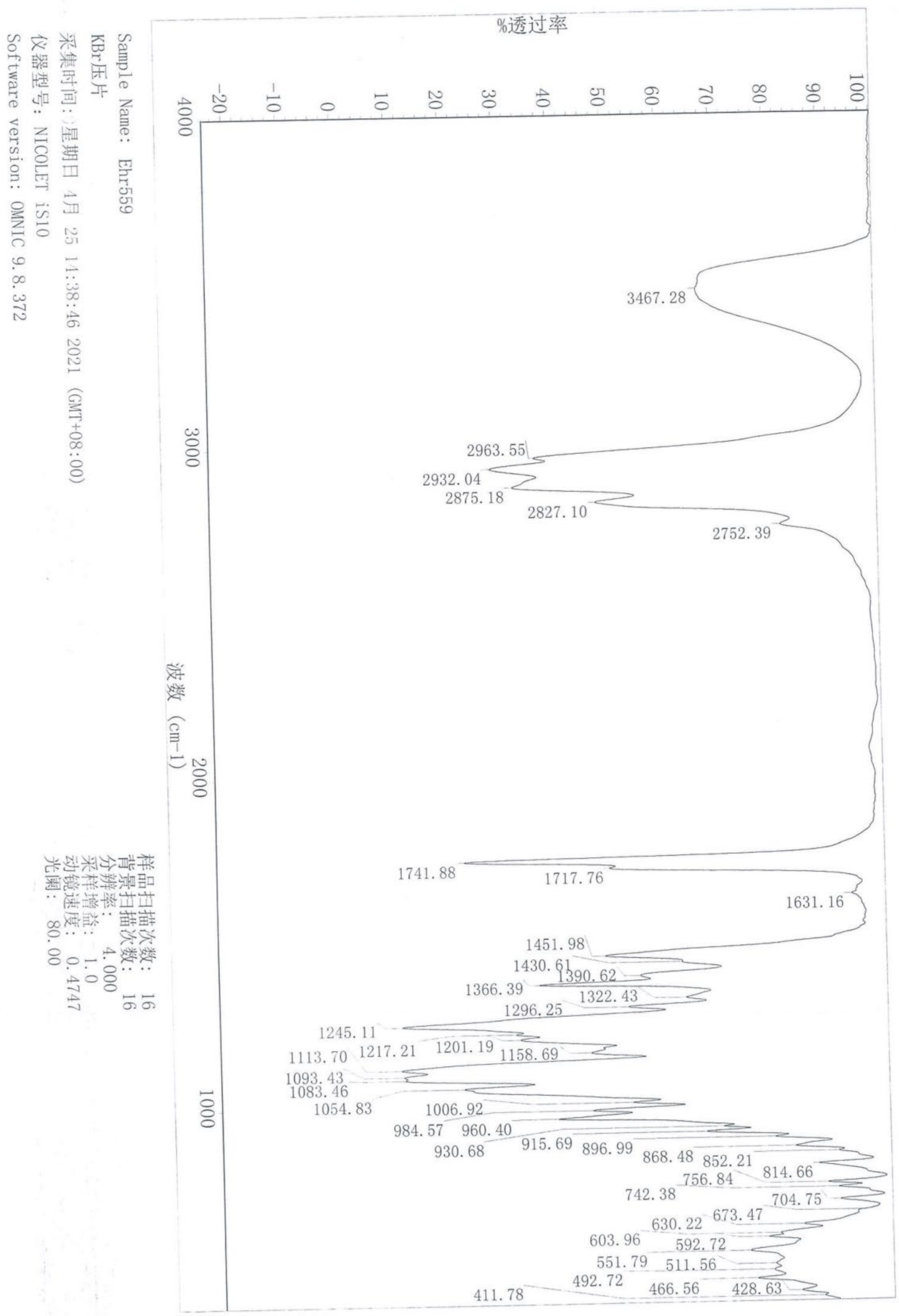

Figure S42: IR spectrum of compound 6

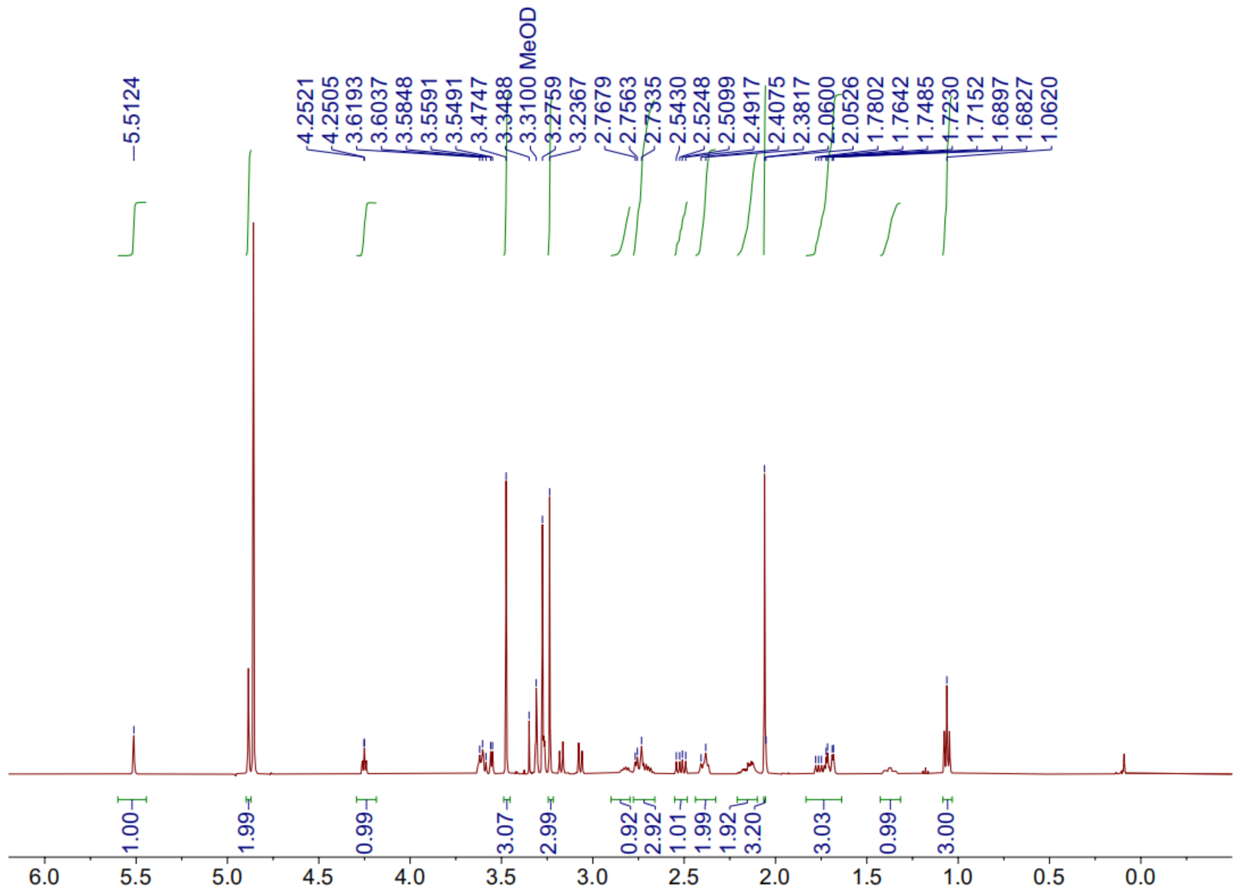

Figure S43: ^{1}H-NMR (500 MHz, CD_3OD) spectrum of compound 6

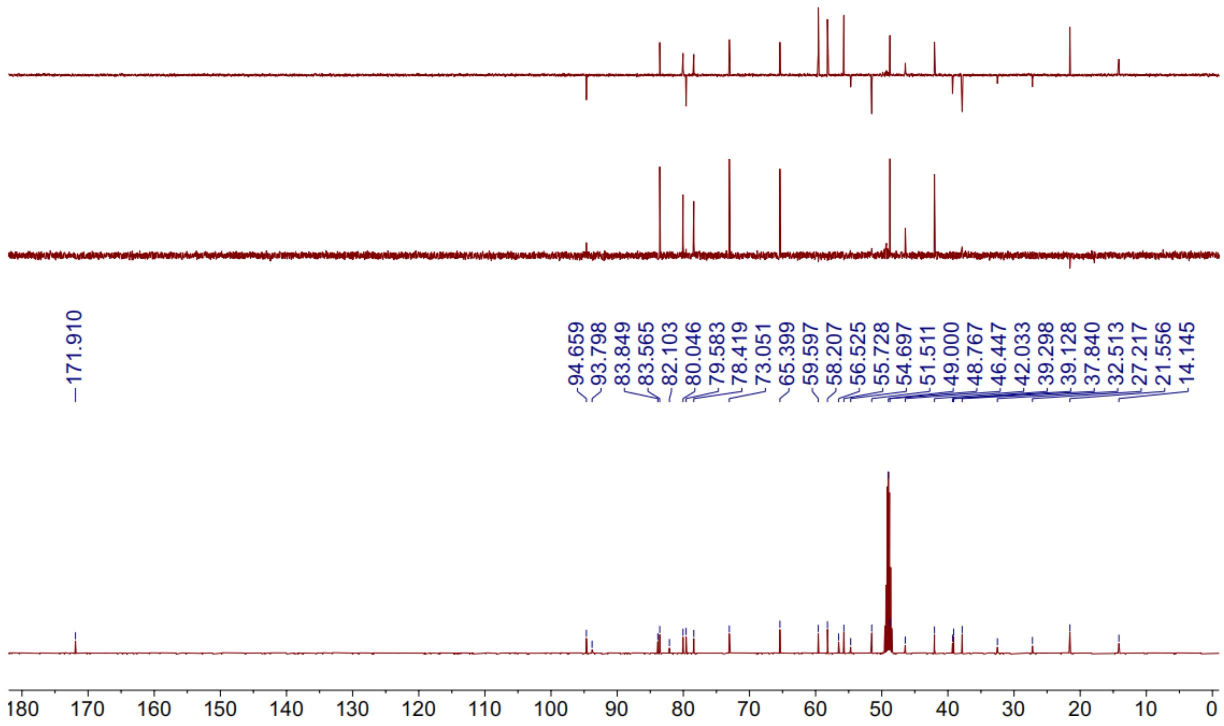

Figure S44: ^{13}C-NMR (125 MHz, CD_3OD) spectrum of compound 6

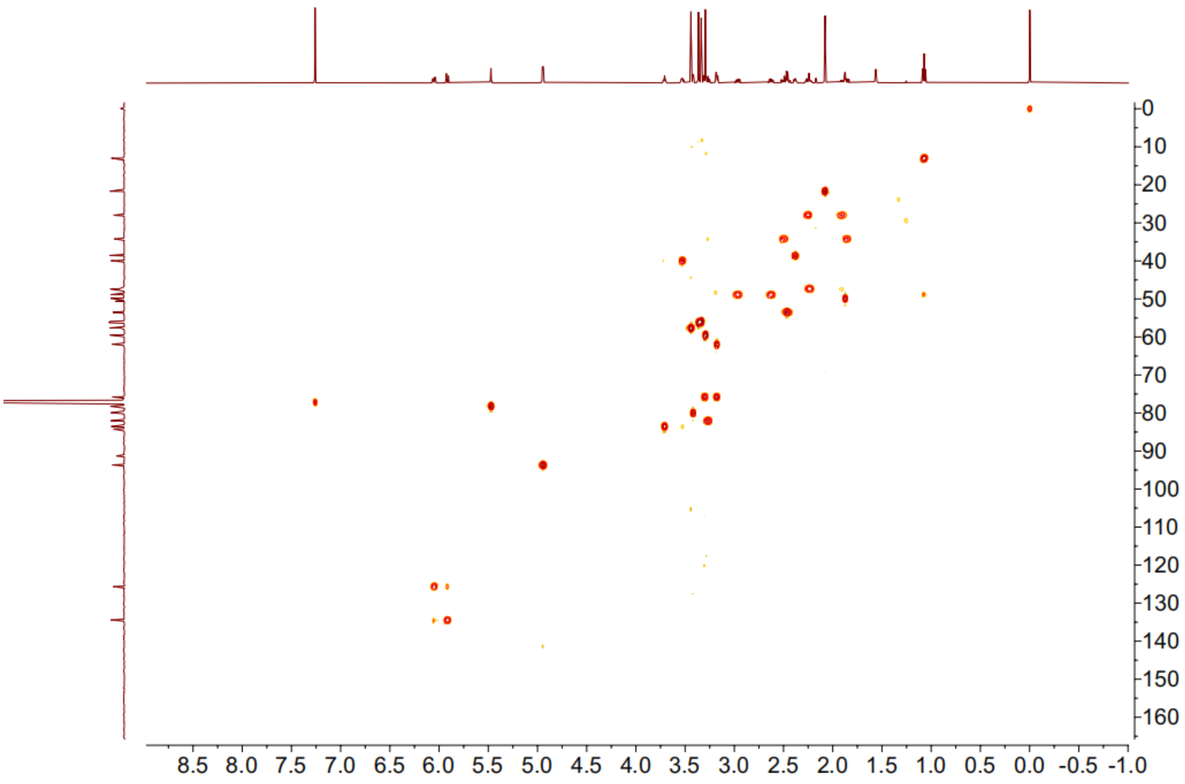

Figure S45: HSQC spectrum of compound 6

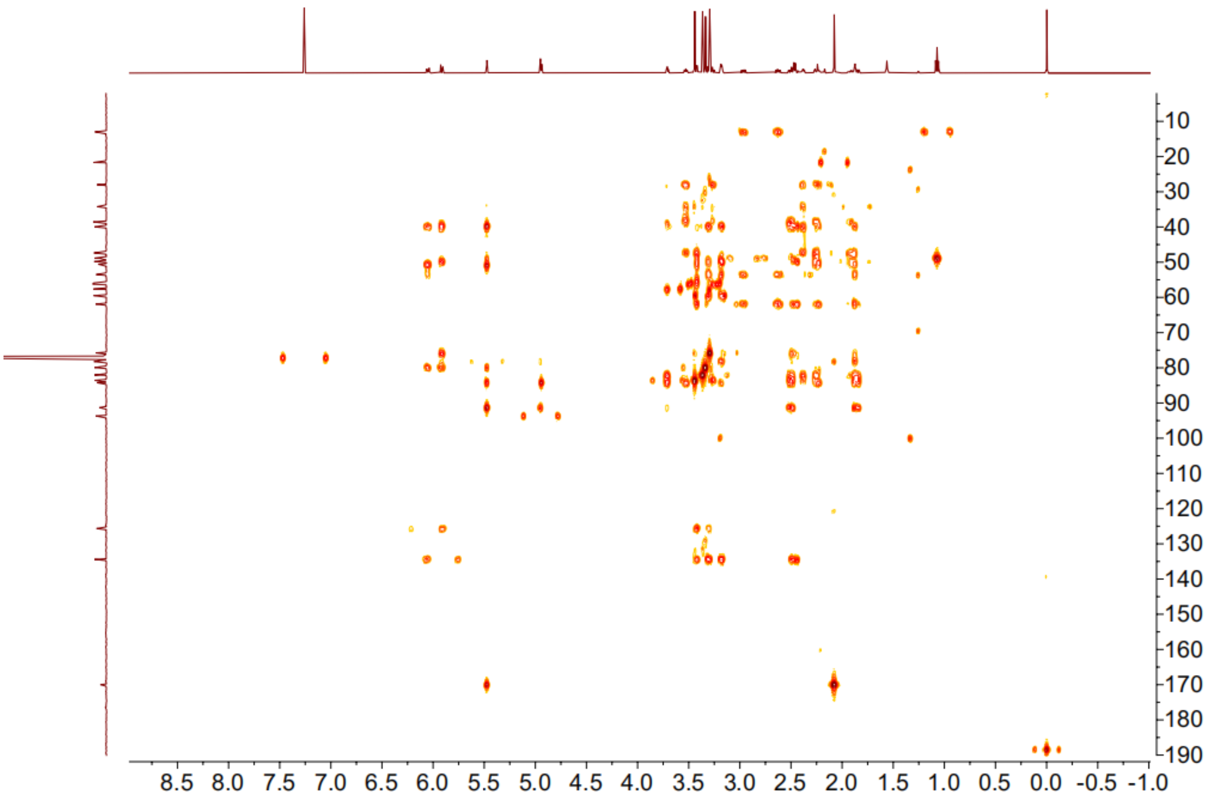

Figure S46: HMBC spectrum of compound 6

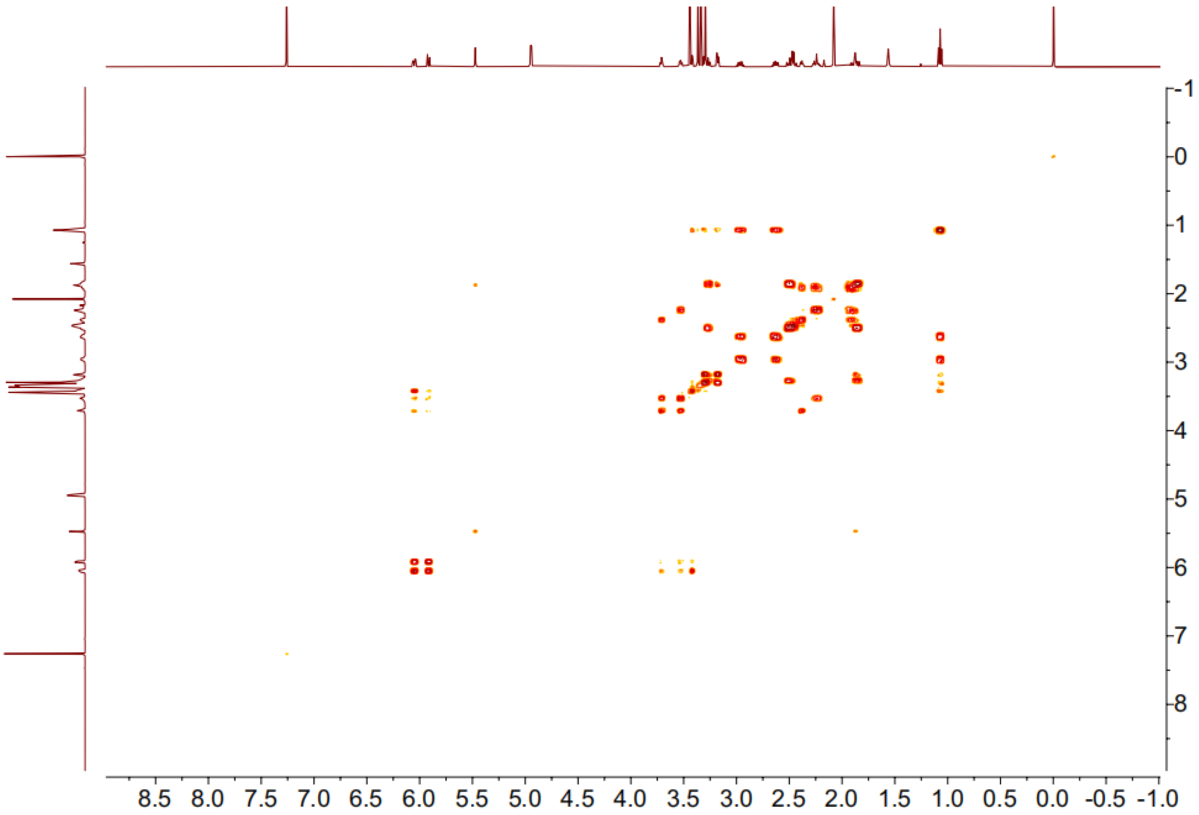

Figure S47: ^{1}H-^{1}H COSY spectrum of compound 6

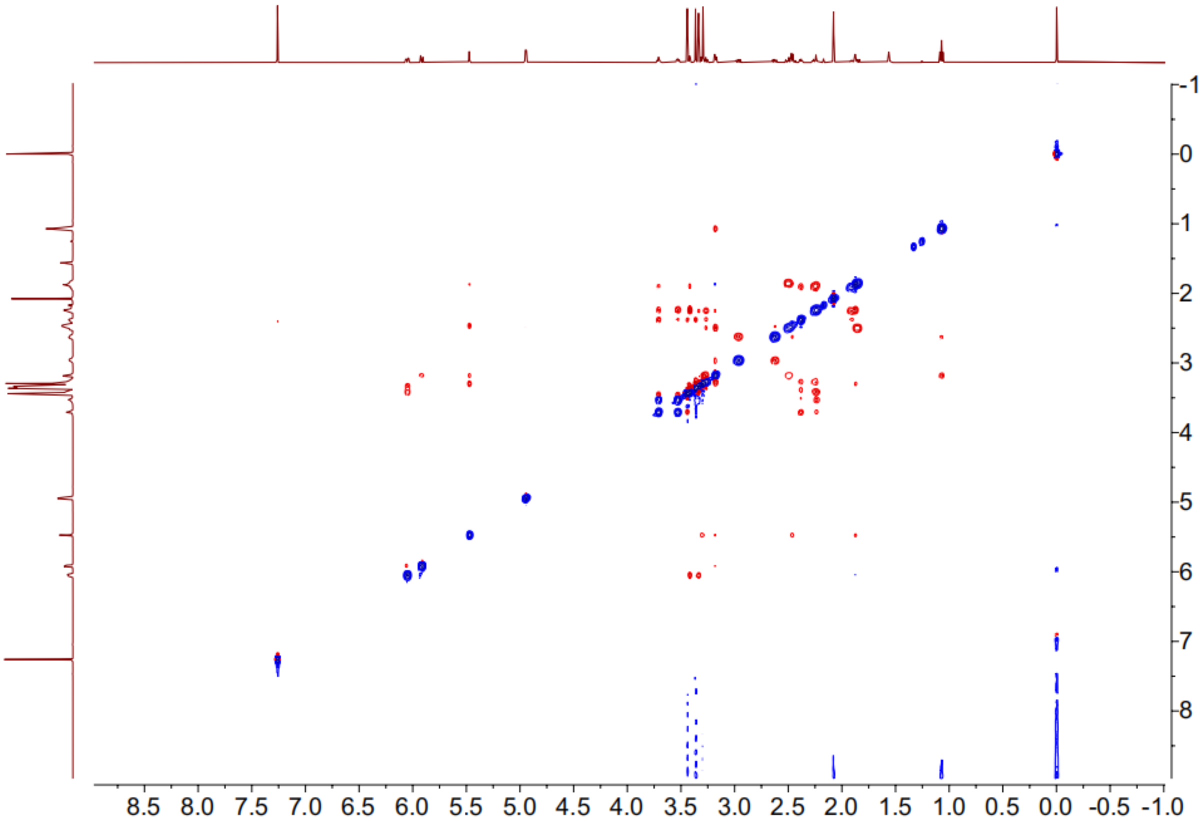

Figure S48: NOESY spectrum of compound 6

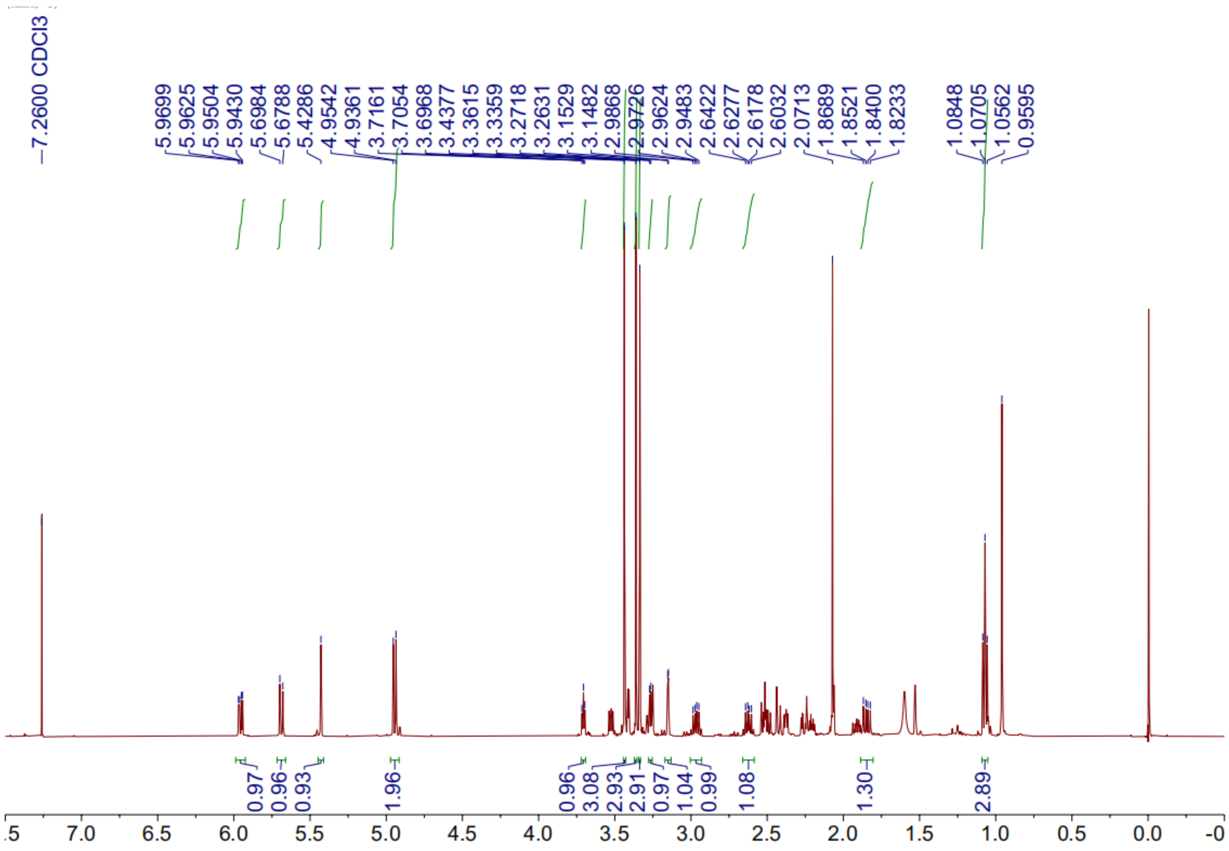

Figure S49: ^{1}H-NMR (500 MHz, $CDCl_3$) spectrum of compound 7

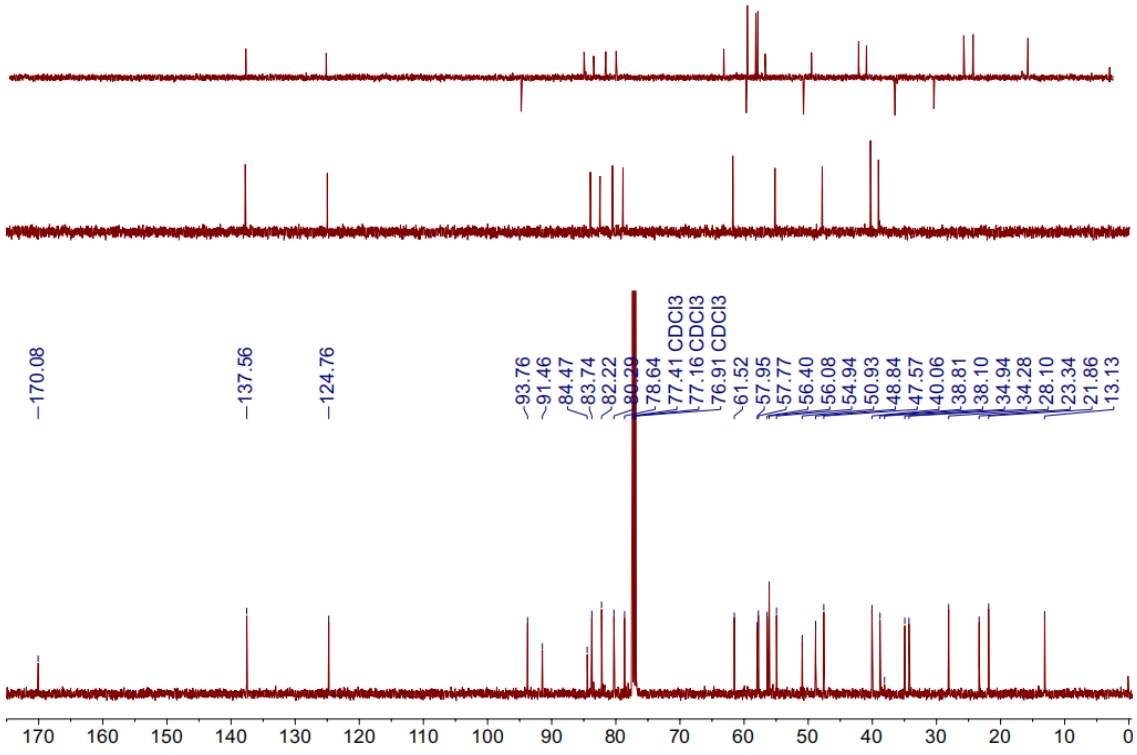

Figure S50: ^{13}C-NMR and DEPT (125 MHz, $CDCl_3$) spectrum of compound 7

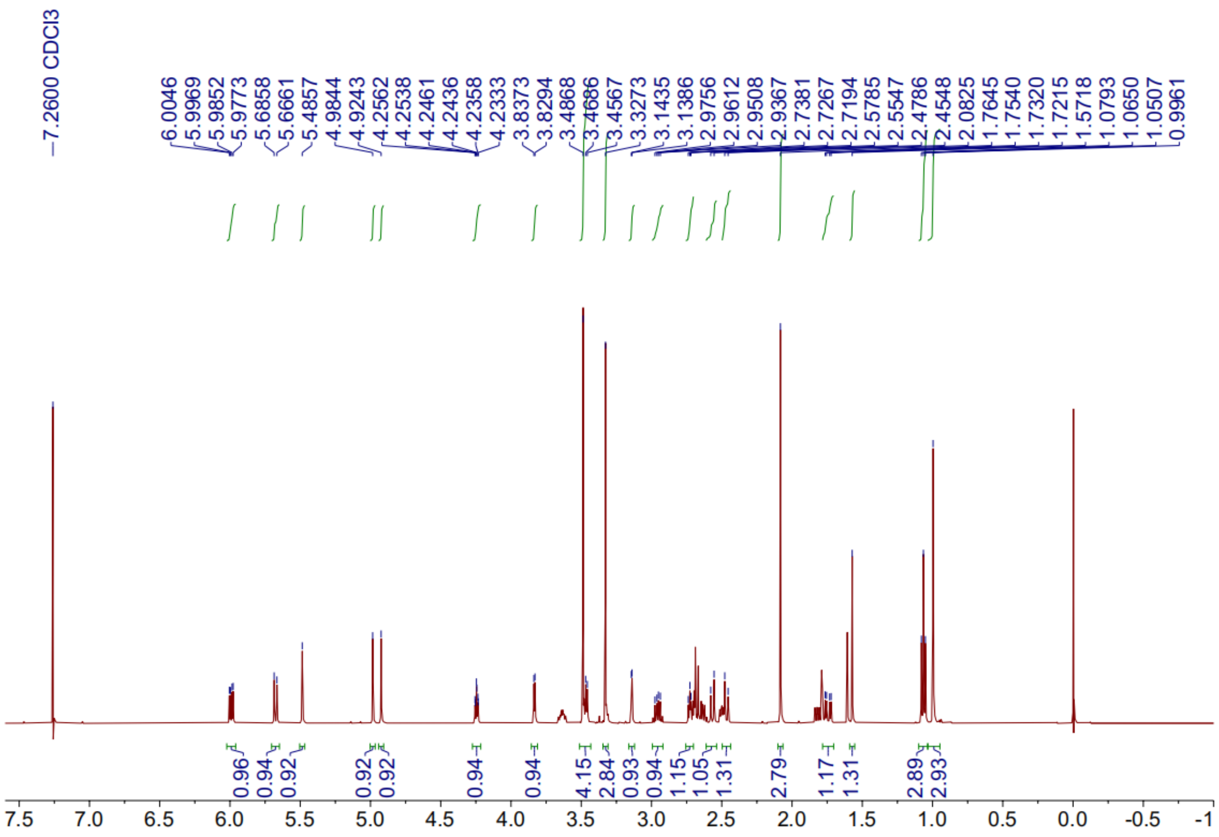

Figure S51: ¹H-NMR (500 MHz, $CDCl_3$) spectrum of compound 8

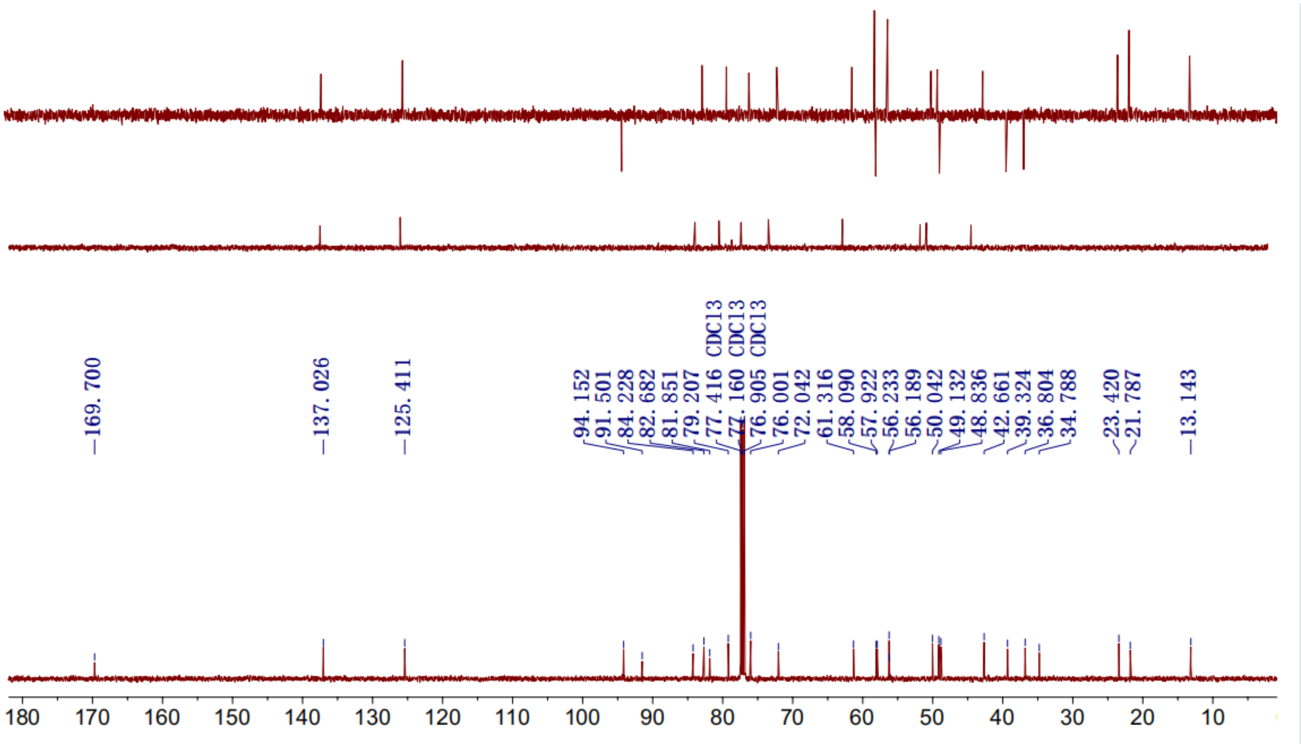

Figure S52: ¹³C-NMR and DEPT (125 MHz, $CDCl_3$) spectrum of compound 8

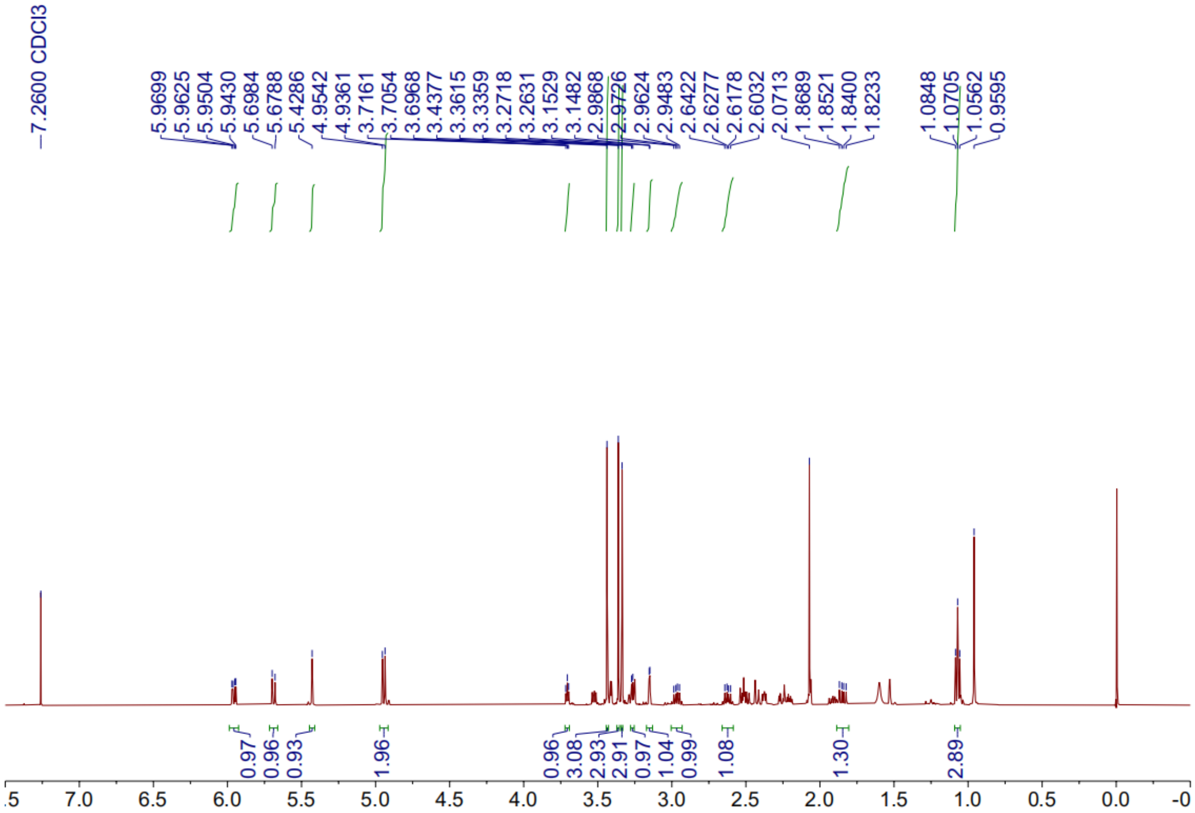

Figure S53: ^{1}H-NMR (500 MHz, $CDCl_3$) spectrum of compound 9

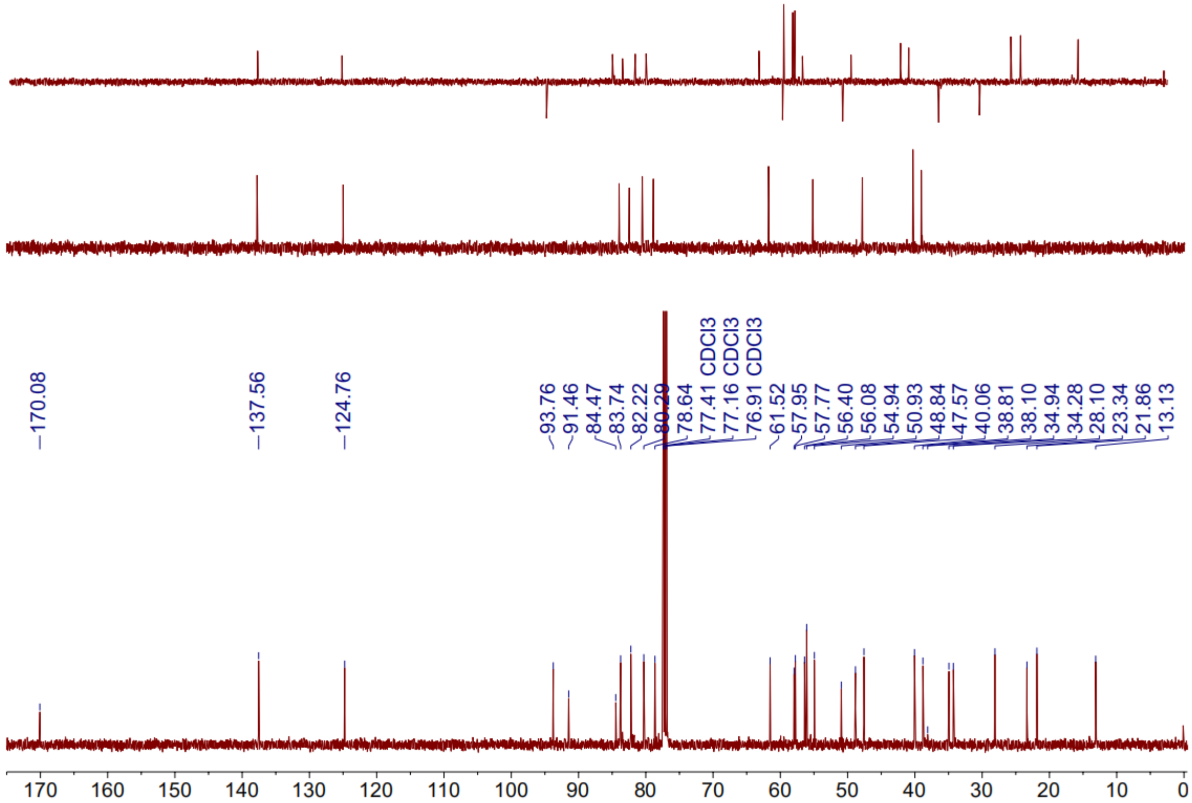

Figure S54: ^{13}C-NMR and DEPT (125 MHz, $CDCl_3$) spectrum of compound 9

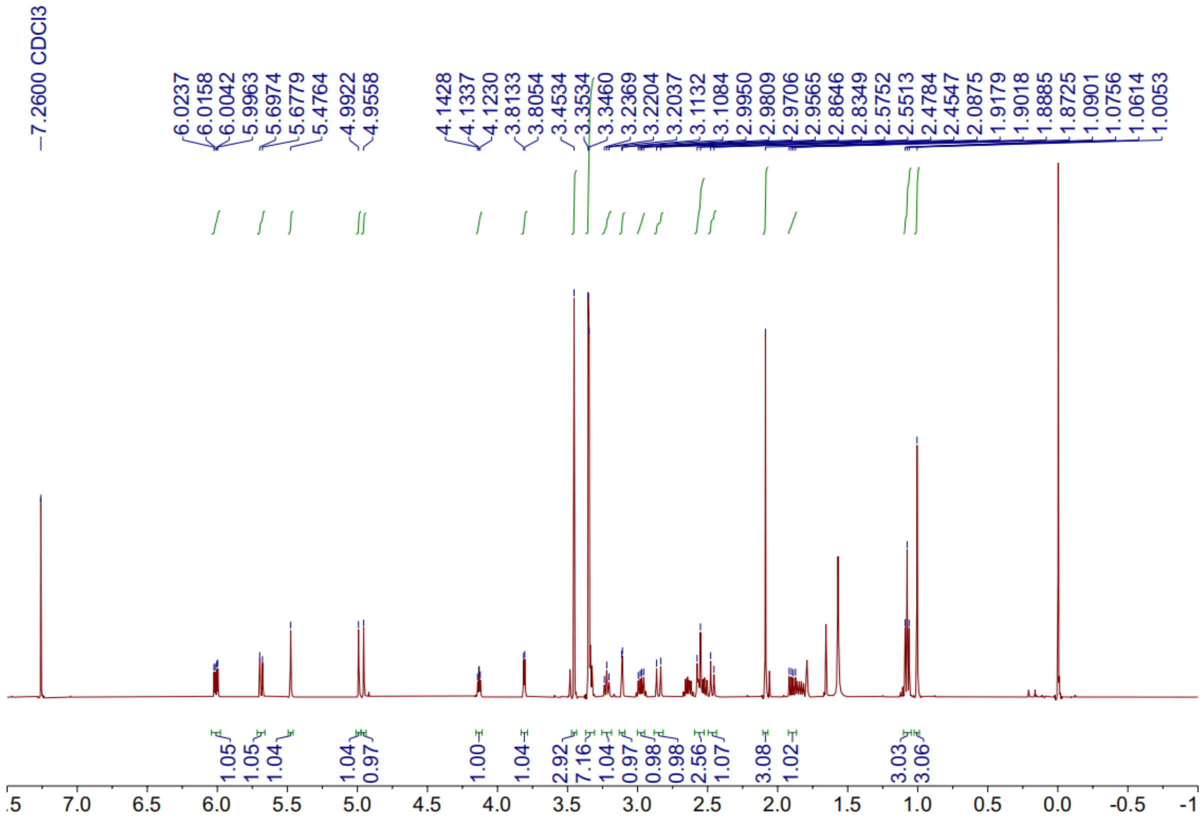

Figure S55: ^{1}H-NMR (500 MHz, CDCl$_3$) spectrum of compound 10

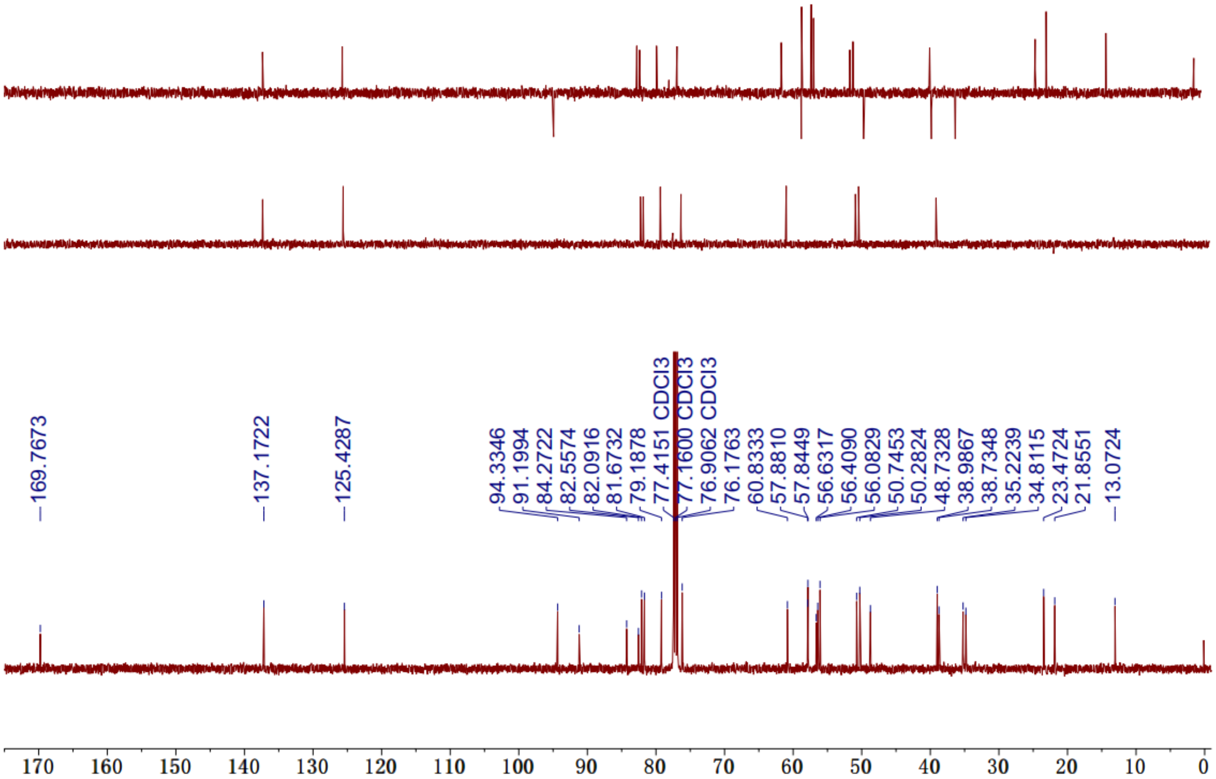

Figure S56: ^{13}C-NMR and DEPT (125 MHz, CDCl$_3$) spectrum of compound 10

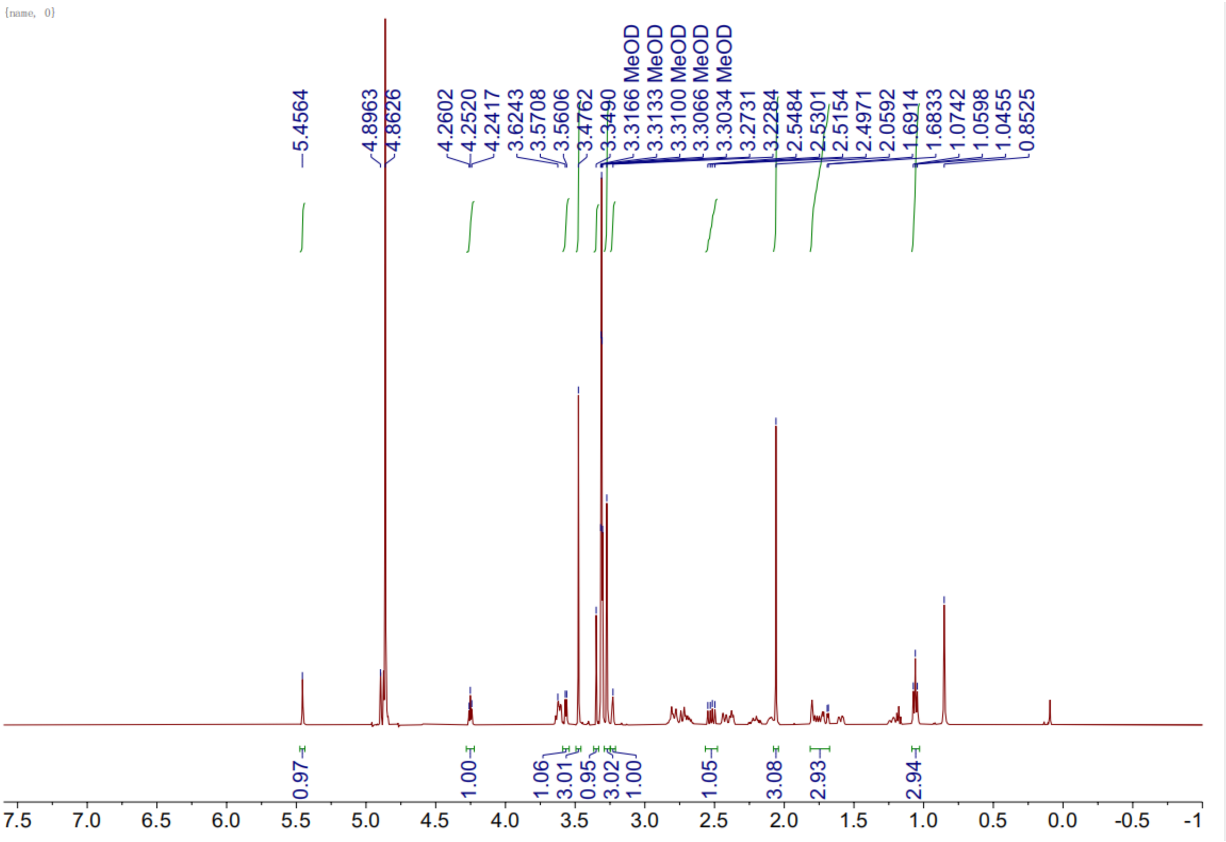

Figure S57: ^{1}H-NMR (500 MHz, $CDCl_3$) spectrum of compound 11

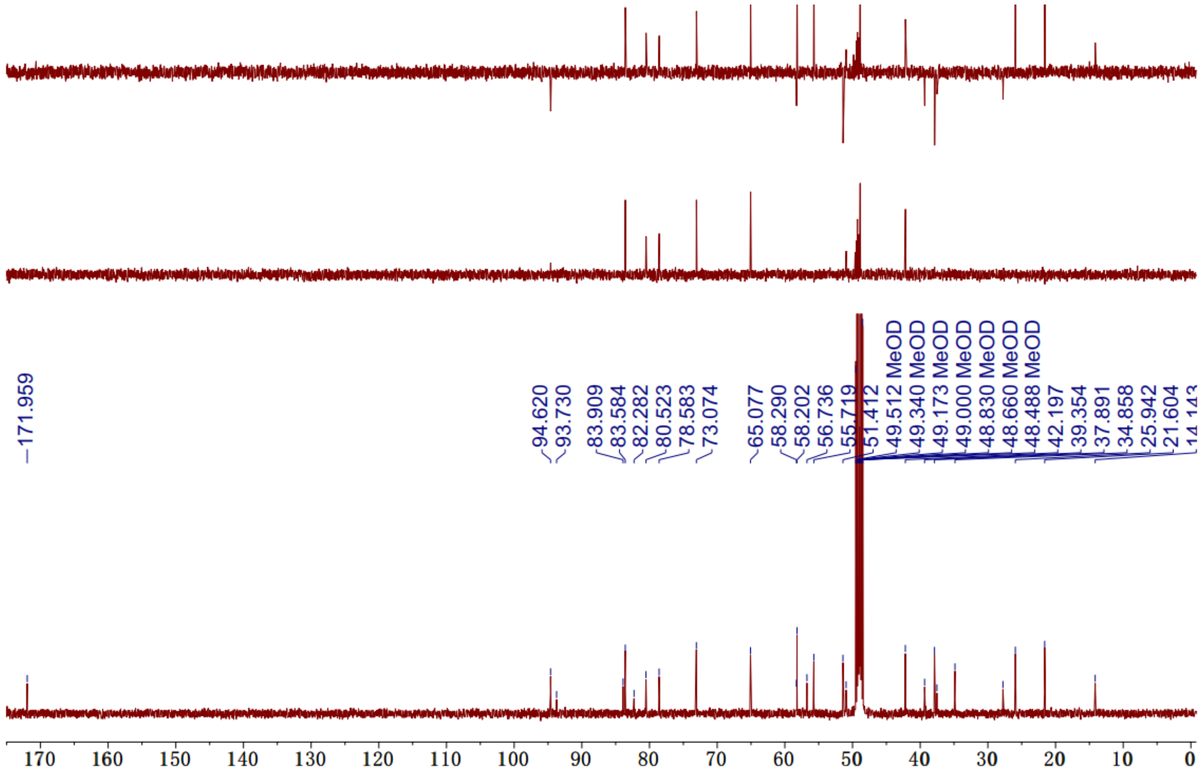

Figure S58: ^{13}C-NMR and DEPT (125 MHz, $CDCl_3$) spectrum of compound 11

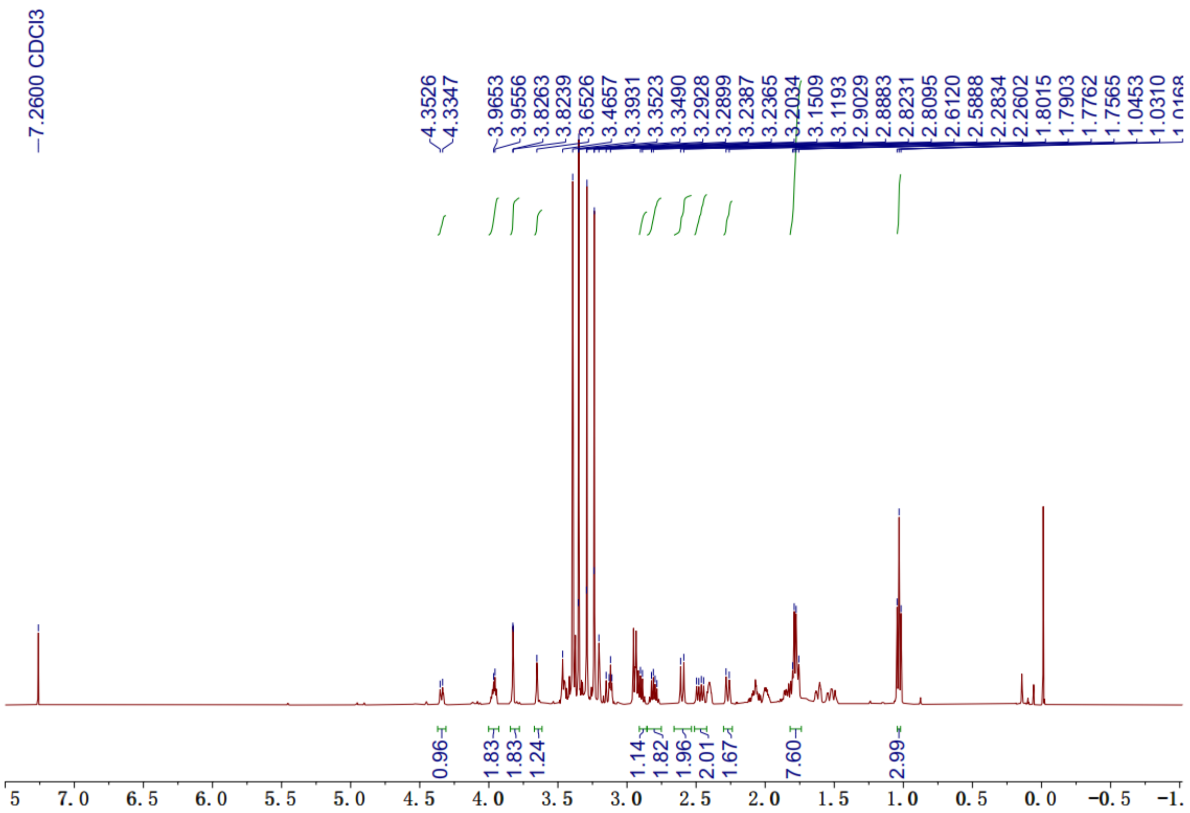

Figure S59: ^{1}H-NMR (500 MHz, $CDCl_3$) spectrum of compound 12

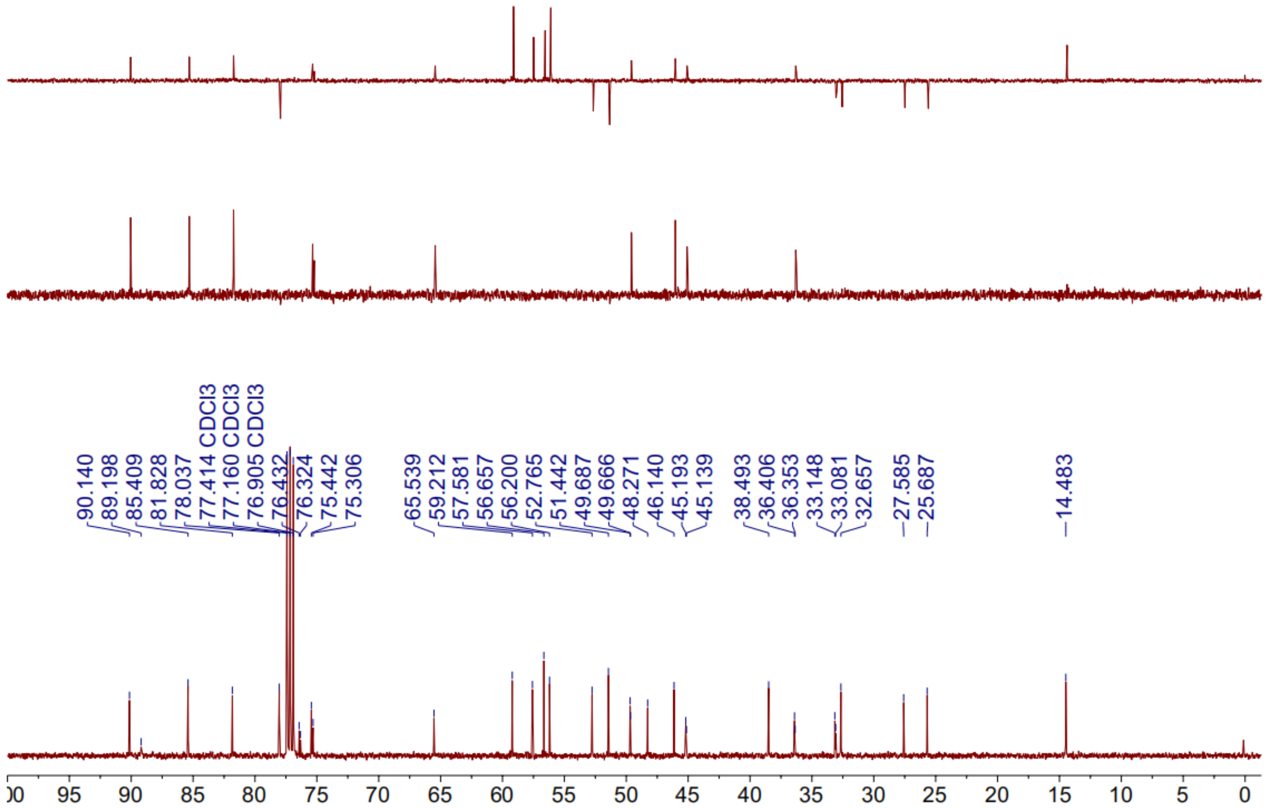

Figure S60: ^{13}C-NMR and DEPT (125 MHz, $CDCl_3$) spectrum of compound 12

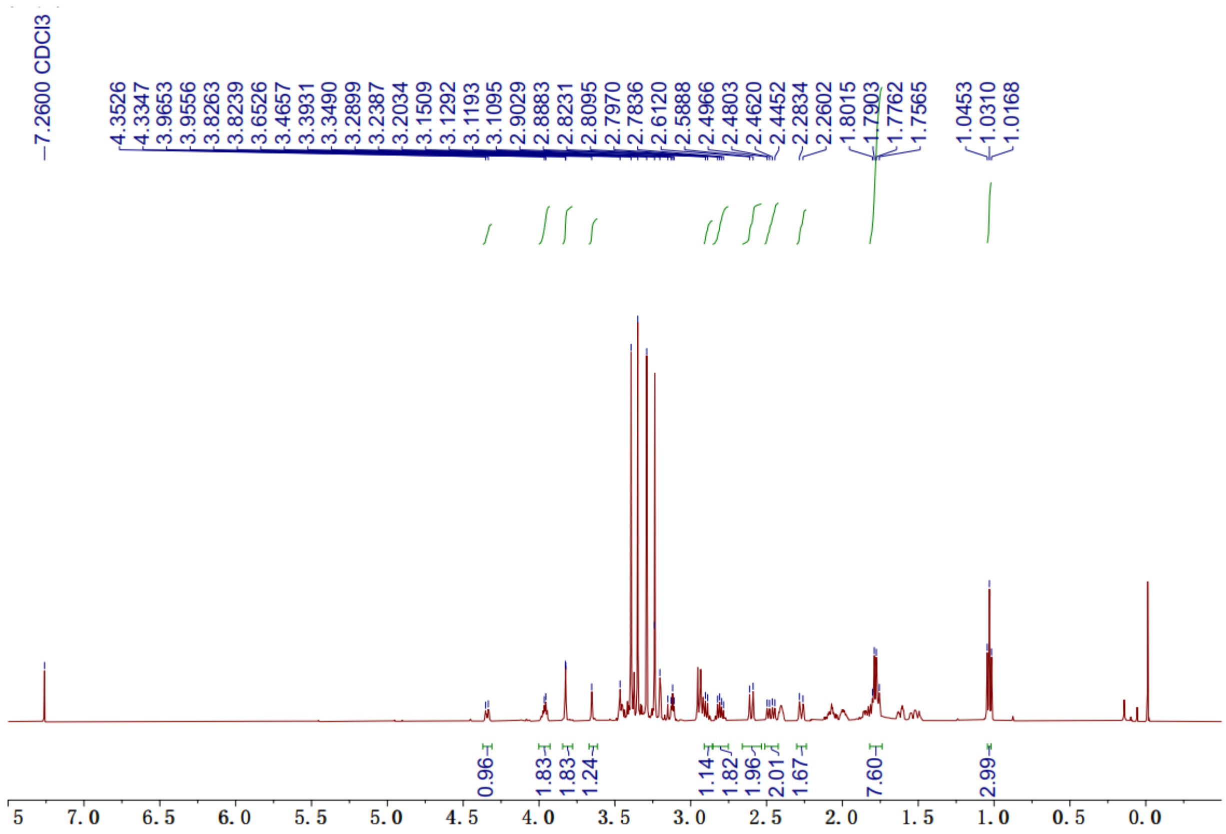

Figure S61: ^{1}H-NMR (500 MHz, $CDCl_3$) spectrum of compound 13

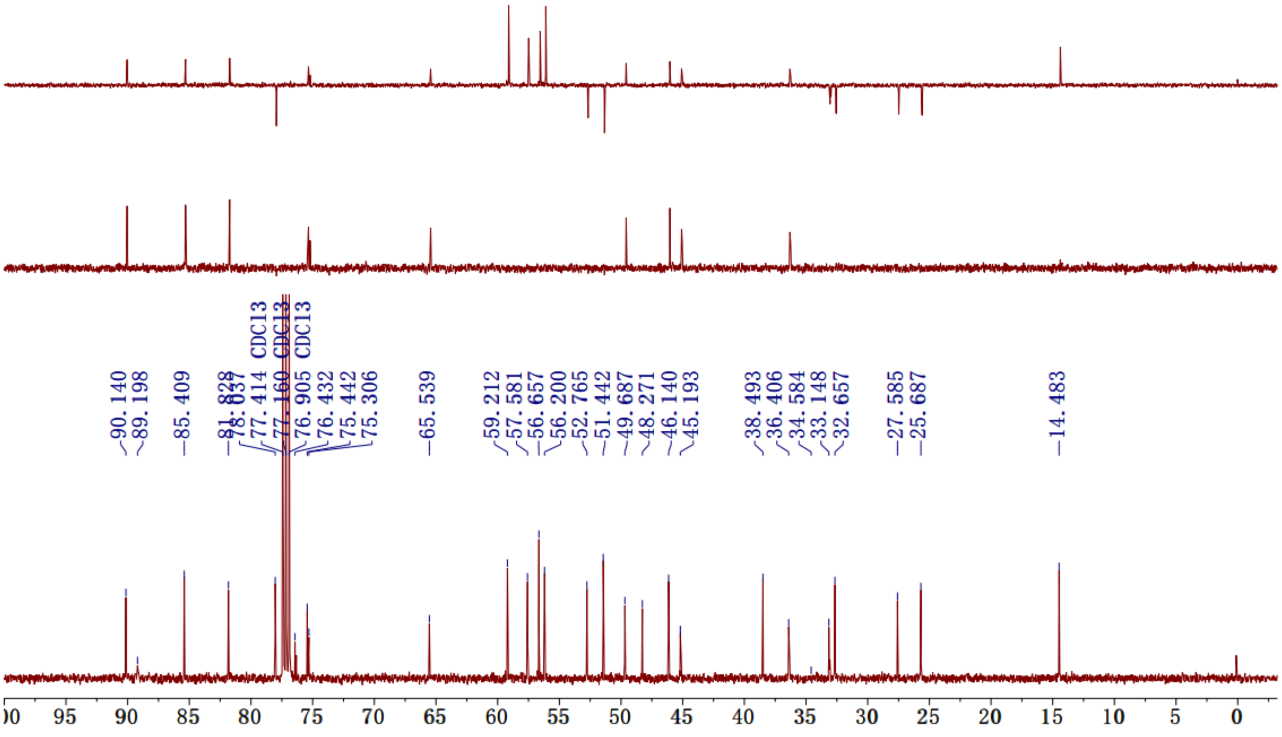

Figure S62: ^{13}C-NMR and DEPT (125 MHz, $CDCl_3$) spectrum of compound 13

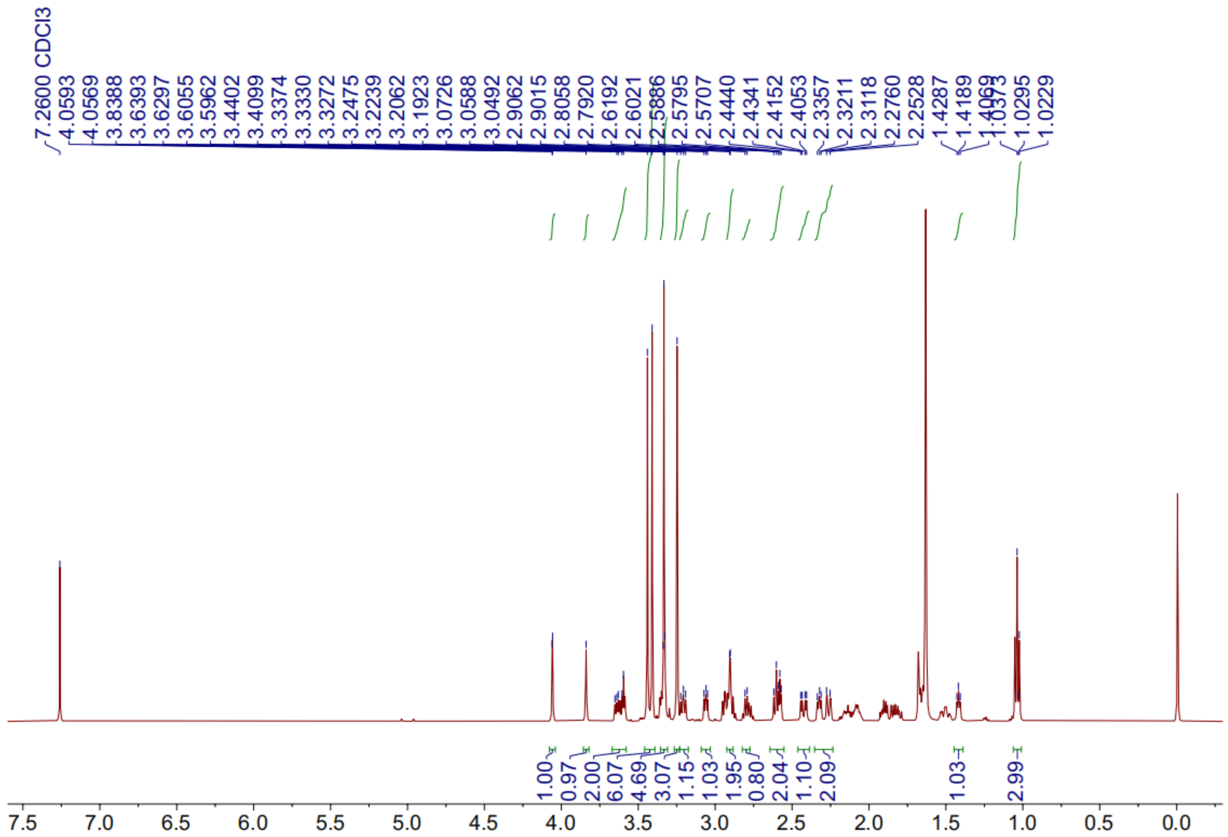

Figure S63: ^{1}H-NMR (500 MHz, $CDCl_3$) spectrum of compound 14

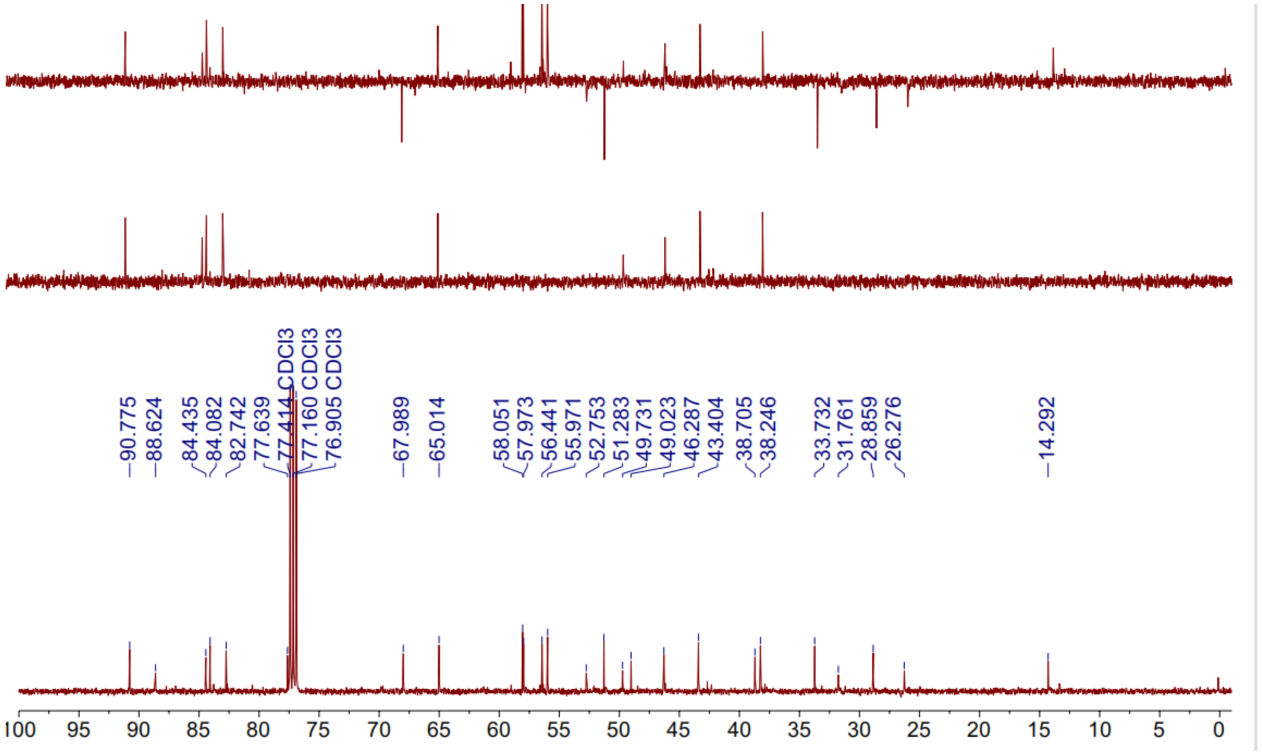

Figure S64: ^{13}C-NMR and DEPT (125 MHz, $CDCl_3$) spectrum of compound 14
